# Mechanical measurement of hydrogen bonded host–guest systems under non-equilibrium, near-physiological conditions†
†Electronic supplementary information (ESI) available: Synthetic and theoretical details as well as figures are available. See DOI: 10.1039/c7sc03044d



**DOI:** 10.1039/c7sc03044d

**Published:** 2017-07-31

**Authors:** Teresa Naranjo, Fernando Cerrón, Belén Nieto-Ortega, Alfonso Latorre, Álvaro Somoza, Borja Ibarra, Emilio M. Pérez

**Affiliations:** a IMDEA Nanociencia , C/Faraday 9, Ciudad Universitaria de Cantoblanco , 28049 , Madrid , Spain . Email: borja.ibarra@imdea.org ; Email: emilio.perez@imdea.org; b Nanobiotecnología (IMDEA-Nanociencia) , Unidad Asociada al Centro Nacional de Biotecnología (CSIC) , 28049 , Madrid , Spain

## Abstract

Decades after the birth of supramolecular chemistry, there are many techniques to measure noncovalent interactions, such as hydrogen bonding, under equilibrium conditions. As ensembles of molecules rapidly lose coherence, we cannot extrapolate bulk data to single-molecule events under non-equilibrium conditions, more relevant to the dynamics of biological systems. We present a new method that exploits the high force resolution of optical tweezers to measure at the single molecule level the mechanical strength of a hydrogen bonded host–guest pair out of equilibrium and under near-physiological conditions. We utilize a DNA reporter to unambiguously isolate single binding events. The Hamilton receptor–cyanuric acid host–guest system is used as a test bed. The force required to dissociate the host–guest system is ∼17 pN and increases with the pulling rate as expected for a system under non-equilibrium conditions. Blocking one of the hydrogen bonding sites results in a significant decrease of the force-to-break by 1–2 pN, pointing out the ability of the method to resolve subtle changes in the mechanical strength of the binding due to the individual H-bonding components. We believe the method will prove to be a versatile tool to address important questions in supramolecular chemistry.

## Abstract 2

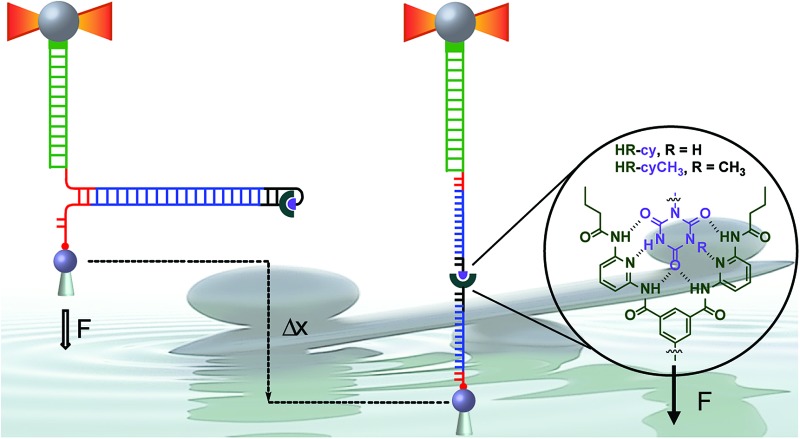
A new method to measure the mechanical strength of single hydrogen bonded host–guest systems under non-equilibrium conditions.

## Introduction

Hydrogen bonds are arguably the most prominent of noncovalent interactions, as the very foundations of life as we know it depend crucially on H-bonding.^1^ From a synthetic point of view, a wealth of host–guest systems,^2^ supramolecular polymers,^3^ and molecular machines^4^ based on H-bonding has been described.^5^ Unsurprisingly, measuring and understanding H-bonds has been the subject of intensive research efforts, and it still busies the scientific community.^6^ Most measurements of H-bonded systems are based on the determination of association constants,^7^ and therefore provide Δ*G* data obtained under equilibrium conditions, and are the average over very many interactions, in the order of Avogadro's number. As molecules in an ensemble are not synchronized, these data are not necessarily related to the behaviour of a single molecule under non-equilibrium conditions.^8^ In addition, the growing interest in molecular nanotechnology has increased the need for knowledge regarding molecular stability and bond mechanical strength from a single-molecule perspective and under near-physiological aqueous conditions. These conditions are required for the proper operation of next generation hybrid molecular motors; biological mechano-enzymes modified with synthetic molecules in order to control their activity and motion at the molecular level.^9^

The development of methods to manipulate individual molecules in the last couple of decades offers the opportunity to directly observe single molecule events for the first time.^10^ Scanning probe-microscopy methods (SPM) have been particularly successful for the observation of noncovalent interactions in organic solvents.^11^ However, under aqueous conditions, H-bonds are typically disrupted by forces of a few piconewtons (pN). For instance ∼10–15 pN are required to pull apart the H-bonds holding the double helix of DNA.^12^ This force range is at the detection limit of classical SPM methods.^13^ In addition, the non-specific adsorption of the sample, typically used by these methods and, in many cases, the lack of a proper reporter, hinders the unambiguous identification of single molecule events. In the biological sciences, this problem is circumvented by using repetitions of the unit peptide under study, and discarding the initial distance changes (typically the first 30 to 75 nm) and associated force peaks, which are systematically overestimations.^14^

**Fig. 1:**
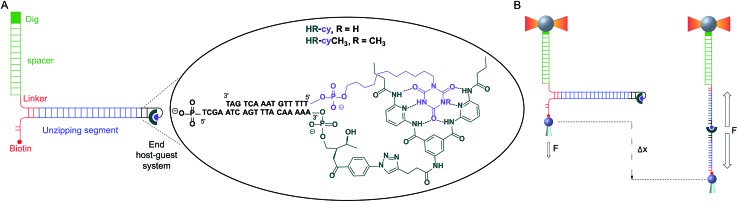
Schematic of experimental setup: (A) diagram illustrating the DNA construct (not to scale). One end of the main DNA unzipping segment (blue) was ligated to complementary DNA oligonucleotides bearing the receptor (HR, dark green) and the cyanuric acid (cy, purple). The other end of the unzipping segment was ligated through a short DNA linker (red) to a dsDNA (2686 bp) labelled with digoxigenin (Dig, green). The 5′ end of the linker was labelled with biotin. The insert shows the structure of the HR–cy couple and the sequence of the complementary DNA oligonucleotides. (B) A single DNA construct was tethered to functionalized beads *via* Dig-antiDig (green) and biotin-streptavidin (red) connections: one strand of the unzipping segment was attached to a bead (grey sphere) held in the laser trap (dark yellow beams) and the complementary strand to a bead on top of a mobile micropipette (light grey cone). The dsDNA spacer (green) provides separation (∼1 μm) between the two attachment points. Pulling the beads in opposite directions promotes the unzipping of the DNA (Δ*x*) and allows applying controlled, axial mechanical force (*F*) to the host–guest couple located at one end of the unzipping segment.

Here, we describe a novel experimental set-up to unequivocally isolate single H-bonded host–guest systems and study their mechanical strength under non-equilibrium, near-physiological conditions, using optical tweezers (OT).^15^ The ability of OT to resolve forces as small as 0.1–1 pN makes this technique ideally suited to measure the mechanical strength of noncovalent interactions in aqueous solutions. In order to unambiguously identify single host–guest interactions we use a hairpin-like DNA construct as a ‘single-molecule reporter’. The well-defined mechanical properties of DNA make this biological polymer an excellent reporter for identification and manipulation of single molecules.^12,16^ As a model H-bonded system, we focused on the receptor for barbiturates developed by Hamilton^17^ and a cyanuric acid derivative as guest (HR–cy in Fig. 1A and ESI†), which features six strong H-bonds. We based our choice on several reasons: (1) the system has been thoroughly studied in bulk; (2) benchmark data using SPM for a similar system in organic solvents are available;^18^ (3) the synthesis of the building blocks is well-known and versatile, for instance it is possible to block one of the cyanuric acids' H-bond donors by methylation (HR–cyCH_3_ in Fig. 1A).

## Results and discussion

To study the mechanical strength of the H-bonding of a single host–guest system, the receptor (HR) and the cyanuric acid were linked covalently to the 3′- and 5′-termini, respectively, of self-complementary DNA oligonucleotides (Fig. 1A and ESI†). Briefly, an amino alkyl chain-controlled pore glass (LCAA-CPG) solid support was modified with the HR (compound 9, ESI†) and used as a ground to synthesize, in the 3′ to 5′ direction, one of the oligonucleotides (Fig. 1A, oligonucleotide 6 in ESI†). The 5′-terminus of the complementary oligonucleotide (Fig. 1A, oligonucleotides 7 and 8 in ESI†) was linked covalently to a cyanuric acid phosphoramidite (compound 11 and 15, ESI†) on the last step of a solid support DNA chemical synthesis. The oligonucleotide sequences were designed to provide a 5′ protruding end after annealing (Fig. 1A), which was used to ligate the modified oligonucleotide couple to one end of a 410 bp double-stranded DNA (dsDNA) molecule (or unzipping segment). The other end of the unzipping segment was modified at the 5′-terminus with a single biotin and at the 3′-terminus with a dsDNA spacer (∼1 μm) finished with multiple digoxigenins (Dig) (Fig. 1A and ESI†).^19^ We used a counter propagating dual-beam optical tweezers instrument^20^ to unzip mechanically individual DNA constructs tethered between an optically trapped anti-Dig-coated bead and a streptavidin-coated bead immobilized on top of a micropipette (Fig. 1B and ESI†). This configuration allows applying controlled directional mechanical force to the opposite strands of the DNA construct and to determine ultimately the mechanical strength of the transient, out of equilibrium, H-bond interactions holding together the single host–guest couple located at the end of the unzipping segment. Therefore, the DNA construct was designed to pull the HR–cy couple in an axial direction along the extension of the host–guest couple (Fig. 1B).

**Fig. 2:**
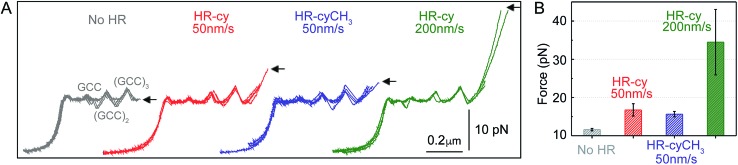
Rupture forces of the HR–cy couple. (A) Representative experiments showing the force extension curves of DNA constructs harbouring at their ends the HR–cy (red, 50 nm s^–1^, green 200 nm s^–1^) or HR–cyCH_3_ (blue, 50 nm s^–1^) couples. The mechanical unzipping of DNA constructs without the HR component is shown in grey. Four independent curves are shown in each case (see ESI† for additional curves). The positions of the GCC clusters are revealed as peaks in the unzipping pattern. Arrows indicate the force and position of the rupture events. (B) Average rupture forces for DNA constructs without the HR–cy couple (grey), with the HR–cy couple (red, 50 nm s^–1^, green 200 nm s^–1^) and with the HR–cyCH_3_ couple (blue, 50 nm s^–1^). Error shows standard deviations; see the main text for populations. Data were taken at 100 Hz (22 ± 1 °C).

Under near-physiological NaCl and pH conditions (Tris–HCl 20 mM pH 7.5, 50 mM NaCl) mechanical unzipping of a single DNA construct started at a force of ∼12 pN (Fig. 2A). The characteristic force–extension curve of the molecule permitted the unequivocal identification of single attachments between the beads.^12,21^ The initial and final extensions of the DNA constructs corresponded to their expected lengths before and after unzipping, respectively.^22^ The unzipping segment contained three guanine-cytosine (GC) base pair clusters with 1 to 3 repetitions of the GCC sequence, separated by a ∼100 nucleotide low-GC content, the three GCC clusters are clearly visible as distinct increments in force as we move from the initial GCC cluster to (GCC)_2_ and (GCC)_3_ (Fig. 2A). These sequences were used as fiducial markers to align independent force–extension curves (ESI†) and further identify the end of the DNA unzipping segment where the host–guest interactions should occur.

Mechanical unzipping of the DNA was typically carried out with a pulling rate of 50 nm s^–1^. When the HR component of the host–guest system was not included at the end of the unzipping segment, disassembly was completed shortly after unzipping of the last (GCC)_3_ position at an average force of 11.5 ± 0.3 pN (number of experiments, *N* = 20, Fig. 2B). However, when the HR–cy host–guest system was included, after full unzipping of the DNA segment the force increased to 16.7 ± 1.6 pN (*N* = 100, 12% of which showed unequivocal signs of binding), reflecting the mechanical strength of the H-bonding between the HR–cy couple under these experimental conditions (Fig. 2B). Disruption of one of the H-bonding interactions by a methyl group (HR–cyCH_3_) decreased the rupture force to 15.6 ± 0.7 pN (*N* = 100, 10%, Fig. 2B). We note that although mechanical stability is not necessarily related to thermodynamic stability,^23^ our results are in agreement with the effect of these modifications on the thermal stability of oligonucleotide duplexes in the bulk (see the ESI†). We confirmed with a 95% probability than the difference between the rupture forces measured for the HR–cy and HR–cyCH_3_ couples is statistically significant (ESI†). In addition, increasing the pulling rate (from 50 nm s^–1^) to 200 nm s^–1^ increased the average rupture force for the HR–cy system to 34 ± 9 pN (*N* = 100, 9%, Fig. 2B), as expected for a system under non-equilibrium conditions.^11*d*^ We note that the measured average rupture forces for the HR–cy and HR–cyCH_3_ couples are significantly smaller than the average breaking force of the DNA-beads interactions under the same experimental conditions, 73 ± 10 pN (*N* = 10) (ESI†).

These results highlight the remarkable ability of our method to resolve subtle changes in the mechanical strength of the complex due to the H-bond components. However, we note that the mechanical stability of the HR–cy couple depends on the location of the transition state along the mechanical reaction coordinate used in our experiments, the height of the barrier and the loading rate.^23,24^ Therefore, the H-bonds of the host–guest system are probably not equivalent in terms of resistance to the applied force, and our measurements reflect an average for the mechanical strength of the HR–cy couple. In fact, the difference in the rupture force between the HR–cy and HR–cyCH_3_ couples (1–2 pN) suggests that the applied force is distributed over the whole binding site of the host–guest couple and is not representative of a single H-bond.

Previously reported average rupture forces for the HR–cy system were measured at ∼170 pN with Atomic Force Microscopy (AFM) at a pulling rate of 250 nm s^–1^ in an organic solvent.^18^ As discussed above, the discrepancy in experimental results may arise from differences in the pulling geometry, effective pulling rates, contact forces^25^ and/or from an enhancement in binding strength of H-bonded complexes in organic solvents compared to aqueous environment.

**Fig. 3:**
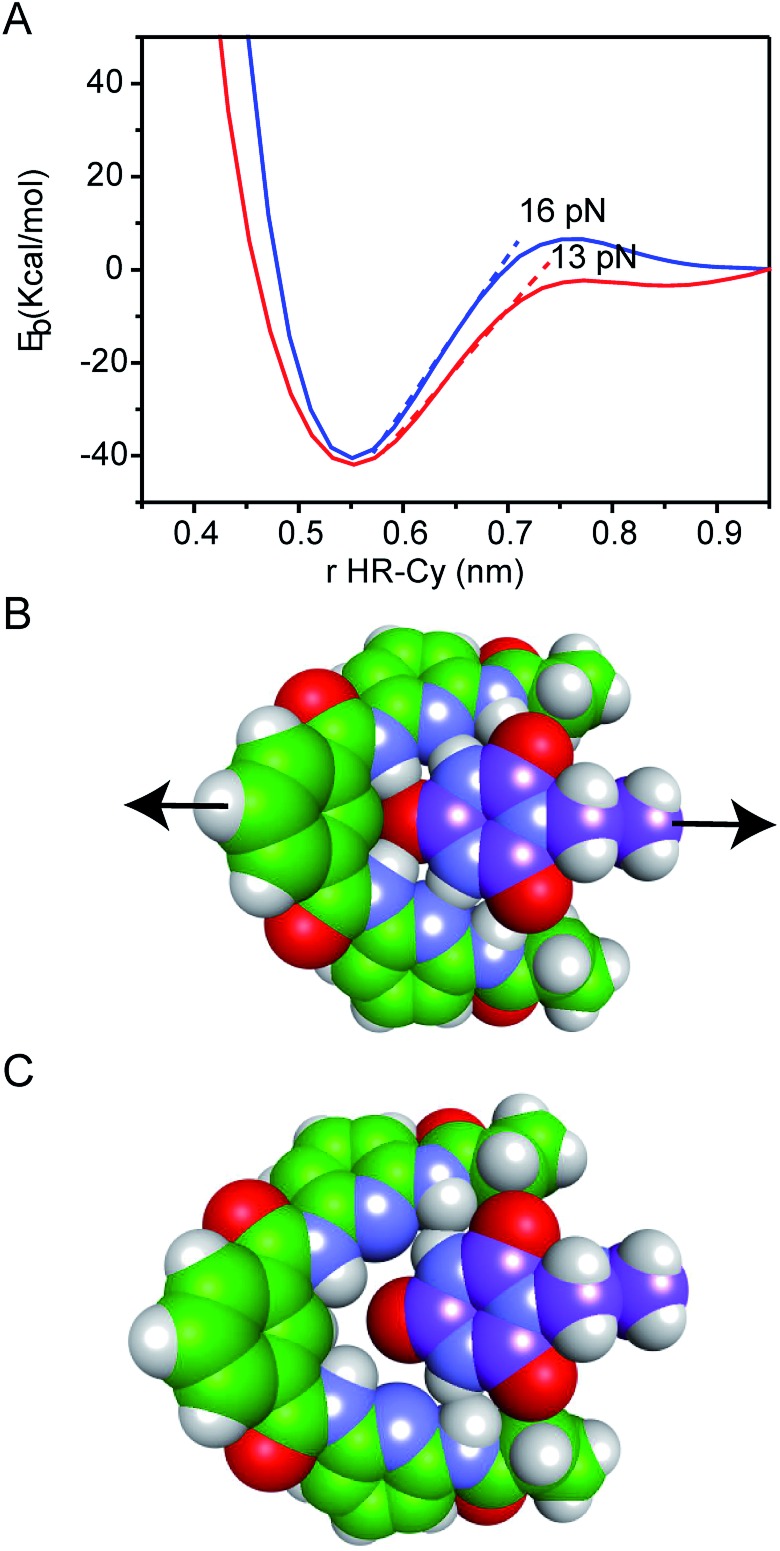
(A) Potential energy curves for HR–cy (blue) and HR–cyCH3 (red) employing IEF-PCM water solvation model. Energy-minimized molecular models showing the geometry of the (B) bound and (C) unbound HR–cy system.

Our experimental results were corroborated by DFT calculations. The potential energy curves (PECs) for HR–cy and HR–cyCH_3_ couples were calculated performing single point energy calculations at different distances in an axial direction, along the extension of the host–guest couple (ESI†). Specifically, we increase the host–guest distance 50 times with a variation of 0.2 Å between them. The long range corrected ωB97xD density functional^26^ was employed in combination with the basis-set 6-31g(d,p) and the IEF-PCM water solvation model. This methodology has been chosen following the literature in the field.^27^ The PECs for HR–cy and HR–cyCH_3_ systems and the optimized structures for the completely bonded and non-interacting states in water are displayed in Fig. 3.

In Fig. 3A, we observe that at large distances, interactions are negligible (*E*_b_ = 0) but attractive interactions grow as host and guest approach each other, reaching a state of equilibrium where the net force (the sum of both attractive and repulsive components) is zero. The PECs indicate the energy of the host–guest interaction based on the depth of the potential well, and the force (*F*) is the slope of the curve, according to: *F* = –d*E*_b_/d*r*, where *E*_b_ is the binding energy and *r* the distance between host and guest. At equilibrium, the binding energy for the systems HR–cy and HR–cyCH_3_ are calculated to be 40.51 and 41.92 kcal mol^–1^ respectively, which are expected binding energies for systems with six and five strong hydrogen bonds in aqueous conditions.

At short distances from the equilibrium state (0–0.03 nm) the potential energy is not affected by a change in the intermolecular distance, but at larger values of *r* a change in the slope is observed. From this point to the next change in slope, where the cyanuric acid is completely dissociated from the HR, a value of 16 pN for the force is obtained (Fig. 3). This value is directly related with the force required to split the couple, and is in good agreement with our experimental data. In the case of the HR–cyCH_3_ system, the calculated force was 13 pN, a noticeably lower value than HR–cy, following the same tendency of ours experimental results. We also calculated the rupture force using 1,2-dichlorobenzene as model of organic solvent (see ESI†). We observe a slight increase in the force: 17 pN for HR–cy system and 14 pN for HR–cyCH_3_. DFT calculations corroborate that the average rupture forces measured in our work under aqueous conditions are in the correct order of magnitude, and support a relatively minor role of solvent composition in the breaking of an already formed H-bonded host–guest couple.

## Conclusions

In conclusion, we have developed a new method to measure the average mechanical strength of H-bonded supramolecular complexes, at the single-molecule level, under non-equilibrium conditions. Our method expands the capabilities of previously described SPM methods by exploiting the force resolution of optical tweezers (0.1–100 pN), which allows measuring the characteristic strength of H-bonds under near-physiological conditions, and by including a DNA reporter that guarantees that the force measurements refer to a single binding event. The modularity and addressability of the DNA construct described here also opens up the possibility of targeting synthetic host–guest couples in controlled orientations and stoichiometry. In this case, we have focused on aqueous conditions, but the system is in principle adaptable to other sets of conditions. This renders our method a versatile and precise tool to address key questions in supramolecular chemistry, like the mechanical strength of hydrogen bonded systems, and its relationship with their thermodynamic stability.
