# Supplementary material for: Mechanical measurement of hydrogen bonded host–guest systems under non-equilibrium, near-physiological conditions
Source: Chem Sci. 2017 Jul 31;8(9):6037–41. doi: 10.1039/c7sc03044d (PMC5625567; doi:10.1039/c7sc03044d)
Supplement: Supplementary file 1 [file SC-008-C7SC03044D-s001.pdf]

Electronic Supplementary Material (ESI) for Chemical Science.
This journal is © The Royal Society of Chemistry 2017

Mechanical measurement of hydrogen bonds under non-equilibrium, near-physiological conditions

SUPPORTING INFORMATION

Synthesis and characterization

General. All solvents were dried according to standard procedures. Reagents were used as purchased. All air-sensitive reactions were carried out under argon atmosphere. Flash chromatography was performed using silica gel (Merck, Kieselgel 60, 230-240 mesh, or Scharlau 60, 230-240 mesh). Analytical thin layer chromatographies (TLC) were performed using aluminium-coated Merck Kieselgel 60 F254 plates. NMR spectra were recorded on a BrukerAvance 400 (^{1}H: 400 MHz; ^{13}C: 100 MHz), spectrometer at 298 K, unless otherwise stated, using partially deuterated solvents as internal standards. Coupling constants (J) are denoted in Hz and chemical shifts (δ) in ppm. Multiplicities are denoted as follows: s = singlet, d = doublet, t = triplet, sx = sextuplet, m = multiplet, b = broad. Fast Atom Bombardment (FAB) and Matrix-assisted Laser desorption ionization (coupled to a Time-Of-Flight analyzer) experiments (MALDI-TOF) were recorded on a VS AutoSpec spectrometer and a Bruker ULTRAFLEX III spectrometer, respectively.

1. Synthesis of the Hamilton receptor derivative
2. Preparation of modified solid supports
3. Cyanuric acid derivate synthesis
4. Oligonucleotide synthesis and analytical data of oligonucleotides
5. Mass spectral data of synthetized oligonucleotides
6. Melting temperatures of duplexes
7. Synthesis of DNA construct
8. Optical Tweezers experiments
9. Experimental force-extension curves
10. Statistical analysis
11. Computational details
12. Z-matrix of molecular optimized geometries
13. References

1. Synthesis of the Hamilton receptor derivative

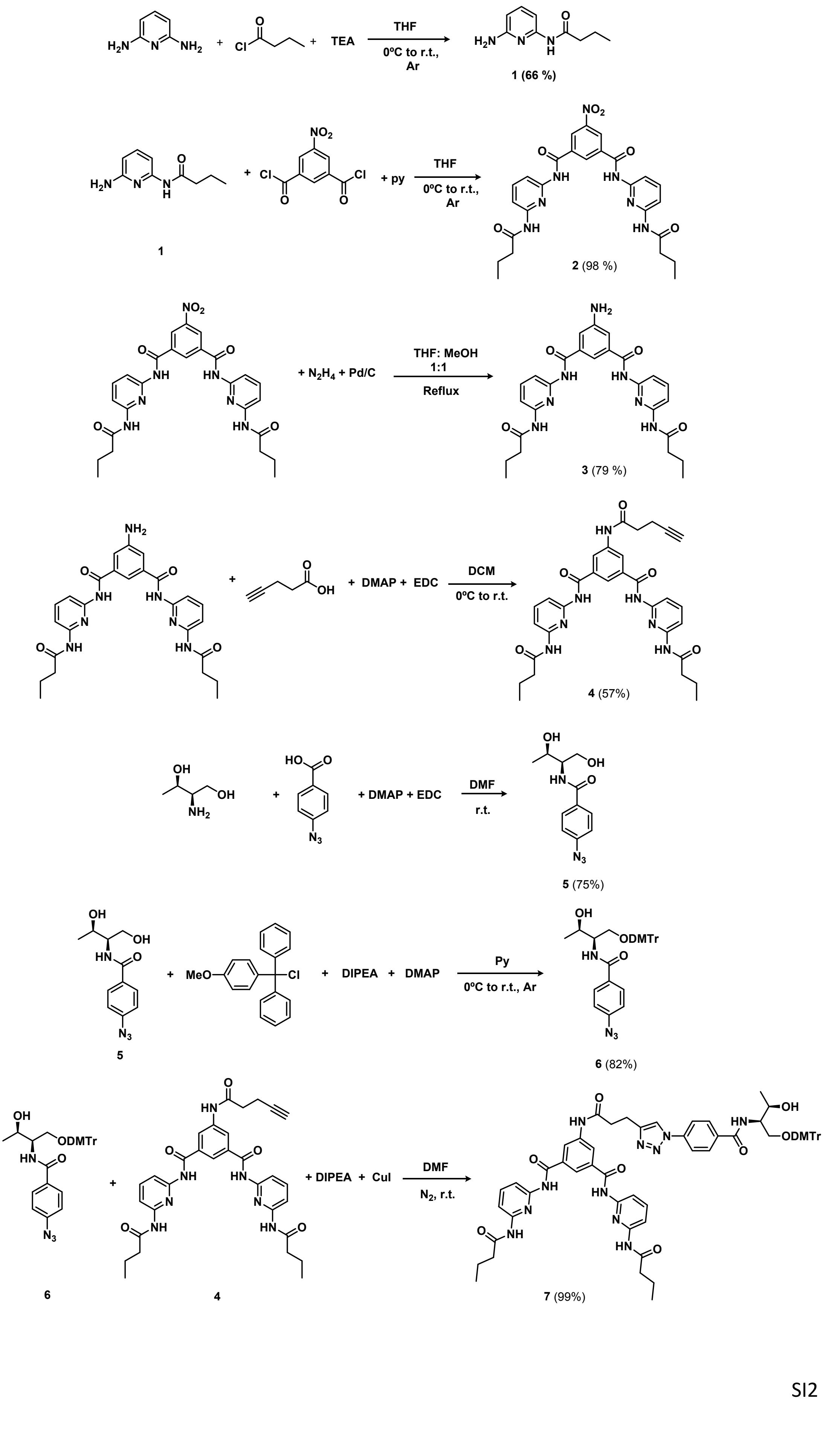

Compound 1: A solution of butyryl chloride (2 g, 18.8 mmol) in dry THF (10 mL) was added to a solution of 2,6-diaminopyridine (2.05 g, 18.8 mmol) and trimethylamine (1.9 g, 18.8 mmol) in dry THF (20mL) at 0 ºC under nitrogen atmosphere over a period of 2h. The solution was stirred overnight, at room temperature; the residue was filtered off and the solvent removed under reduced pressure. The product was purified by column chromatography to give **1** as yellow solid, 2.23 g, 66%. ^{1}H NMR ($CDCl_3$): δ= 7.65 (s, 1H), 7.54 (br d, *J* = 7.8 Hz, 1 H), 7.44 (t, *J* = 7.9 Hz, 1 H), 7.44 (t, *J* = 7.9 Hz, 1 H), 7.23 (d, *J* = 8 Hz, 1 H), 4.29 (br s, 2H), 2.31 (t, *J* = 7.4 Hz, 2 H), 1.73 (s, *J* = 7.4 Hz, 2 H), 0.98 (t, *J* = 7.4 Hz, 3 H) .This data is in accordance with *Chem. Eur. J.* 2007, **13**, 5466.

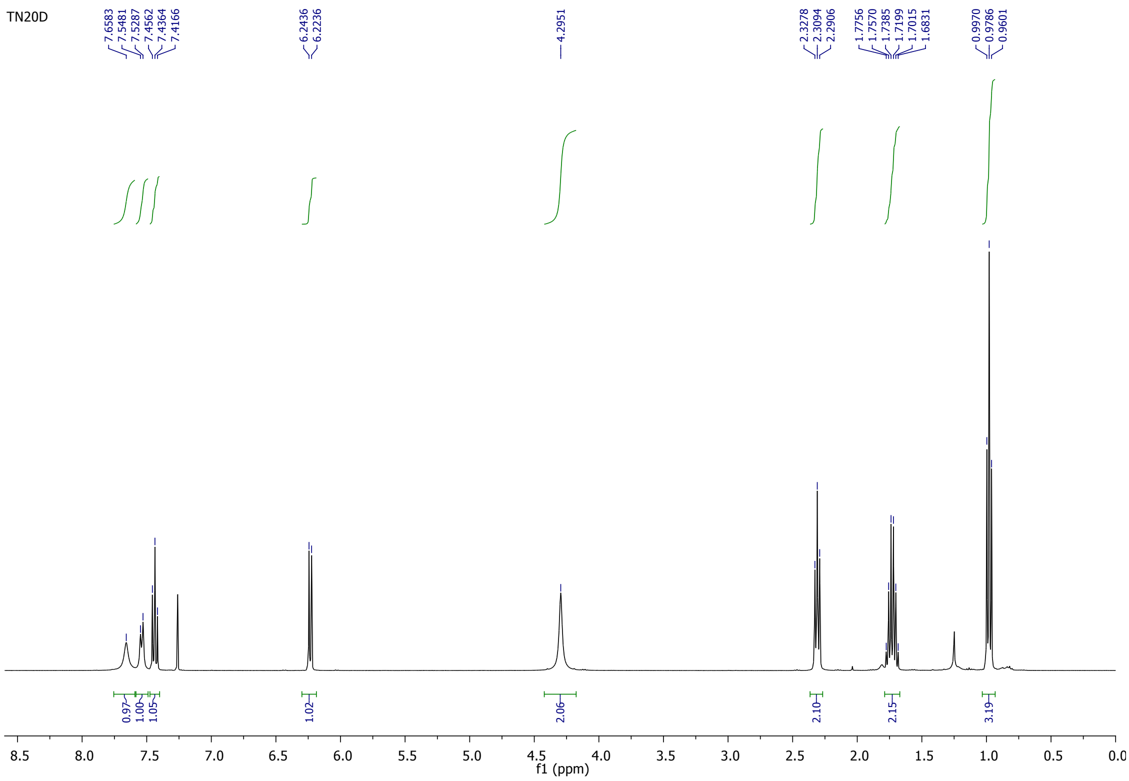

Compound 2. A solution of 5-nitroisophthaloyl dichloride (0.9 g, 3.62 mmol) in dry THF was added dropwise to a solution of monosubstituted diaminopyridine **1** (1.5 g, 7.24 mmol) and trimethylamine (0.7 g, 7.24 mmol) in dry THF (15 mL) at 0ºC under argon atmosphere. The solution was stirred at room temperature overnight, the residue filtered off and the solvent removed under reduced

pressure. Purification by column chromatography on silica gel (DCM: AcOEt 10:1 to 3:1) gave **2** as a yellow solid, 1.89 g, 98%. ^{1}H NMR ([D6]dimethyl sulfoxide): 10.94 (s, 2H), 10.12 (s, 2H), 8.92 (m, 2H), 8.91 (m, 1H), 7.86 (d, *J* = 4.7 Hz, 3 H), 7.79 (m, 2H), 2.39 (t, *J* = 7.3 Hz, 2 H), 1.61 (sx, *J* = 7.4 Hz, 2 H), 0.91 (t, *J* = 7.4 Hz, 3 H); ^{13}C NMR ([D6]dimethyl sulfoxide): δ= 172.1 (2C), 163.5 (2C), 150.6 (2C), 149.8 (2C), 147.7 (2C), 140.1 (2C), 135.8 (2C), 133.6, 125.7 (2C), 110.6, 110.3 (2C), 38.0 (2C), 18.4 (2C), 13.6 (2C) ppm. This data is in accordance with *J. Am. Chem. Soc.*, 1990, **26**, 9589.

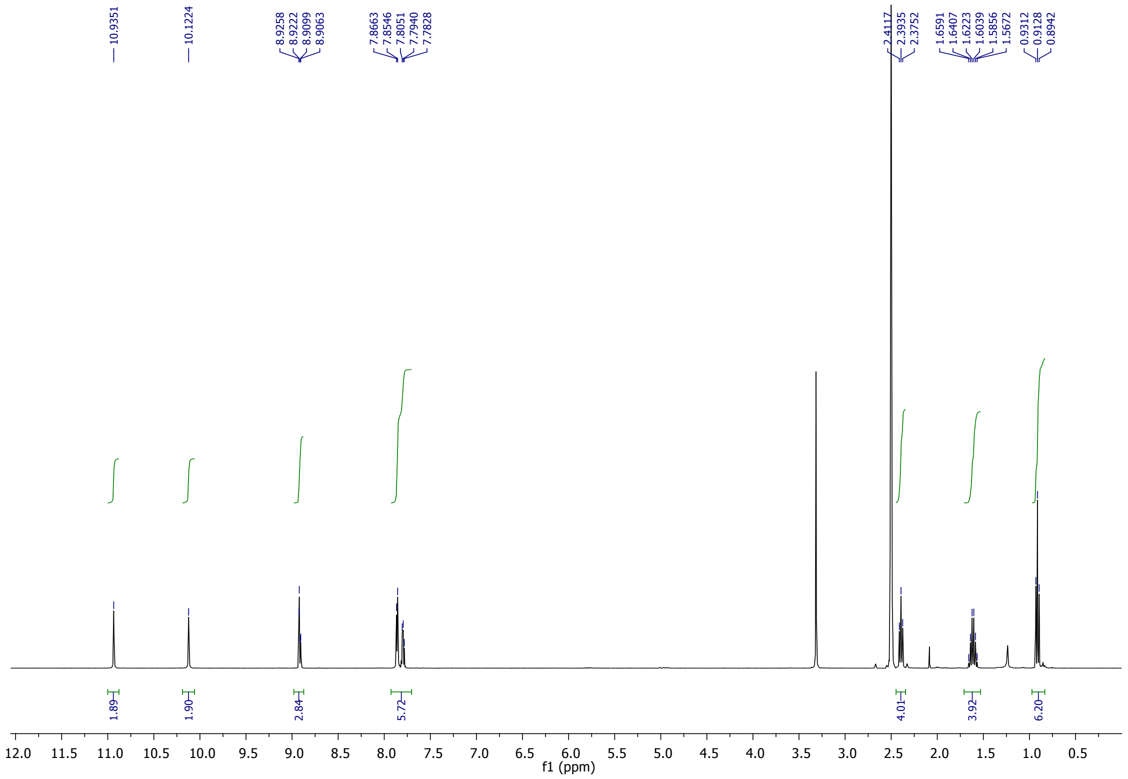

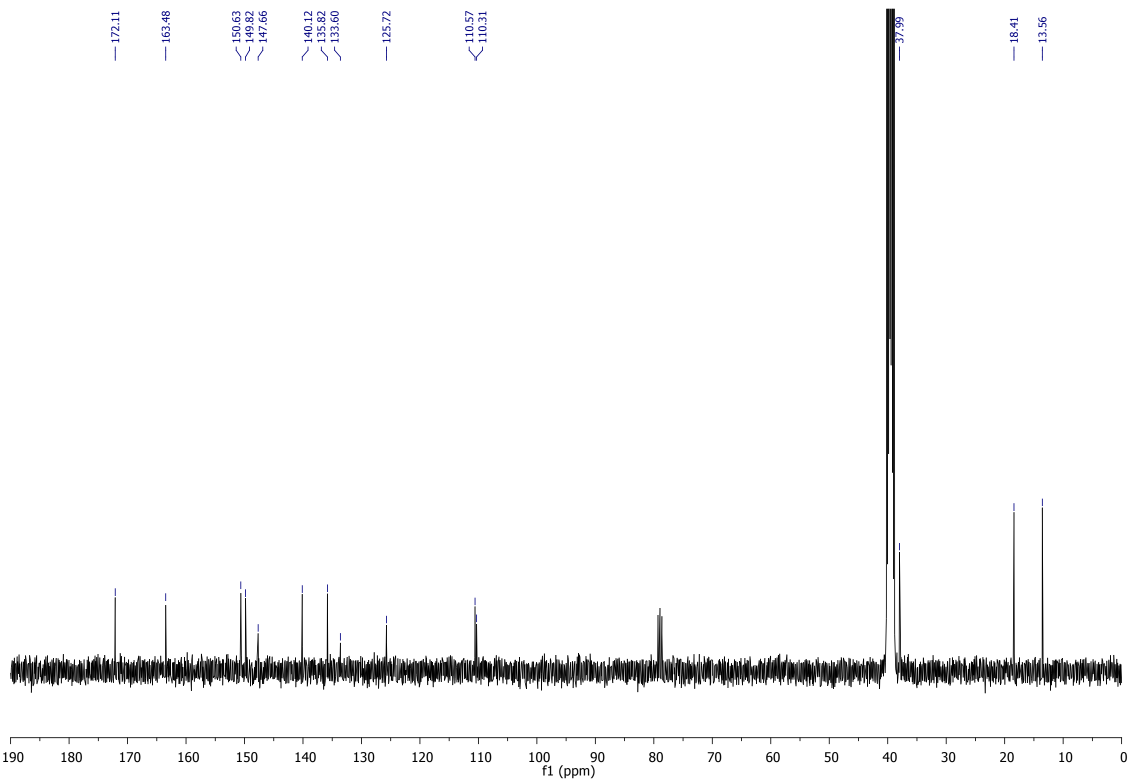

Compound 3. To a solution of **2** (0.4 g, 0.75 mmol) in dry THF (34 mL) and MeOH (34 mL) in a round-botton flask under nitrogen atmosphere, 10% Pd-C (0.4 g) was added and the reaction mixture was heated to 70 ºC. Then hydrazine (1.1g, 20.4 mmol) was added to the reaction mixture, which was heated under reflux for 4h. After cooling the reaction mixture to room temperature, Pd-C was removed by filtering it twice through celite, and the filtrate was evaporated under vacuum to give **3** as a white solid, 0.3 g, 79%. ^{1}H NMR ([D6]dimethyl sulfoxide): 10.18 (s, 2H), 7.80 (m, 6H), 7.70 (m, 1H), 7.31 (d, *J* = 1.4 Hz, 2 H), 5.65 (s, 2H), 2.39 (t, *J* = 7.3 Hz, 2 H), 1.60 (sx, *J* = 7.4 Hz, 2 H), 0.91 (t, *J* = 7.4 Hz, 3 H); ^{13}C NMR ([D6]dimethyl sulfoxide): δ= 172.0 (2C), 165.7 (2C), 150.5 (2C), 150.2 (2C), 149.3 (2C), 140.0 (2C), 138.1, 134.9 (2C), 116.3 (2C), 110.6 (2C), 109.8, 38.0 (2C), 18.4 (2C), 13.6 (2C) ppm. MS m/z: calculated for $C_{26}H_{29}N_7O_4$ $[M+H]^+$ 503.6 found FAB 504.3.

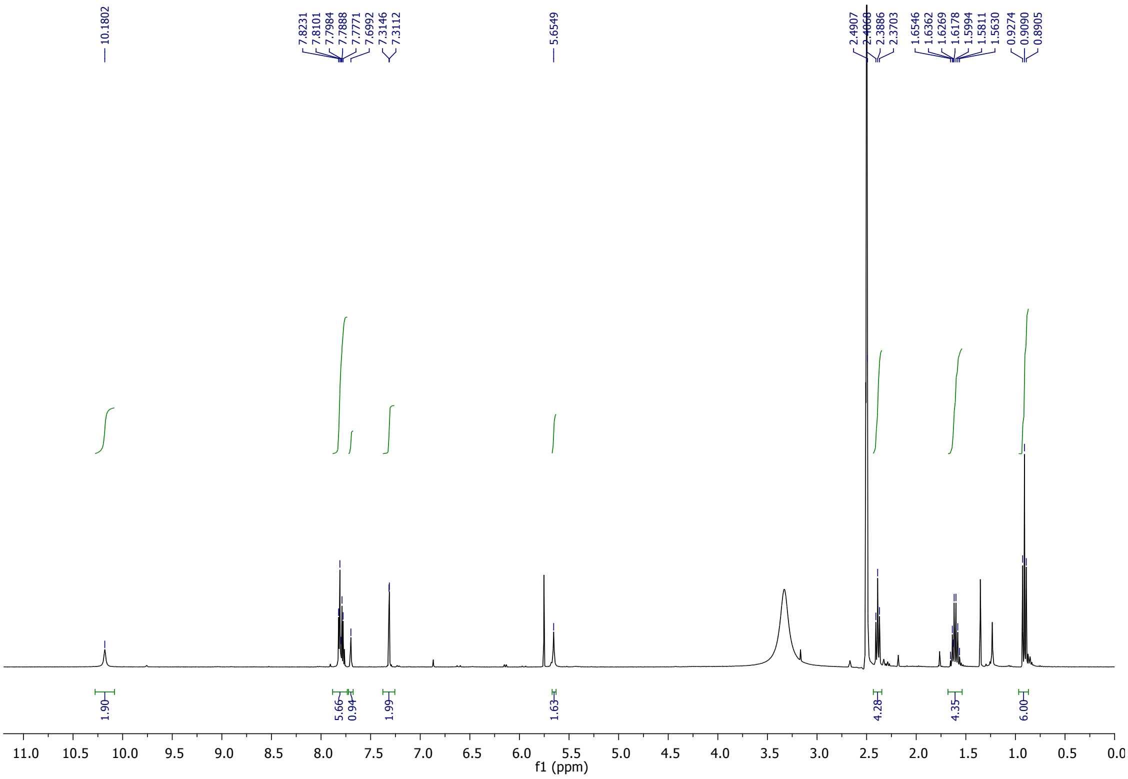

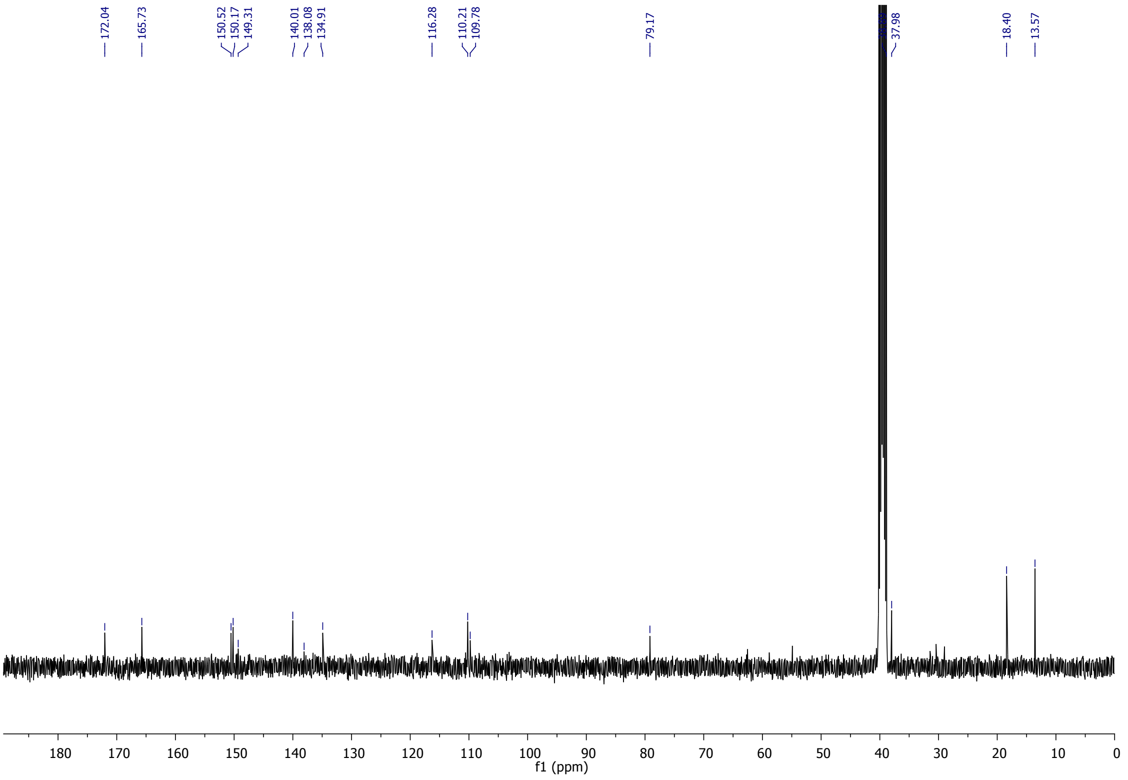

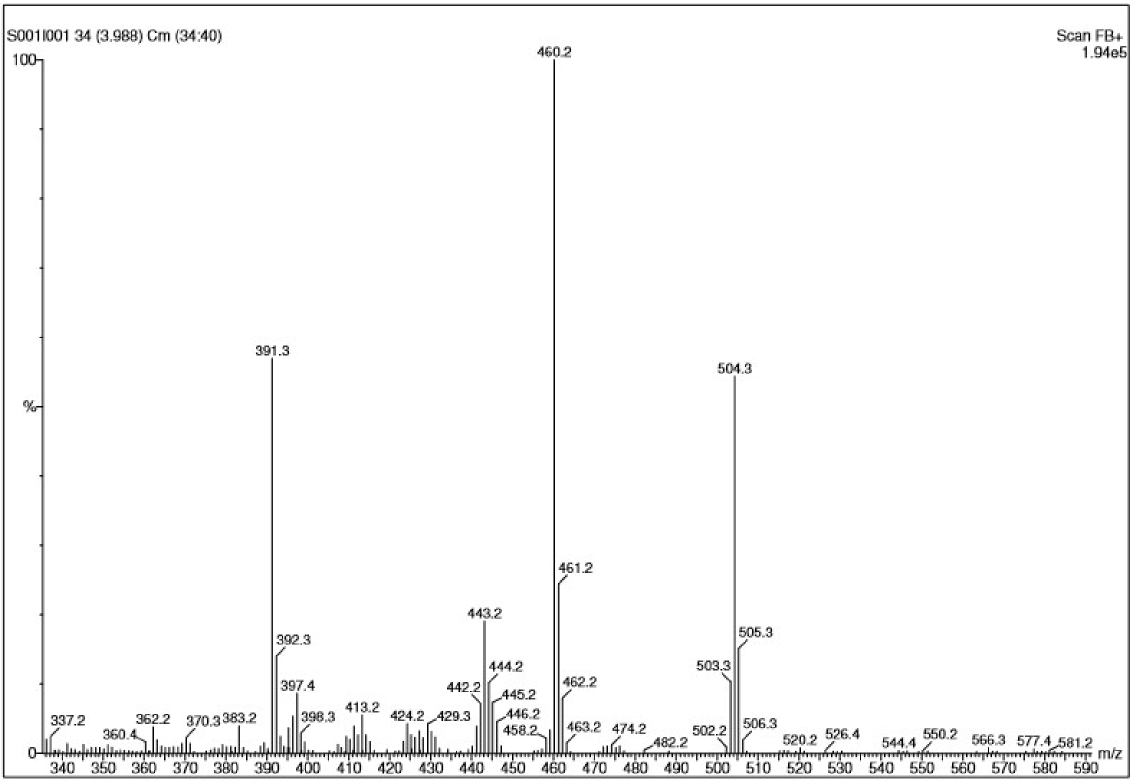
S001I001 34 (3.988) Cm (34:40)
Scan FB+
1.94e5
100
%
0
460.2
391.3
504.3
461.2
443.2
505.3
392.3
503.3
444.2
442.2
462.2
397.4
445.2
413.2
337.2
362.2
370.3
383.2
398.3
424.2
429.3
446.2
458.2
463.2
474.2
360.4
482.2
502.2
506.3
520.2
526.4
544.4
550.2
566.3
577.4
581.2
m/z
340 350 360 370 380 390 400 410 420 430 440 450 460 470 480 490 500 510 520 530 540 550 560 570 580 590

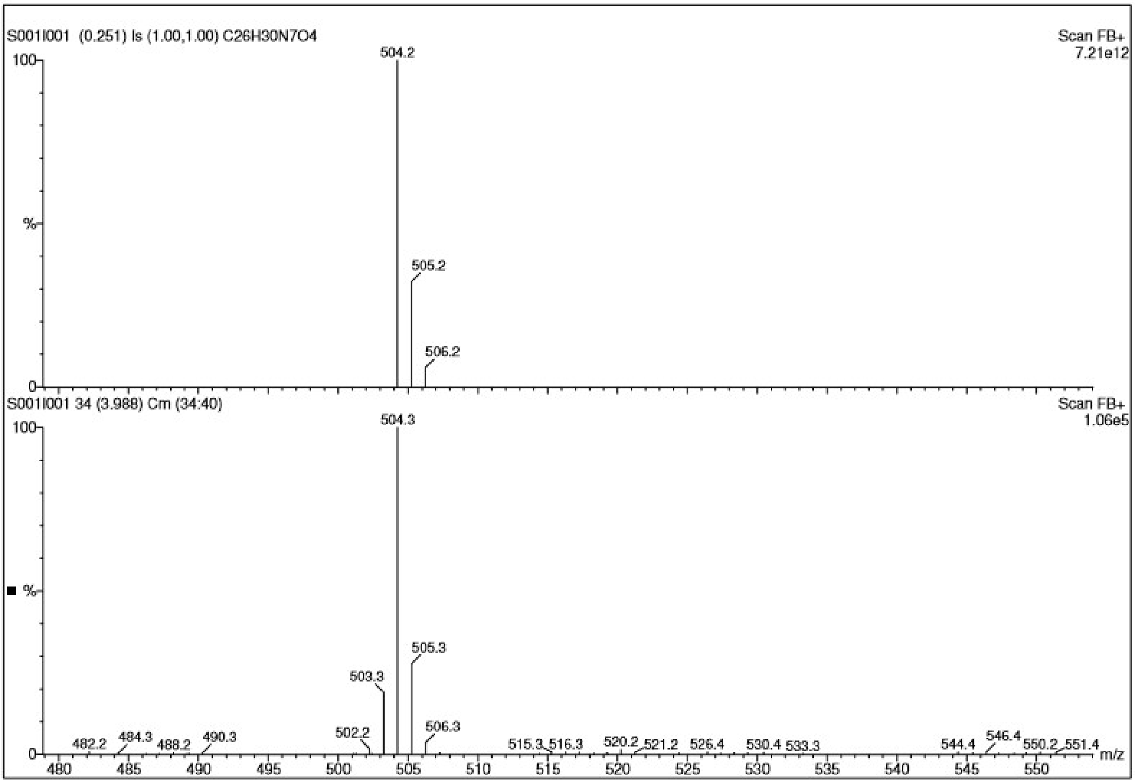
S001I001 (0.251) Is (1.00,1.00) C26H30N7O4
Scan FB+
7.21e12
100
%
0
504.2
505.2
506.2
S001I001 34 (3.988) Cm (34:40)
Scan FB+
1.06e5
100
%
0
504.3
505.3
503.3
506.3
502.2
482.2
484.3
488.2
490.3
515.3
516.3
520.2
521.2
526.4
530.4
533.3
544.4
546.4
550.2
551.4
m/z
480 485 490 495 500 505 510 515 520 525 530 535 540 545 550

Compound 4. 4-pentynoic acid (16 mg, 0.16 mmol) was dissolved in DCM (3.5 mL) and the solution was cooled to 0ºC. EDCI.HCl (50 mg, 0.26 mmol) and DMAP (31 mg, 0.26 mmol) were added. The reaction mixture was allowed to stir at room temperature for 30 minutes. A solution of **3** (75 mg, 0.15 mmol) in DCM (2 mL) was added to the activated acid. The reaction mixture was stirred for 24h, concentrated under reduced pressure and then DCM was added. The organic layer was washed once with 1M HCl, once with $NaHCO_3$ (sat., aq.), then concentrated under reduced pressure. The crude material was purified by column chromatography (eluent: DCM: MeOH 25:1) to furnish product **4** as white solid, 50mg, 57%. ^{1}H NMR ([D6]dimethyl sulfoxide): 10.40 (s, 1H), 10.36 (s, 2H), 10.08 (s, 2H), 8.36 (d, *J* = 1.3 Hz, 2H), 8.22 (m, 1H), 7.81 (m, 6 H), 2.82 (t, *J* = 2.6 Hz, 1 H), 2.58 (m, 2H), 2.38 (t, *J* = 7.3 Hz, 4 H), 1.61 (sx, *J* = 7.4 Hz, 4 H), 0.91 (t, *J* = 7.4 Hz, 6 H); ^{13}C NMR ([D6]dimethyl sulfoxide): δ= 172.1 (2C), 169.9, 165.1 (2C), 150.6 (2C), 150.0 (2C), 140.1 (2C), 139.6 (2C), 134.8 (2C), 121.7, 110.3 (2C), 110.0, 83.5, 71.6, 38.0 (2C), 35.2, 18.4 (2C), 14.0, 13.6 (2C). MS m/z: calculated. for $C_{31}H_{33}N_7O_4$ $[M+H]^+$ 583.6 found MALDI-TOF 584.3.

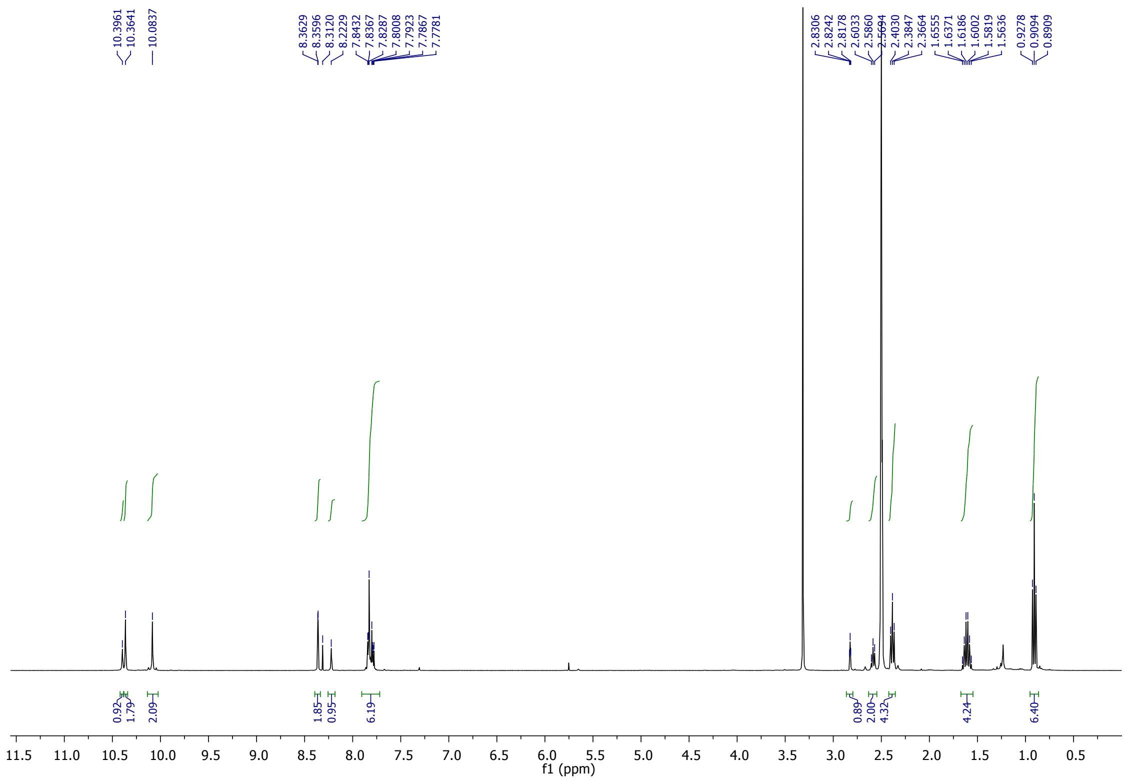

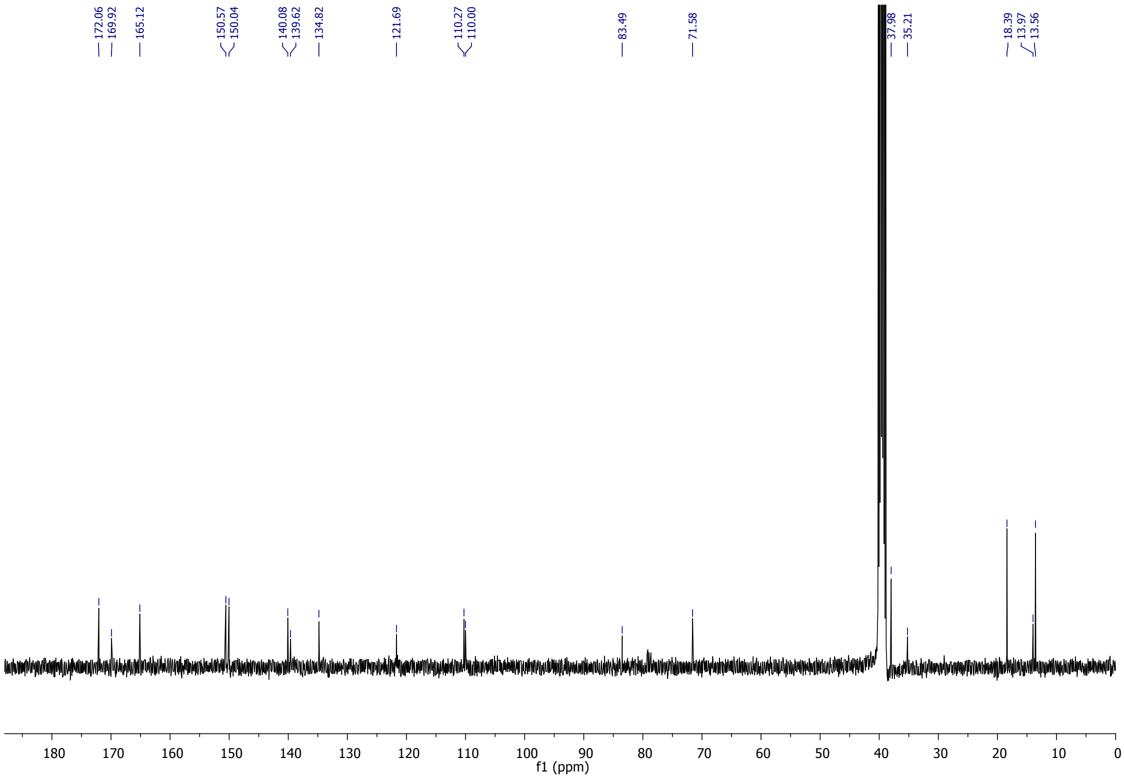

172.06
169.92
165.12
150.57
150.04
140.08
139.62
134.82
121.69
110.27
110.00
83.49
71.58
37.98
35.21
18.39
13.97
13.56
f1 (ppm)

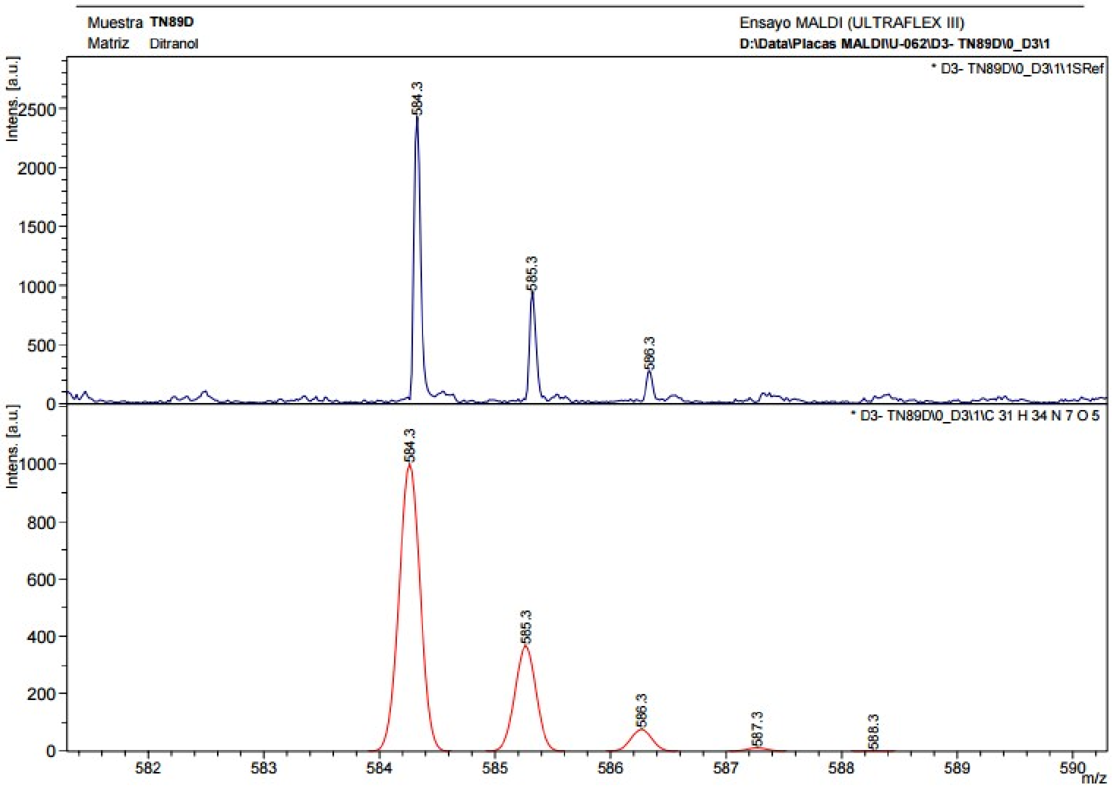

Muestra TN89D
Matriz Ditranol
Ensayo MALDI (ULTRAFLEX III)
D:\Data\Placas MALDI\U-062\D3- TN89D\0_D3\1
* D3- TN89D\0_D3\1\1SRef
Intens. [a.u.]
584.3
585.3
586.3
* D3- TN89D\0_D3\1\C 31 H 34 N 7 O 5
584.3
585.3
586.3
587.3
588.3
m/z

Compound 5. 4-Azidobenzoic acid (447 mg, 2.74 mmol) was solved in dry DMF (6.6 mL) under argon atmosphere; then HOBt (339 mg, 2.51 mmol) and DCC (518 mg, 2.51 mmol) were added. After 5 minutes stirring at room temperature *L*-Threoninol (240 mg, 2.28 mmol) was added. The reaction was stirring overnight. The white solid was filtered off and solvent was removed under reduced pressure. The product was purified by column chromatography (gradient elution DCM: MeOH 50:1 to 20:1) to give **5** as a yellow solid, 430 mg, 75%. ^{1}H NMR ([D6]dimethyl sulfoxide): 7.92 (d, *J* = 6.7 Hz, 2 H), 7.74 (br d, *J* = 8.4 Hz, 2 H), 7.19 (d, *J* = 7.3 Hz, 2H), 4.59 (m, 2H), 3.89 (m, 2H), 3.58 (m, 1H), 3.47 (m, 1H), 1.05 (d, *J* = 6.3 Hz, 3 H); ^{13}C NMR ([D6]dimethyl sulfoxide): δ= 165.6, 142.1, 131.4, 129.3, 118.8, 64.9, 60.4, 56.7, 20.2 ppm. MS m/z: calculated. for $C_{11}H_{14}N_4O_3$ $[M+H]^+$ 250.2 found FAB 251.0.

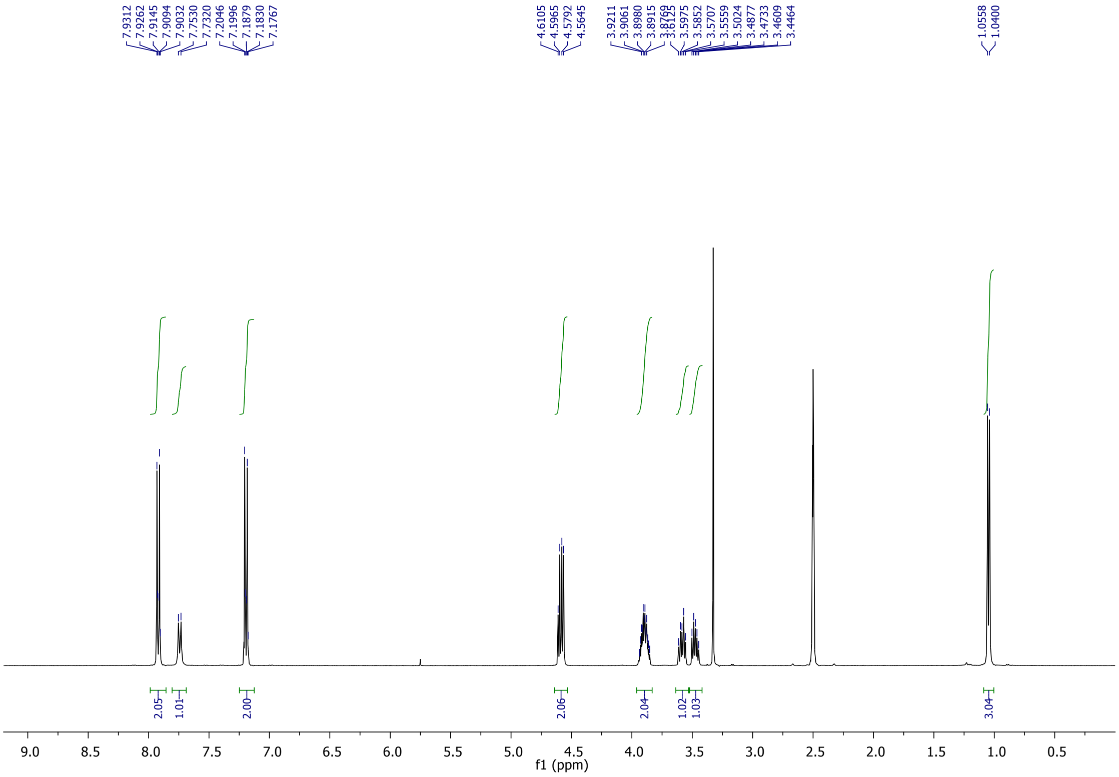

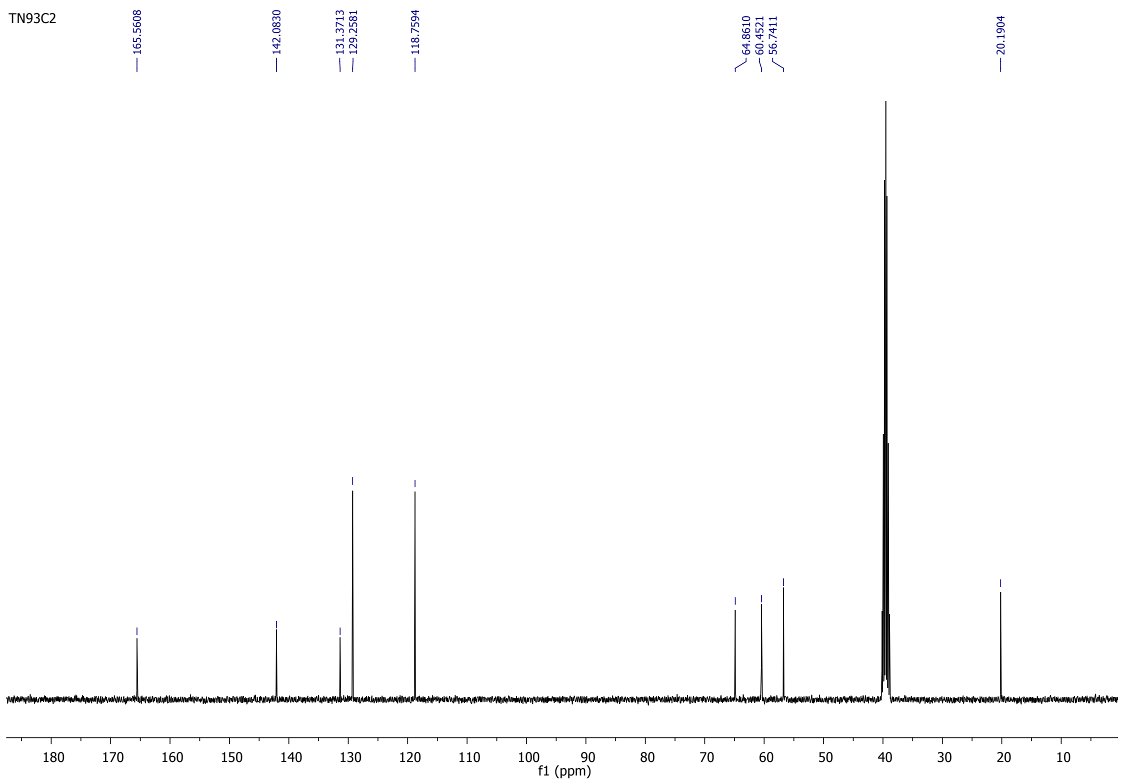

TN93C2
165.5608
142.0830
131.3713
129.2581
118.7594
64.8610
60.4521
56.7411
20.1904
f1 (ppm)

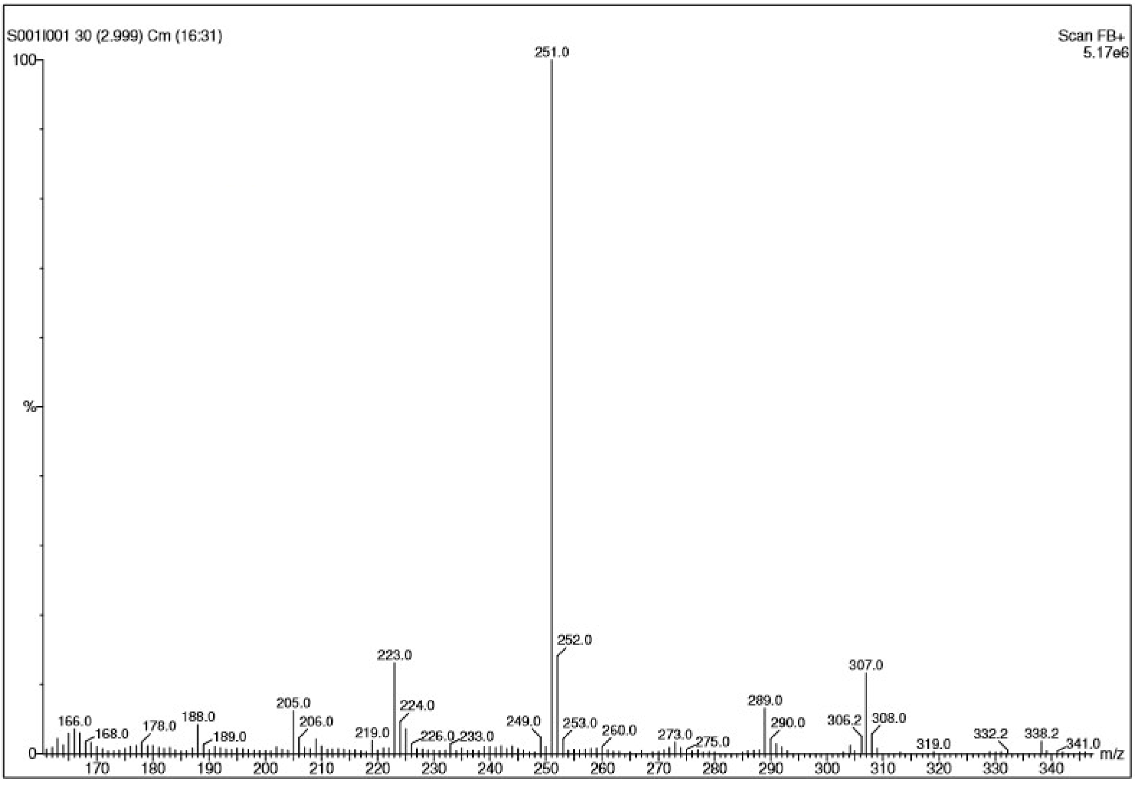

S001I001 30 (2.999) Cm (16:31)
Scan FB+
5.17e6
251.0
252.0
223.0
307.0
289.0
205.0
224.0
m/z

Compound 6. Compound **5** (192 mg, 0.77 mmol) was solved in Pyridine under argon atmosphere. Afterwards DIPEA (149 mg, 1.16 mmol), DMAP (150 mg, 1.16 mmol) and 4,4′-Dimethoxytrityl chloride (313 mg,0.92 mmol) were added at 0ºC. The reaction was stirred overnight. Pyridine was removed under reduced pressure and the desired product was purified by flash column chromatography using neutralized SiO_2 (neutralized with trimethylamine), using gradient elution Hexane: AcOEt 2 : 1 to 1:1 to give **6** as a colorless solid, 350 mg, 82%.^{1}H NMR ([D6]dimethyl sulfoxide): 8.02 (d, *J* = 8.7 Hz, 2 H), 7.96 (d, *J* = 8.6 Hz, 2 H), 7.38 (br d, *J* = 8.8 Hz, 2 H), 7.23 (m, 8H), 6.83 (dd, *J* = 8.9, 3.4 Hz, 2 H), 4.55 (d, *J* = 6.2 Hz, 1H), 4.10 (m, 2H), 4.01 (m, 2H), 3.72 (s, 6H), 3.21 (dd, *J* = 9, 5.4 Hz, 1H), 2.96 (dd, *J* = 9, 6.2 Hz, 1H), 1.00 (d, *J* = 6.3 Hz, 3 H); ^{13}C NMR ([D6]dimethyl sulfoxide): δ= 165.6, 158.0 (2C), 145.1, 142.2, 135.9 (2C), 135.7 (2C), 131.3 (2C), 129.7 (2C), 129.3 (2C), 127.7 (2C), 127.7 (2C), 126.5, 124.5, 118.8 (2C), 113.07 (2C), 85.1, 65.2, 63.0, 55.2, 55.0 (2C), 20.3 ppm. MS m/z: calculated. for $C_{32}H_{32}N_4O_5$ $[M]^+$ 552.2 found FAB 552.2.

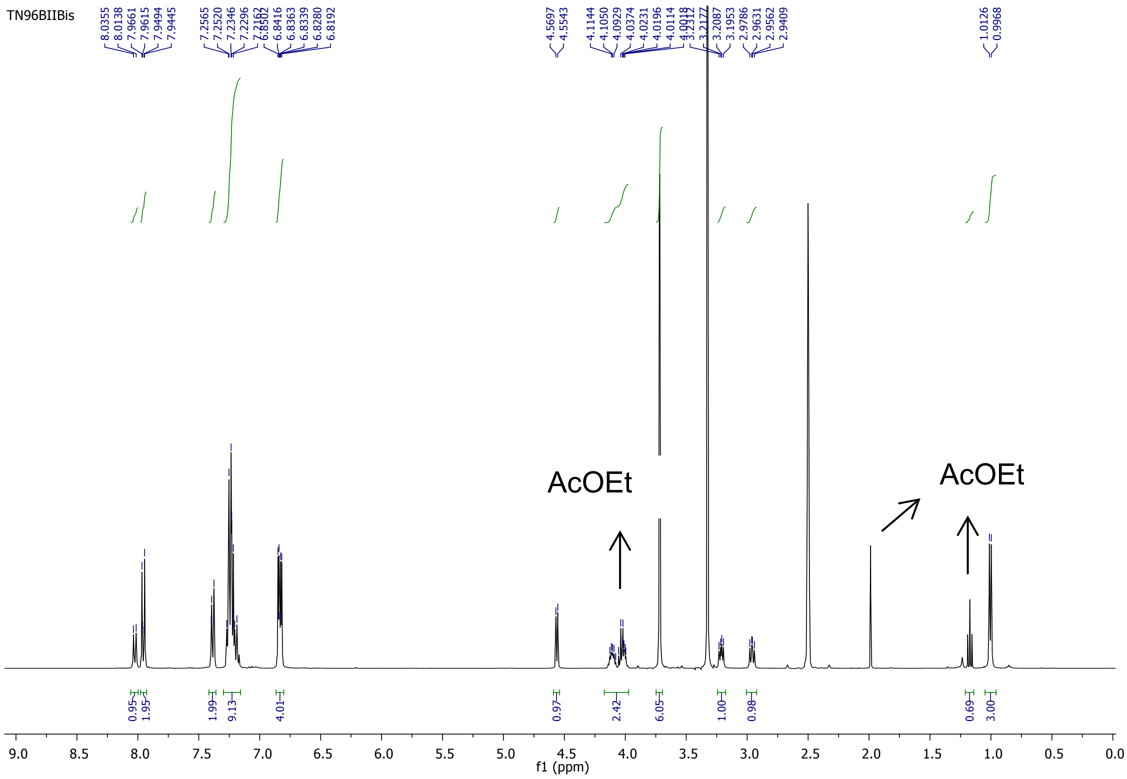

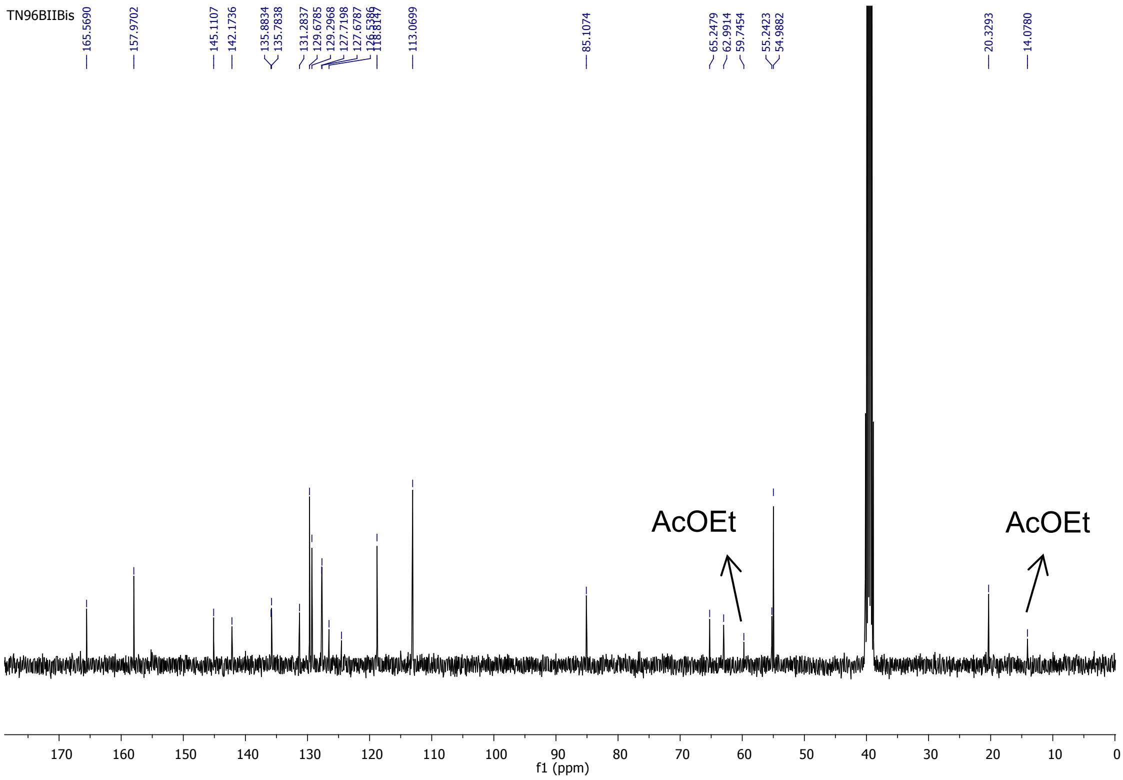
TN96BIIBis
165.5690
157.9702
145.1107
142.1736
135.8834
135.7838
131.2837
129.6785
129.2968
127.7198
127.6787
85.1074
65.2479
62.9914
59.7454
55.2423
54.9882
113.0699
20.3293
14.0780
AcOEt
AcOEt
f1 (ppm)

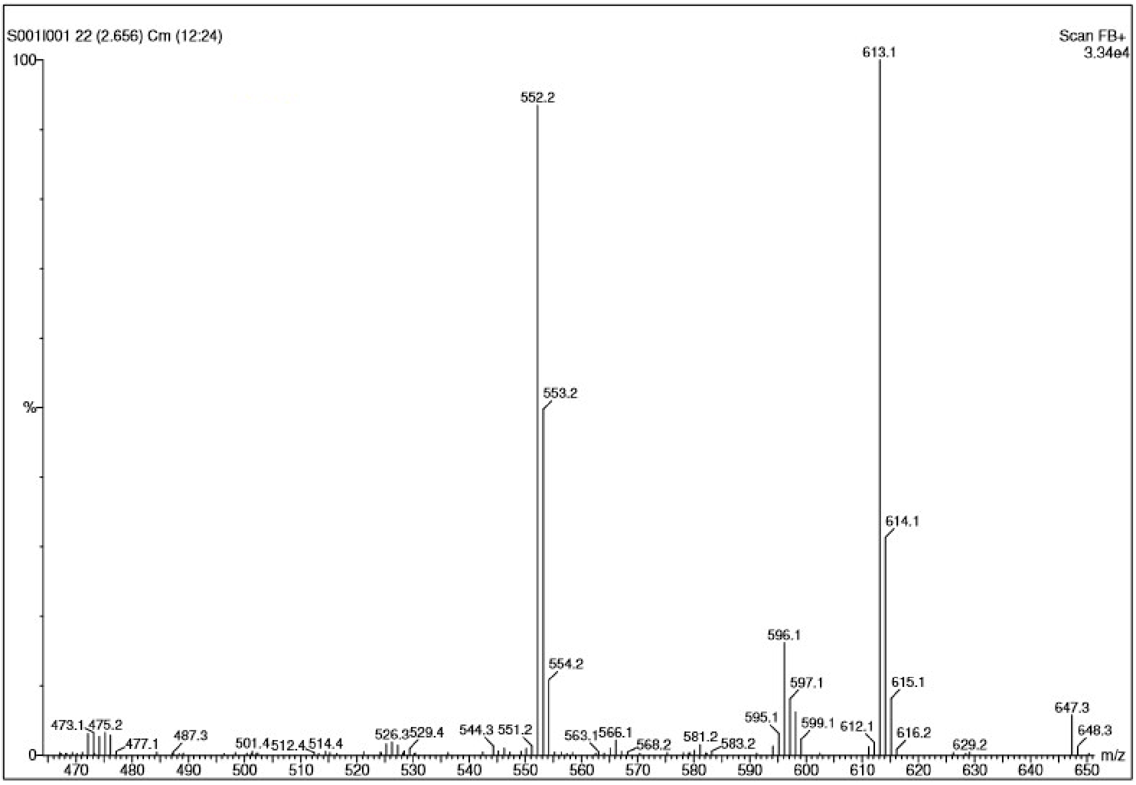
S001I001 22 (2.656) Cm (12:24)
Scan FB+
3.34e4
613.1
552.2
553.2
614.1
596.1
554.2
597.1
615.1
647.3
595.1
648.3
473.1
475.2
487.3
599.1
612.1
616.2
%
m/z

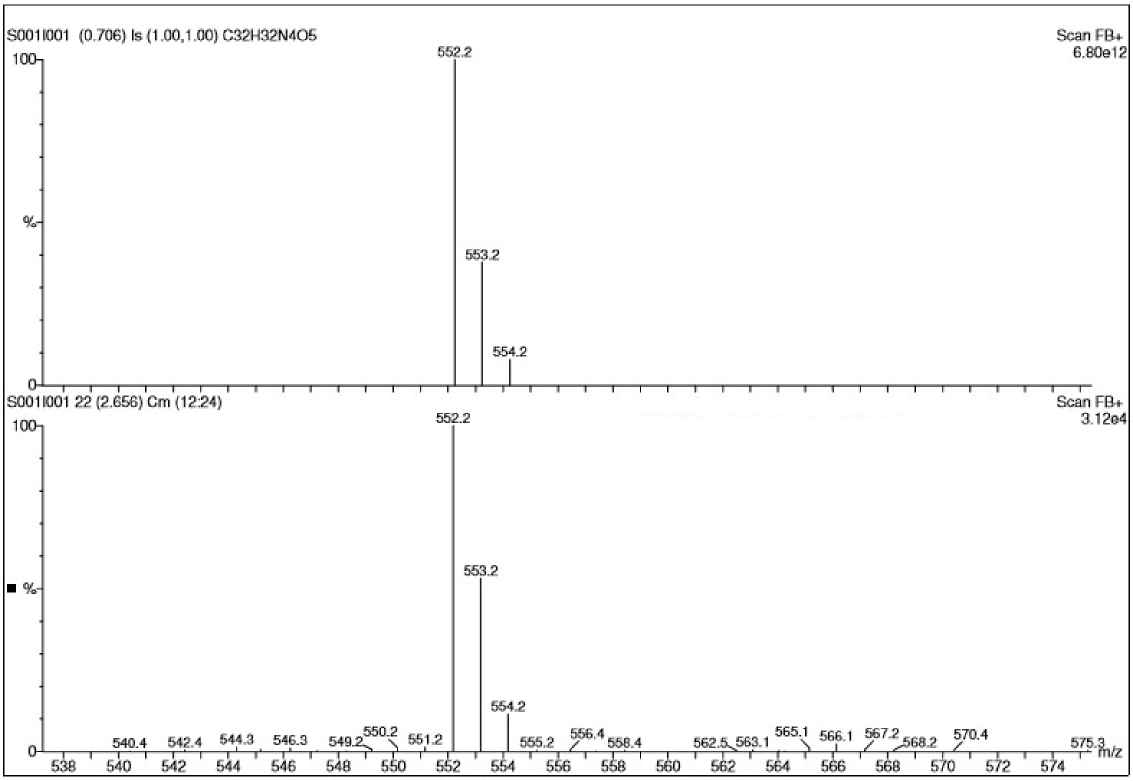

Compound 7. Compound **5** (20 mg, 0.034 mmol) and **6** (19 mg, 0.034 mmol) were dissolved in anhydrous DMF (1 mL); then DIPEA was added (4.4 mg, 0.034 mmol), followed by the addition of catalytic amount of CuI. The reaction was stirred for 3h. Once it was completed (TLC), the solvent was removed under reduced pressure and the product was purified by flash column chromatography using neutralized SiO_2 (neutralized with trimethylamine), using gradient elution DCM : MeOH 25 : 1 to 15:1 to give **7** as a colorless oil, quantitative yield. ^{1}H NMR ([D6]dimethyl sulfoxide): 10.35 (br d, *J* = 3.2 Hz, 2 H), 10.07 (br d, *J* = 4.3 Hz, 2 H), 8.36 (m, 2H), 8.21 (s, 1H), 8.07 (d, *J* = 8.6 Hz, 2 H), 7.80 (m, 7H), 7.23 (m, 4H), 7.07 (d, *J* = 8.7 Hz, 2 H), 6.83 (br d, *J* = 8.7 Hz, 2 H), 6.20 (s, 1H), 4.60 (m, 2H), 3.92 (m, 2H), 3.72 (S, 6H), 3.59 (m, 2H), 2.85 (m, 2H), 2.38 (t, *J* = 7.3 Hz, 1H), 1.60 (sx, *J* = 7.3 Hz, 1H),1.06 (d, *J* = 6.3 Hz, 3 H). 0.91 (t, *J* = 7.3 Hz, 3 H). MS m/z: calculated for $C_{63}H_{65}N_{11}O_{10}$ [M-DMTr+H]$^+$ 833.9 found MALDI-TOF 834.4.

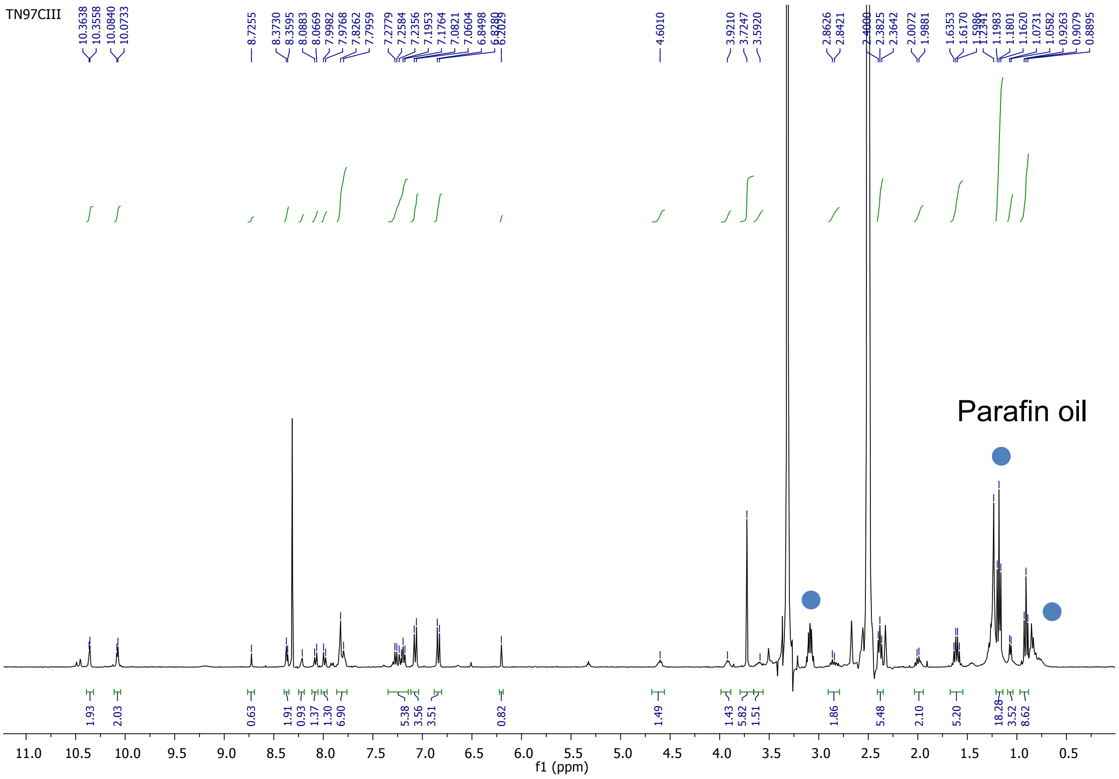
TN97CIII
Parafin oil
f1 (ppm)

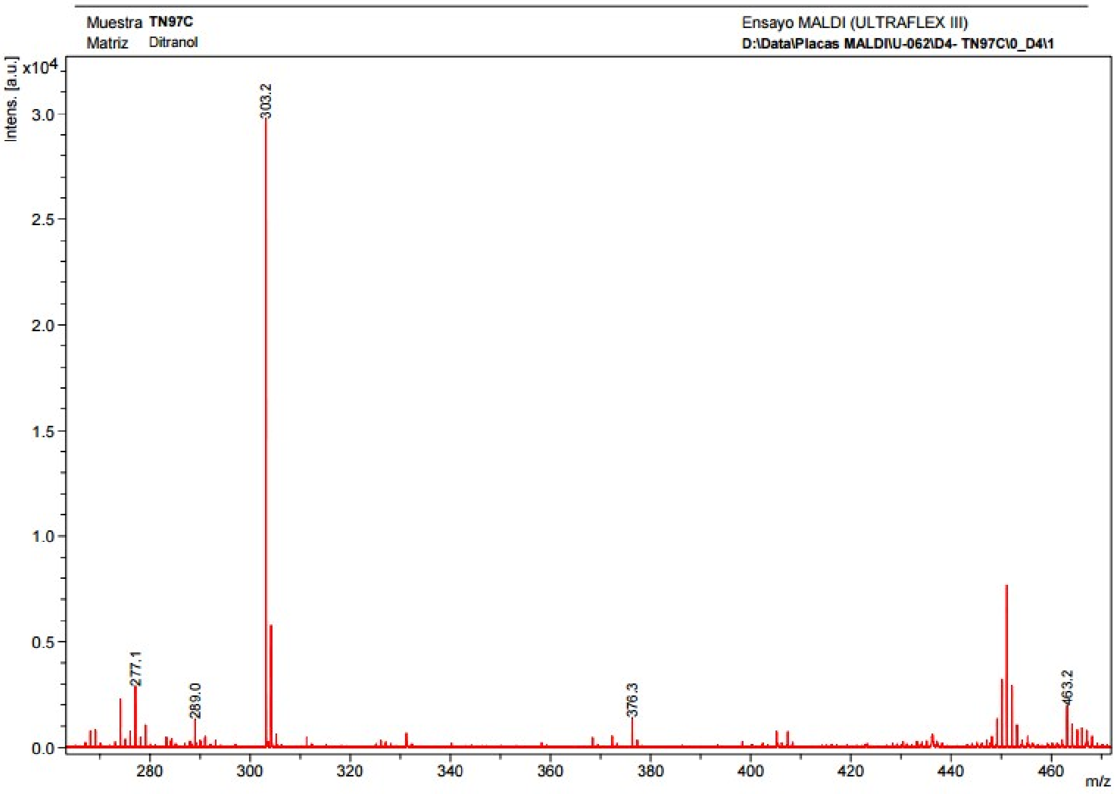
Muestra TN97C
Matriz Ditranol
Ensayo MALDI (ULTRAFLEX III)
D:\Data\Placas MALDI\U-062\D4- TN97C\0_D4\1
Intens. [a.u.]
303.2
277.1
289.0
376.3
463.2
m/z

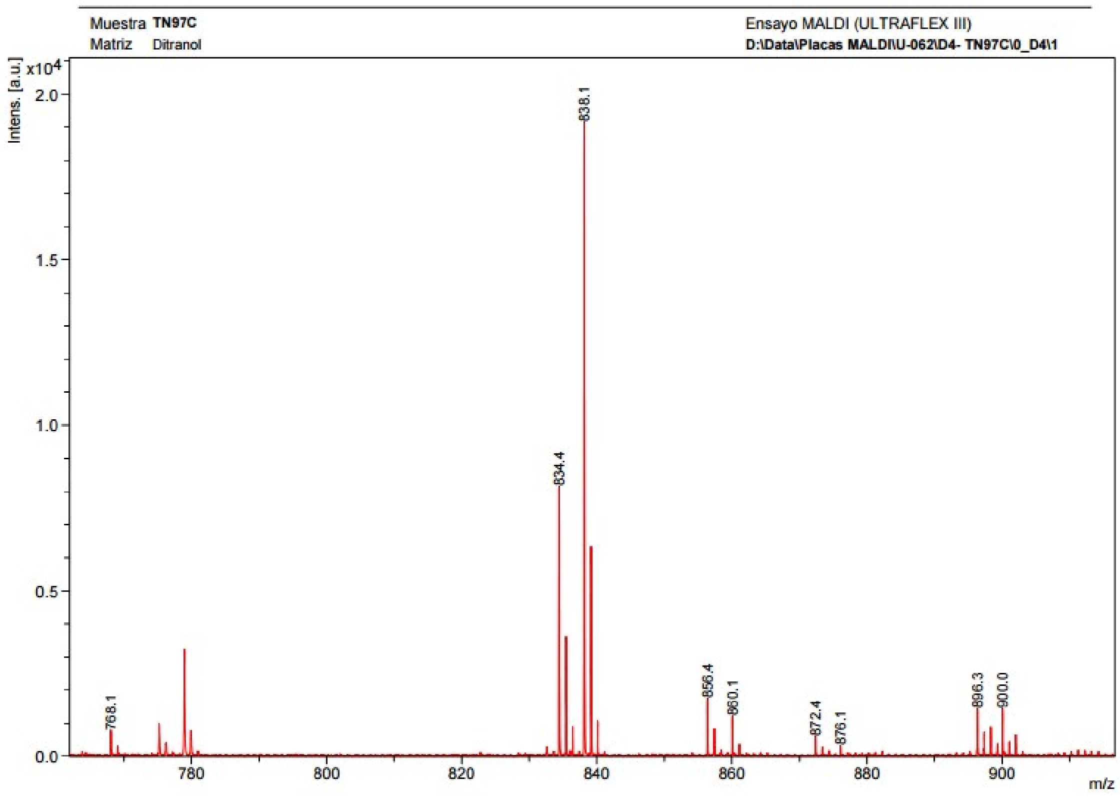

2. Preparation of modified solid supports

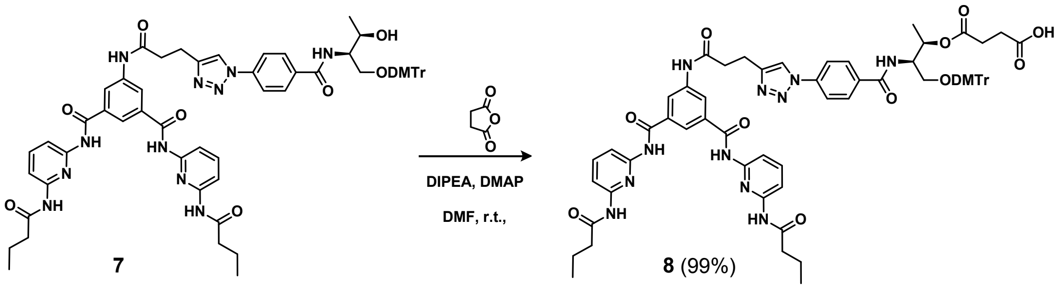

7 **8** (99%)

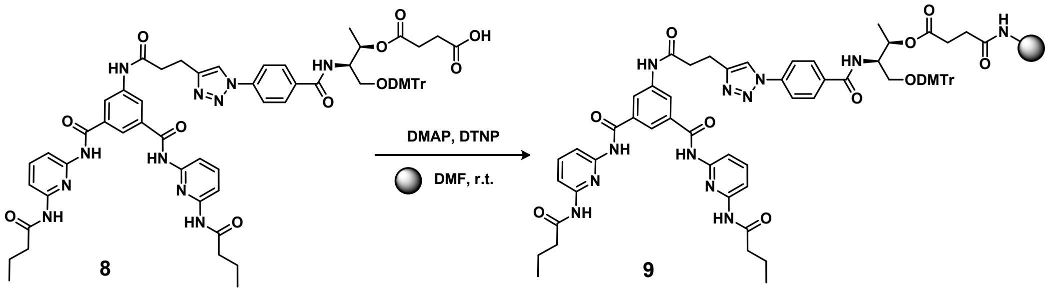

8 **9**

Compound 8. To a solution of **7** (20 mg, 002 mmol) in DMF (0.5 mL), succinic anhydride (3 mg, 0.03 mmol) was then added, followed by the addition of catalytic amount of DMAP and DIPEA (4 mg, 0.03 mmol). The reaction mixture was stirred overnight. The solvent was removed under reduced pressure. The crude material was dissolved in EtOAc and the organic layer was washed with brine (sat. aq.). The product **8** was used directly in the next reaction step without further purification.

Compound 9. 250 mg of commercial solid support (LCAA-CPG-500 Å) were introduced in a specific column. On a round botton flask, compound **8** (20 mg, 0.016 mmol) was dissolved in dry DMF (0.8 mL), then DCC (5 mg, 0.024 mmol) was added, followed by the addition of HOBt (3 mg, 0.022 mmol); the reaction mixture was introduced quickly in the column with the solid support (ensuring that the column was closed). The column was covered and it was swirling with a vortex every 30 min, repeating it for 5h. Then, the excess of liquid was filtered off and the column was washed several times with MeOH and acetonitrile. The modify solid support was treated with 1mL of CAP A (600 μL of Py, 500 μL THF, 400 μL Ac_2O) and 1mL of CAP B (1 mL THF, 400 μL 1-methylimidazol), reacted for 30 min, and filtered again and washed successively with 10 mL portions of MeOH and MeCN and dried in vacuo. Loading was determined by treating a portion (10 mg) of compound **9** with 1 mL of 18% v/v $HClO_4$ in EtOH and measuring the absorbance of DMTr cation at 504 nm (ε= 75 mL cm^{-1} μmol^{-1}). This was found to be 6.7 μmol/g.

3. Cyanuric acid derivate synthesis

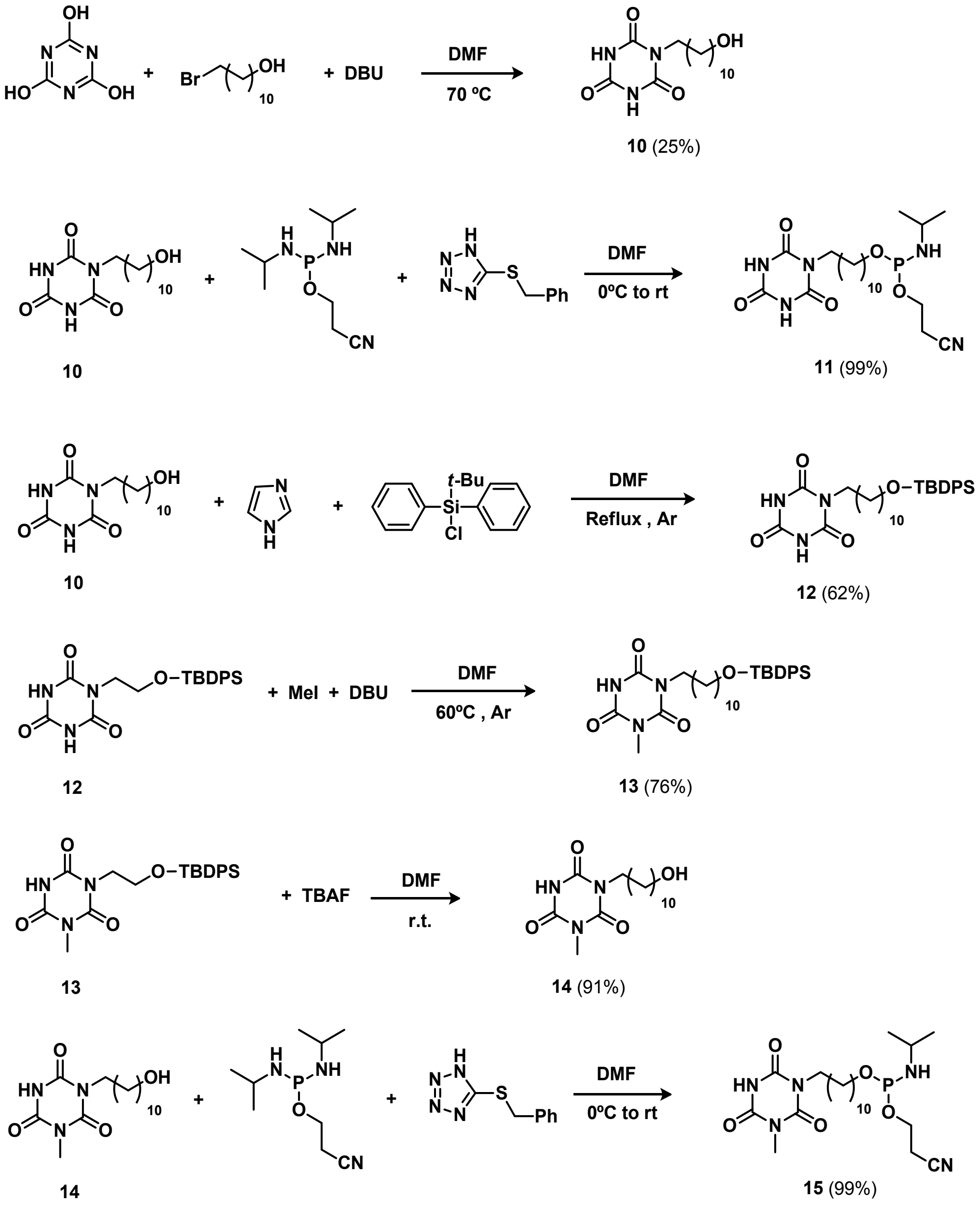

Compound 10. To a solution of cyanuric acid (1.03 g, 7.96 mmol) in DMF (16mL) was added 11-Bromo-1-undecanol (0.5 g, 2 mmol) and 1,8-diazabicycloundec-7-ene (0.32 g, 2 mmol). The reaction mixture was heated under 70ºC overnight, poured into water, and extracted with ethyl acetate. The organic layer was washed with water to eliminate the excess of cyanuric acid, dried with $MgSO_4$ and filtered. The solvent was removed under reduced pressure and the crude material was purified by column chromatography using DCM : MeOH 15:1 as a eluent to give **11** as a white solid, 596 mg, 25%.[1]H

NMR ([D6]dimethyl sulfoxide): 11.34 (br s, 2H), 4.32 (br s, 1H), 3.62 (t, *J* = 7.2 Hz, 2 H), 3.37 (m, 2H), 1.51 (m, 2H), 1.40 (m, 2H), 1.25 (s, 14); ^{13}C NMR ([D6]dimethyl sulfoxide): δ= 149.9 (2C), 148.7, 60.7, 40.4, 32.5, 29.1, 28.9 (2C), 28.9, 28.7, 27.3, 26.1, 25.5.ppm. MS m/z: calculated. for $C_{14}H_{25}N_3O_4$ $[M+Na]^+$ 322.4 found MALDI-TOF 322.2.

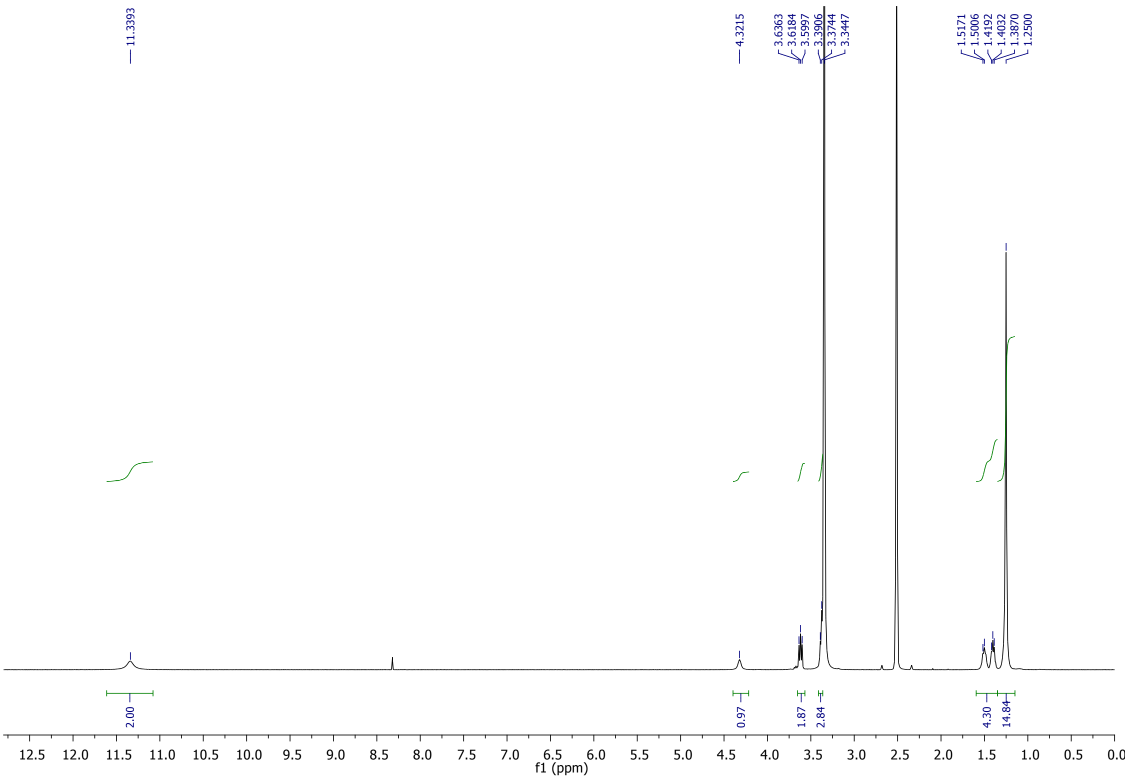

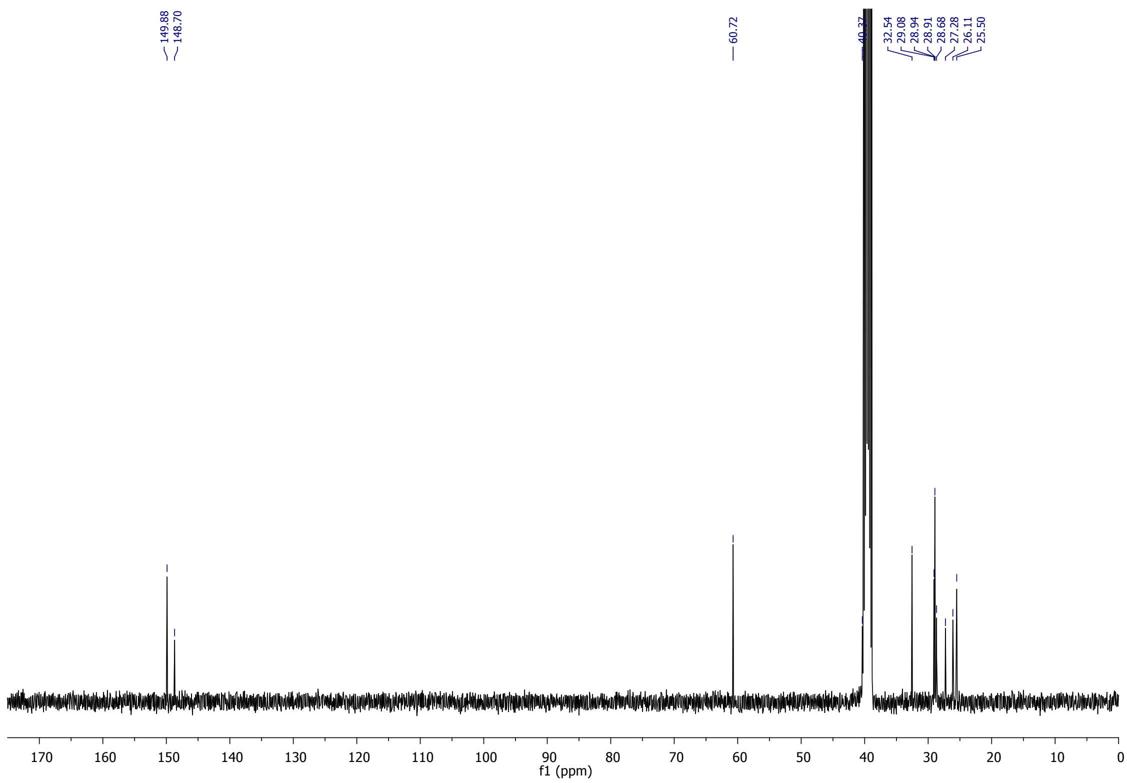
149.88
148.70
60.72
32.54
29.08
28.94
28.91
28.68
27.28
26.11
25.50
170 160 150 140 130 120 110 100 90 80 70 60 50 40 30 20 10 0
f1 (ppm)

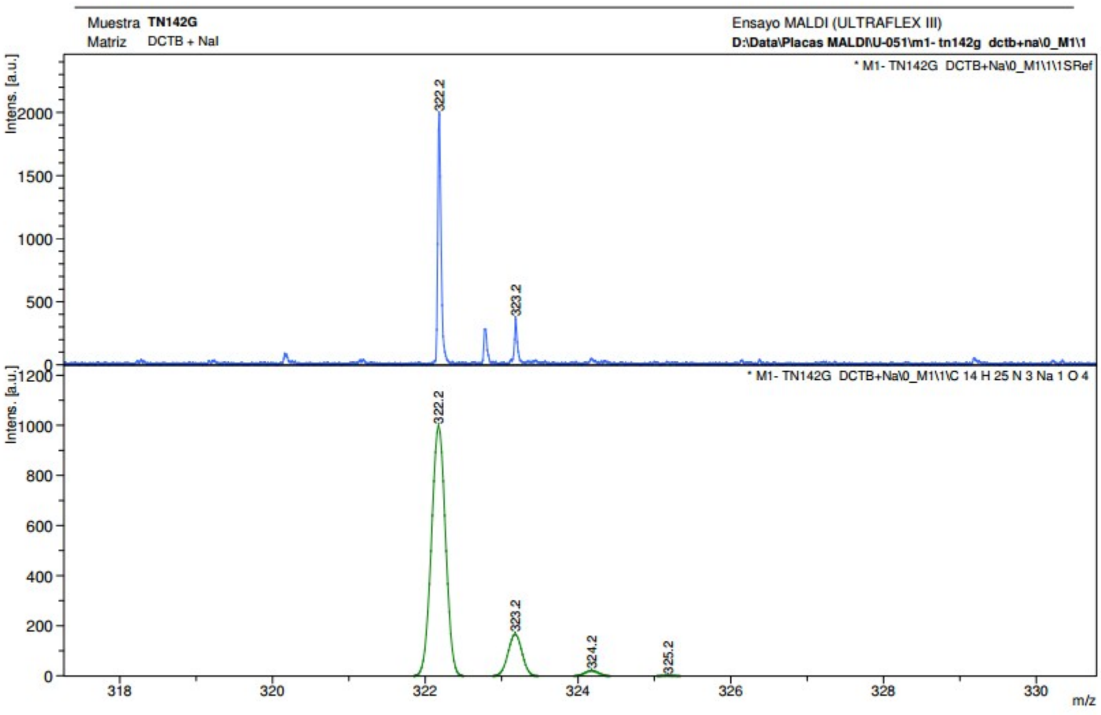
Muestra TN142G
Matriz DCTB + NaI
Ensayo MALDI (ULTRAFLEX III)
D:\Data\Placas MALDI\U-051\m1- tn142g dctb+na\0_M1\1
* M1- TN142G DCTB+Na\0_M1\1\1SRef
Intens. [a.u.]
2000
1500
1000
500
0
322.2
323.2
* M1- TN142G DCTB+Na\0_M1\1\C 14 H 25 N 3 Na 1 O 4
1200
1000
800
600
400
200
322.2
323.2
324.2
325.2
318 320 322 324 326 328 330
m/z

Compound 11. Compound **10** (100 mg, 0.33 mmol) was solved in dry DMF (1 mL) and the solution was cooled to 0ºC. Then, 5-Benzylthio-1-H-tetrazole (76 mg, 0.4 mmol) and the phosphorylating reagent, 2-Cyanoethoxy-bis(N,N-diisopropylamino) phosphine (100 mg, 0.33 mmol), were added. The reaction mixture was allowed to stir at room temperature for 3h under argon atmosphere. When the reaction was completed (followed by TLC), the crude material was using directly, without further purification, in the last step of the automated solid phase synthesis of the corresponding oligonucleotide (oligonucleotide **4** and oligonucleotide **7**), due to compound **11** is a very unstable product, we were not able to isolate it.

Compound 12. Compound **10** (310 mg, 1.11 mmol) was solved in dry DMF (6 mL). Imidazole (300 mg, 4.46 mmol) and *tert*-Butyl(chloro)diphenylsilane (337 mg, 1.23 mmol) were added. The reaction mixture was refluxed under argon atmosphere for 3h. The solvent was removed under reduced pressure and the crude material was purified by column chromatography using a gradient elution DCM : MeOH 100 : 1 to 50 : 1, to give **12** as a white solid, 380mg, 62%. ^{1}H NMR ([D6]dimethyl sulfoxide): 11.37 (br s, 2H), 7.61 (m, 4H), 7.44 (m, 6H), 4.32 (br s, 1H), 3.62 (m, 4H), 1.51 (m, 4H), 1.31 (m, 2H), 1.21 (br s, 12), 0.99 (s, 9H); ^{13}C NMR ([D6]dimethyl sulfoxide): δ= 149.8 (2C), 148.6, 135.0 (4C), 133.4 (2C), 129.8 (2C),127.8 (4C), 63.4, 40.4, 31.9, 28.9, 28.8 (2C), 28.7, 28.6, 27.3, 26.7, 26.1, 25.1, 18.8 (3C) ppm. MS m/z: calculated. for $C_{30}H_{43}N_3O_4Si$ $[M+H]^+$ 538.3 found FAB 538.3.

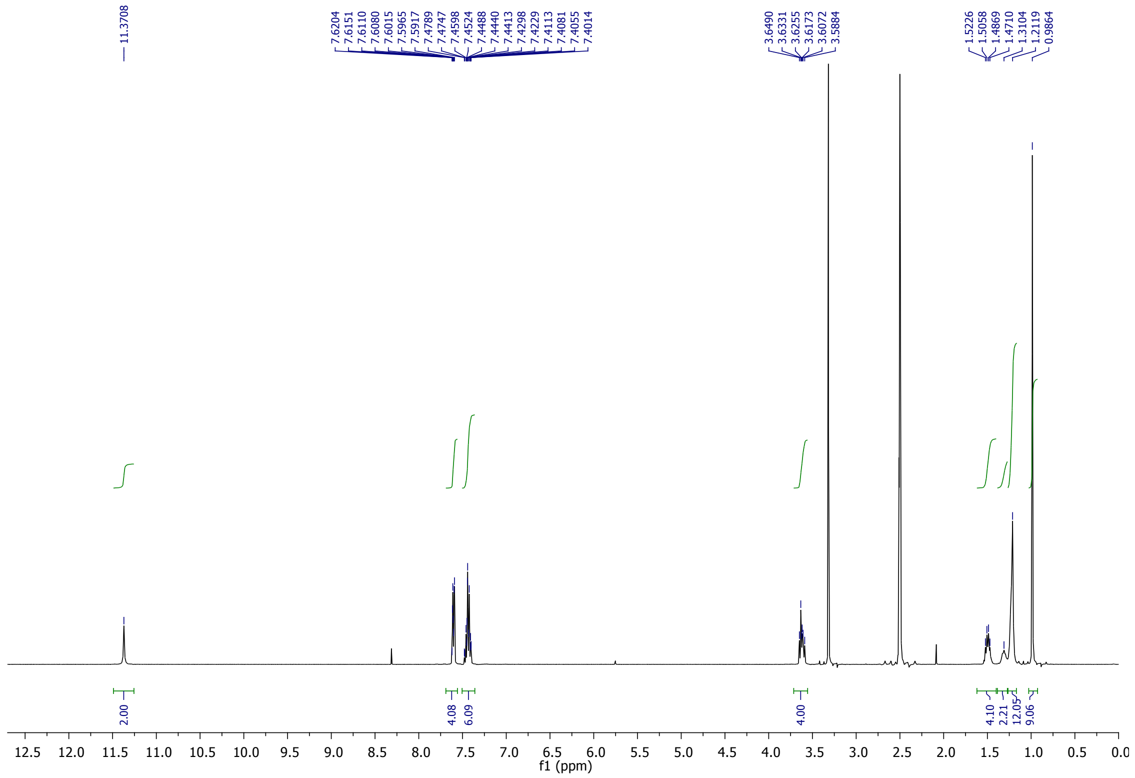

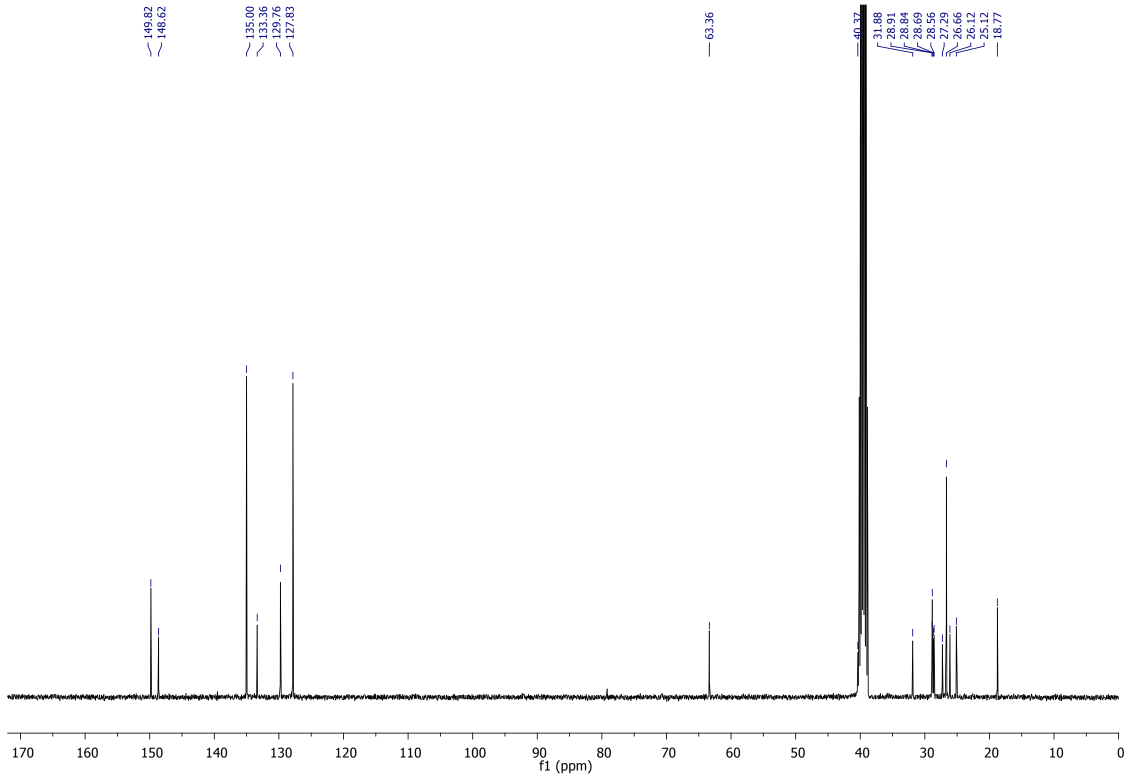

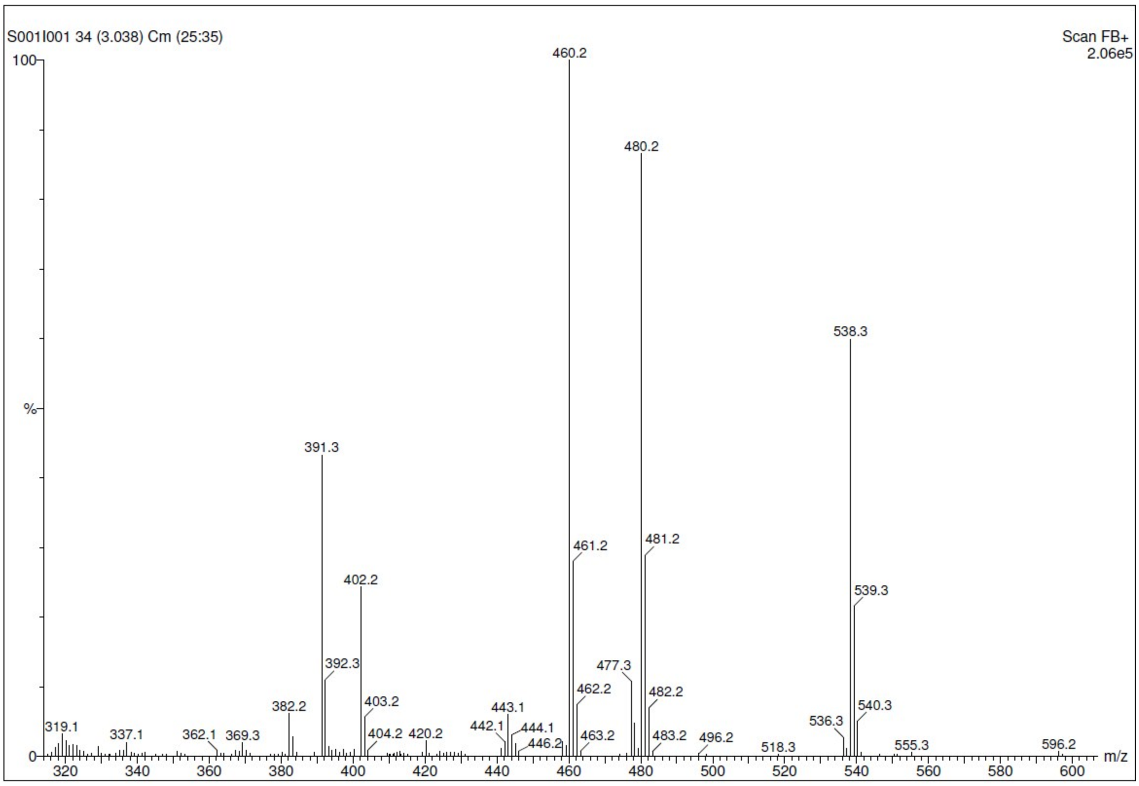
S001I001 34 (3.038) Cm (25:35)
Scan FB+
2.06e5
100
%
0
460.2
480.2
538.3
391.3
461.2
481.2
402.2
539.3
392.3
477.3
462.2
482.2
382.2
403.2
443.1
540.3
536.3
319.1
337.1
362.1
369.3
404.2
420.2
442.1
444.1
446.2
463.2
483.2
496.2
518.3
555.3
596.2
m/z

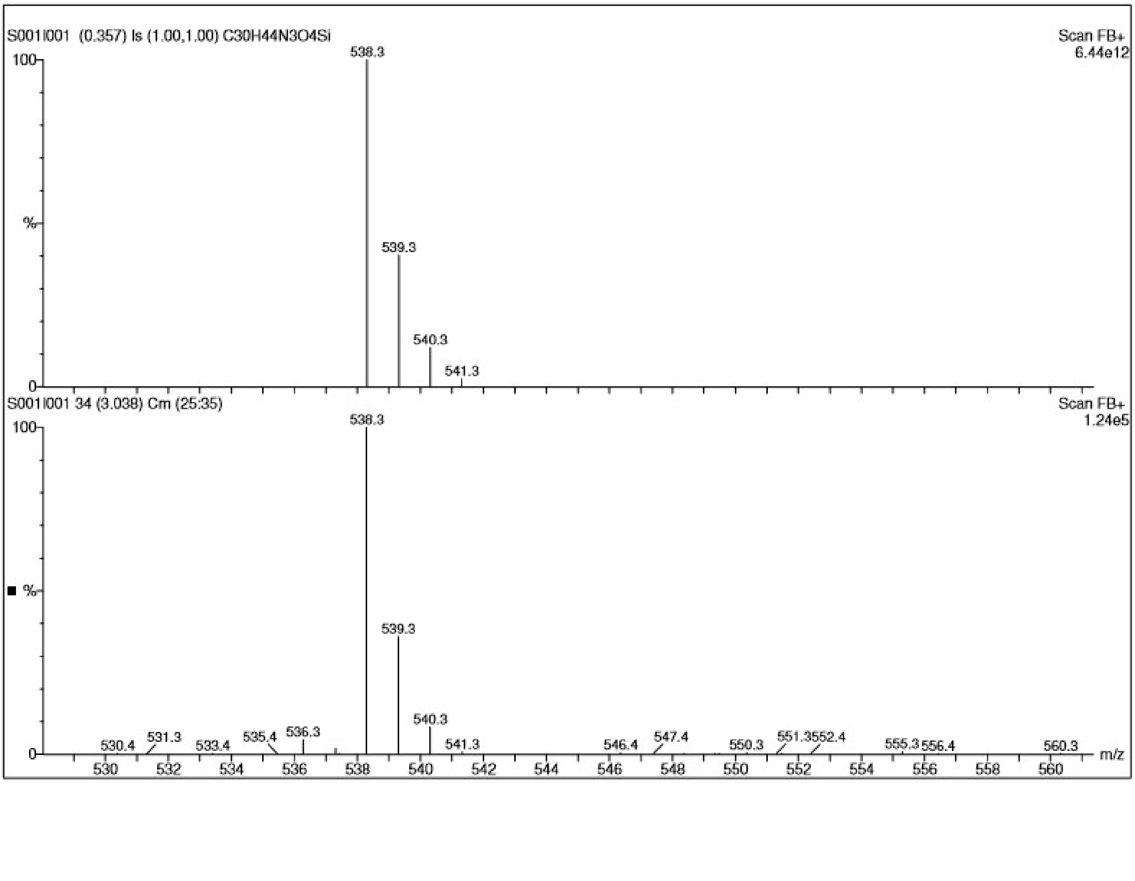
S001I001 (0.357) Is (1.00,1.00) C30H44N3O4Si
Scan FB+
6.44e12
538.3
539.3
540.3
541.3
S001I001 34 (3.038) Cm (25:35)
Scan FB+
1.24e5
538.3
539.3
540.3
541.3
530.4
531.3
533.4
535.4
536.3
546.4
547.4
550.3
551.3552.4
555.3 556.4
560.3
m/z

Compound 13. To a solution of compound **12** (270 mg, 0.5 mmol) in dry DMF (3 mL) were added iodomethane (36 mg, 0.25 mmol) and 1,8-diazabicycloundec-7-ene (40 mg, 0.25 mmol). The reaction mixture was heated at 55 ºC overnight. The solvent was removed under reduced pressure and the crude material was purified by column chromatography using DCM : MeOH 100 : 1 as a eluent. To give **13** as a colorless oil, 209mg (76%). ^{1}H NMR ([D6]dimethyl sulfoxide): 11.61 (br s, 1H), 7.60 (m, 4H), 7.43 (m, 6H), 3.64 (m, 4H), 3.09 (s,3H), 1.51 (m, 4H), 1.31 (m, 2H), 1.22 (br s, 12), 0.99 (s, 9H); ^{13}C NMR ([D6]dimethyl sulfoxide): δ= 150.2, 149.0, 148.7, 135.0 (4C), 133.4 (2C), 129.8 (2C),127.8 (4C), 63.4, 41.3, 40.3, 31.9, 28.9, 28.8, 28.7, 28.6, 28.0, 26.7 (2C), 26.1, 25.1, 18.8 (3C) ppm. MS m/z: calculated for $C_{31}H_{45}N_3O_4Si$ $[M+H]^+$ 552.3 found FAB 552.5.

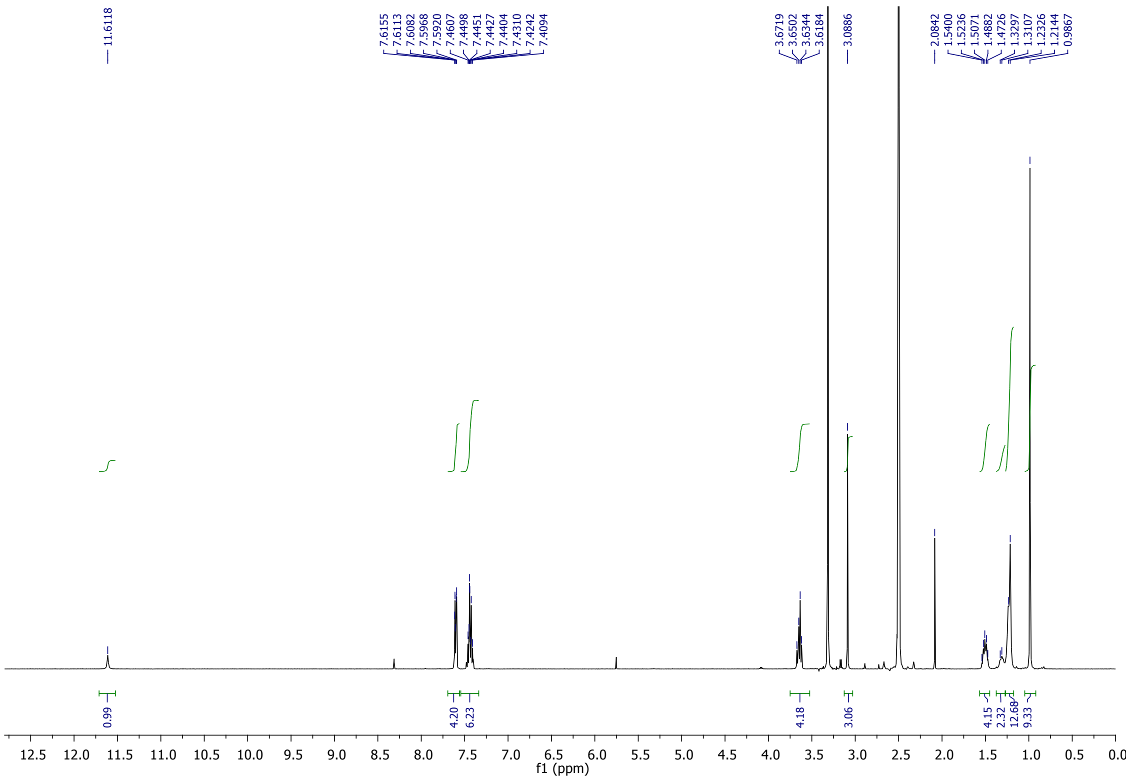

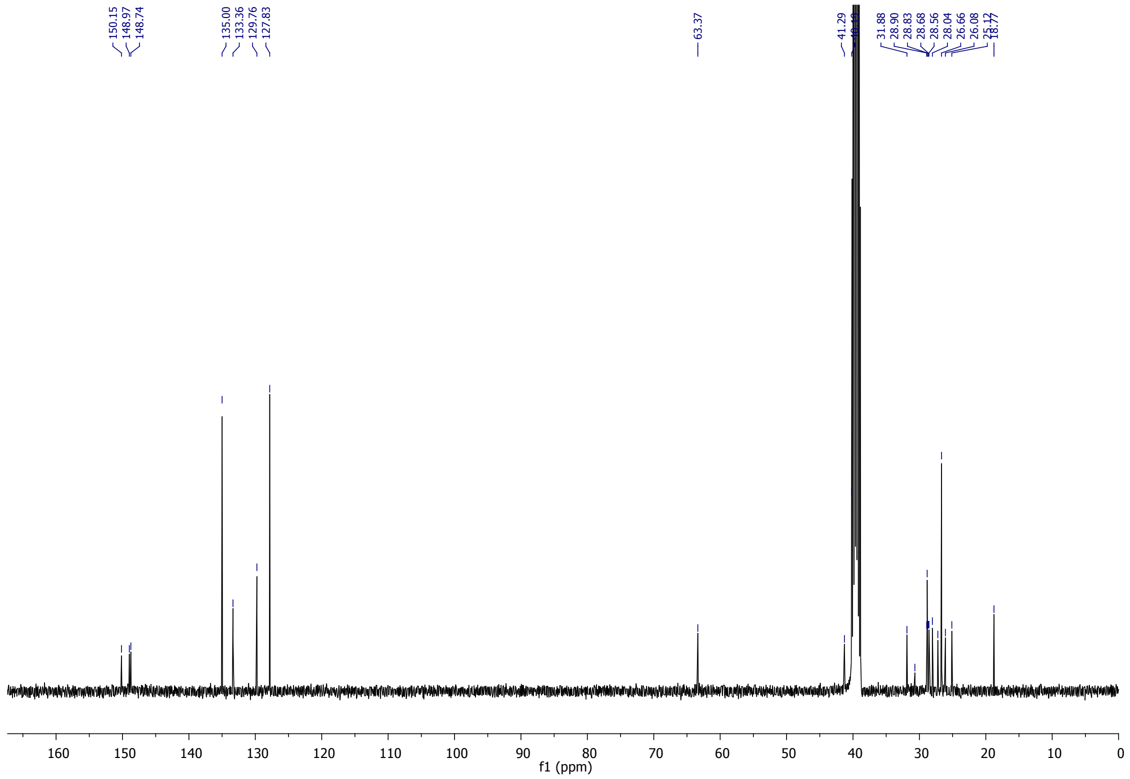
150.15
148.97
148.74
135.00
133.36
129.76
127.83
63.37
41.29
31.88
28.90
28.83
28.68
28.56
28.04
26.66
26.08
25.12
18.77
f1 (ppm)

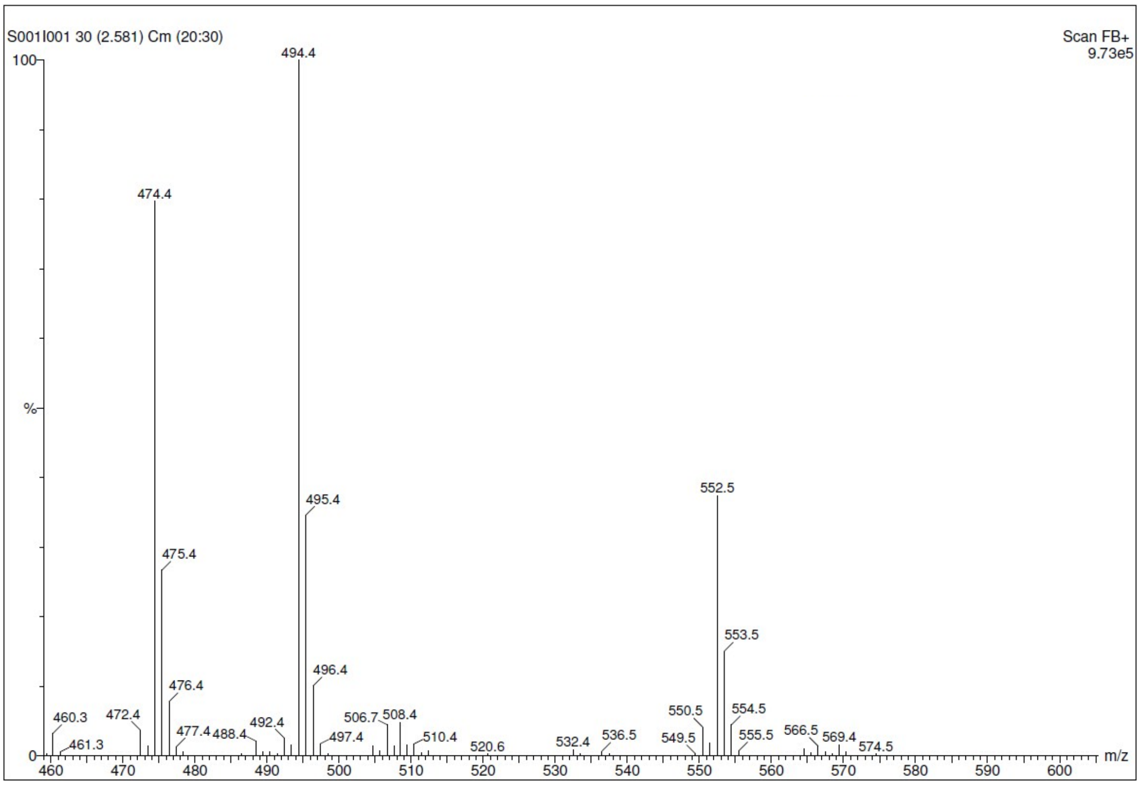
S001I001 30 (2.581) Cm (20:30)
Scan FB+
9.73e5
494.4
474.4
552.5
495.4
475.4
553.5
496.4
476.4
460.3
472.4
492.4
506.7
508.4
550.5
554.5
477.4
488.4
497.4
510.4
520.6
532.4
536.5
549.5
555.5
566.5
569.4
574.5
461.3
%
m/z

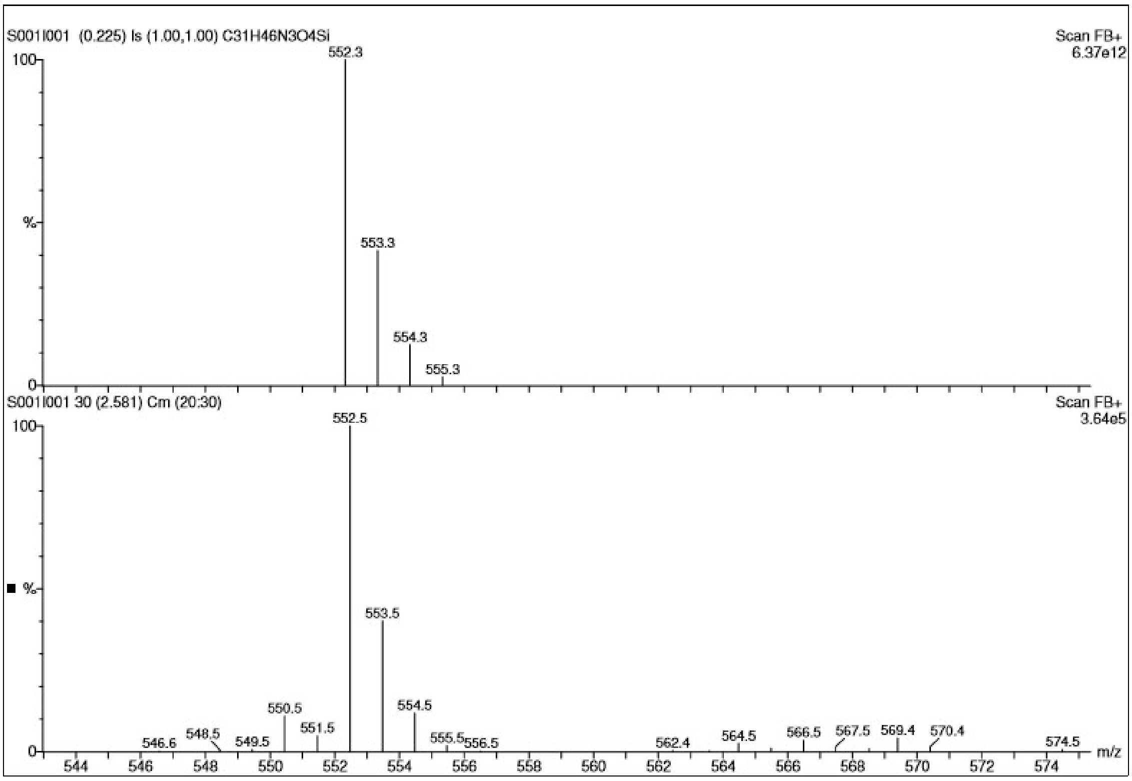

Compound 14. To a solution of **13** (120 mg, 0.22 mmol) in THF was added tetrabutylammonium fluoride solution 1.0 M in THF (0.28 mL). The reaction mixture was stirred for 1h at room temperature. Then the solvent was removed under reduced pressure and the crude material was purified by column chromatography (gradient elution DCM : MeOH 150 : 1 to 50 : 1) to give **14** as colorless oil, 63 mg, 91%. ^{1}H NMR ([D6]dimethyl sulfoxide): 11.61 (br s, 2H), , 4.30 (t, J = 5.1 Hz, 2 H), 3.37 (m, 2H), 1.51 (m, 2H), 1.40 (m, 2H), 1.24 (s, 14); ^{13}C NMR ([D6]dimethyl sulfoxide): δ= 150.2, 149.0, 148.7, 60.7, 41.3, 40.2, 32.5, 29.1, 28.9, 28.9 (2C), 28.7, 28.0, 27.2, 26.1, 25.5 ppm. MS m/z: calculated for $C_{15}H_{27}N_4O_4$ $[M+H]^+$ 314.4 found FAB 314.4.

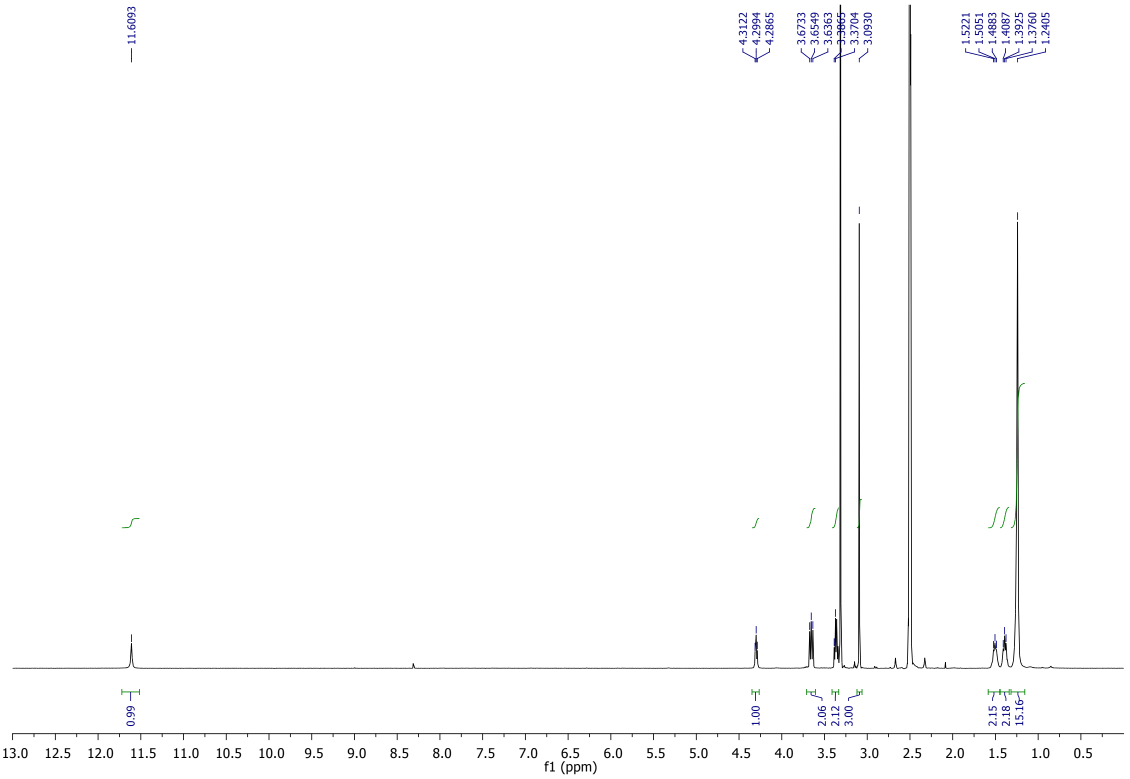
11.6093
4.3122
4.2994
4.2865
3.6733
3.6549
3.6363
3.3865
3.3704
3.0930
1.5221
1.5051
1.4883
1.4087
1.3925
1.3760
1.2405
0.99
1.00
2.06
2.12
3.00
2.15
2.18
15.16
f1 (ppm)

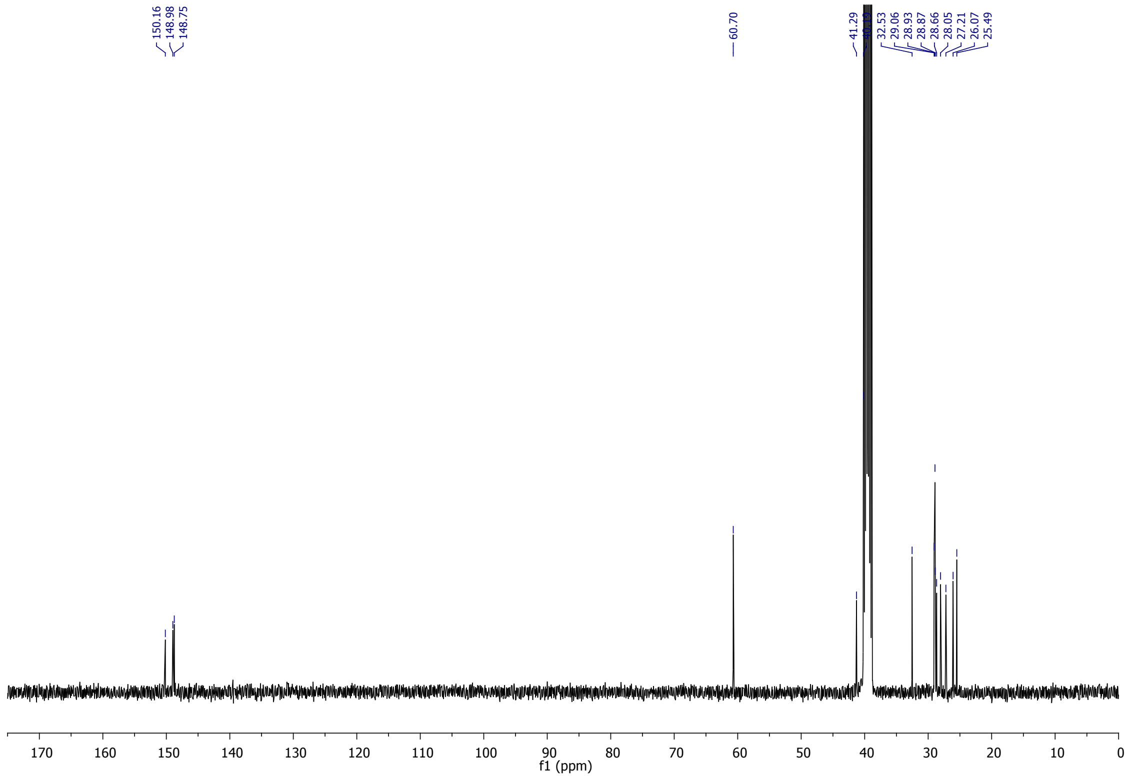
150.16
148.98
148.75
60.70
41.29
32.53
29.06
28.93
28.87
28.66
28.05
27.21
26.07
25.49
f1 (ppm)

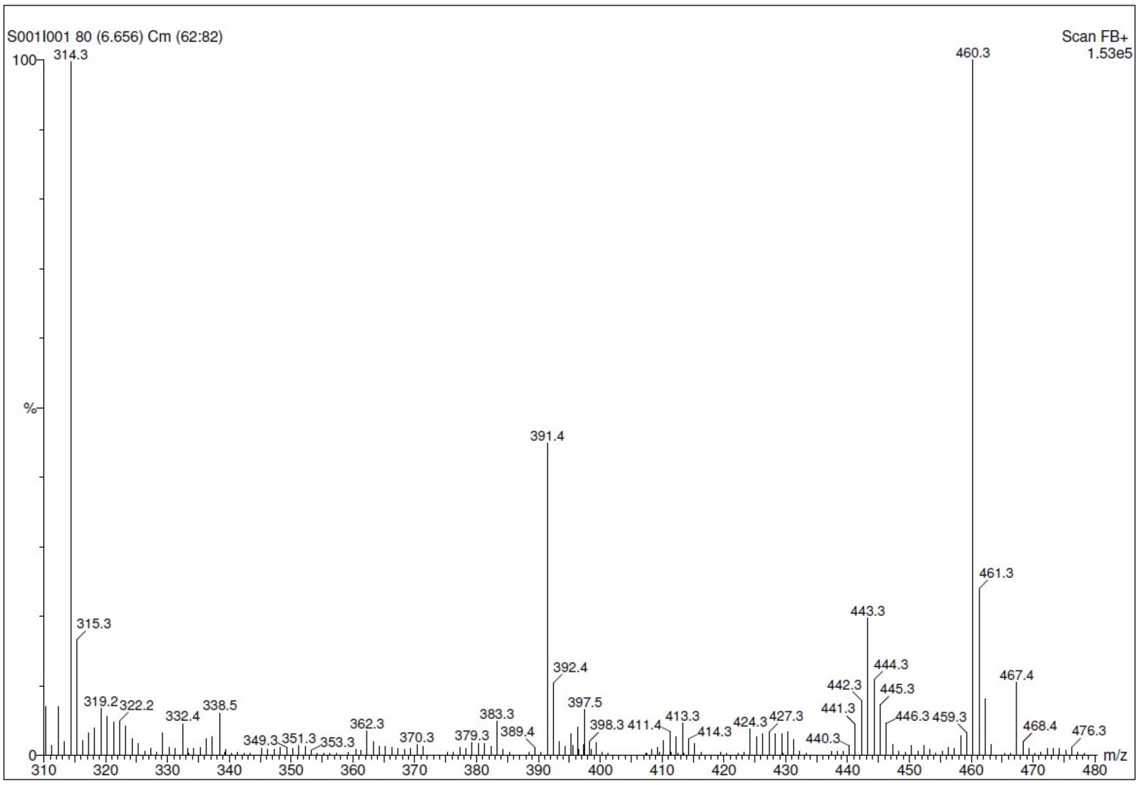
S001I001 80 (6.656) Cm (62:82)
Scan FB+
1.53e5
314.3
460.3
391.4
461.3
443.3
315.3
392.4
444.3
442.3
445.3
467.4
397.5
441.3
446.3
459.3
m/z

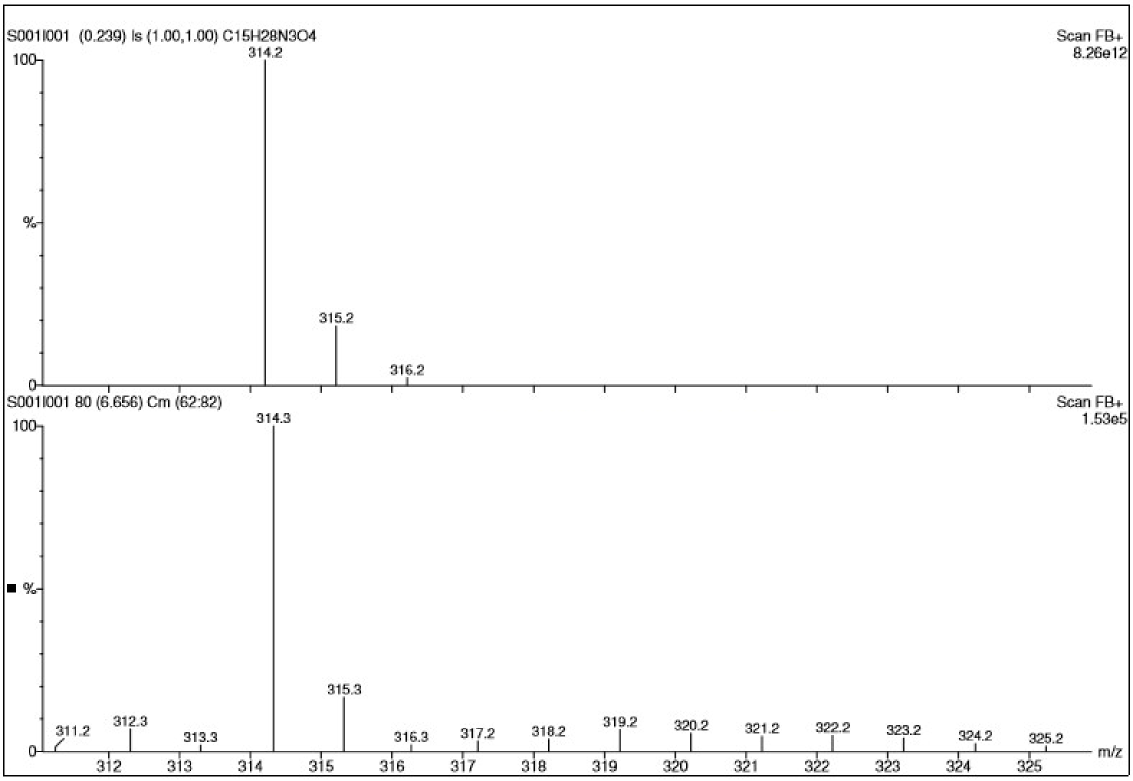
S001I001 (0.239) Is (1.00,1.00) C15H28N3O4
Scan FB+
8.26e12
314.2
315.2
316.2
S001I001 80 (6.656) Cm (62:82)
Scan FB+
1.53e5
314.3
315.3
m/z

Compound 15. Compound **14** (103 mg, 0.33 mmol) was solved in dry DMF (1 mL) and the solution was cooled to 0ºC. Then 5-Benzylthio-*1-H*-tetrazole (76 mg, 0.4 mmol) and the phosphitylating reagent, 2-Cyanoethoxy-bis(N,N-diisopropylamino) phosphine (100 mg, 0.33 mmol), were added. The reaction mixture was allowed to stir at room temperature for 3h under argon atmosphere. When the reaction was completed (followed by TLC), the crude material was using directly, without further purification, in the last step of the automated solid phase synthesis of the corresponding oligonucleotides (oligonucleotide **5** and oligonucleotide **8**), due to compound **15** is a very unstable product, we were not able to isolate it.

4. Automated Oligonucleotide synthesis and analytical data of oligonucleotides.

The syntheses of oligonucleotides were performed on a MerMade 4 synthesizer (BioAutomotion Corporation). For each oligonucleotide synthesis, columns filled with the corresponding Controlled Pore Glass (CPG) solid support. Anhydrous MeCN was used as solvent. For the cleavage of DMTr protecting groups, the resin was purged with 3 % trichloroacetic acid in anhydrous CH_2Cl_2. The removal of the acid was carried out by purging with anhydrous MeCN. The activation of the phosphoramidite functionality was effected by a 0.25 M benzylthiotetrazole solution in anhydrous MeCN. The coupling time for standard phosphoramidites was 2 min and for cyanuric-acid derivatives 5 min (compound **11** and **15**). Oxidation of P(III)-species was attained by alkaline iodine solution (20 mM I_2 in THF/Py/water 7/2/1). For the capping of residual 5'-OH-groups, a mixture of solution A (10% Ac_2O, 10% pyridine, 80% THF) and solution B (10% 1-methylimidazole in THF) was used. After completion of the synthesis, the oligonucleotides were cleaved from the solid support with concomitant removal of the Fmoc and β-cyanoethyl protecting groups by reacting the oligonucleotide-charged solid support with 28 % aq. NH_3 at 55 °C for 20 h. The solution was filtered and the filtrate was concentrated in vacuo. The residue was dissolved in 750 µL water. For purification of this crude oligonucleotide solution, a volume containing ~ 40 nmol crude oligonucleotide was applied to gel electrophoresis

(1 mm, 20% polyacrylamide). The oligonucleotide-containing segments of the gel were visualized by UV-light (260 nm) and separated from the rest of the gel. Oligonucleotides were extracted from the gel using an elutrap system (3 h, 200 V). The solutions were desalted using a NAP-10 column and concentrated in an evaporating centrifuge.

5. Mass spectral data of synthesized oligonucleotides

Maldi data was obtained at the Proteomic facility of the National Center for Biotechnology (CNB-CSIC)

no.	Sequence	Calculated	Found
1	5´-TCGA ATC AGT TTA CAA AAA-3´	5803.9	5811.3
2	5´-TTT TTG TAA ACT GAT TCGA-3´	5807.8	5812.1
3	5´-TCGA ATC AGT TTA CAA AAA-HR-3´	6699.8	6696.1
4	5´-cy-TTT TTG TAA ACT GAT TCGA-3´	6169.1	6165
5	5´-cyCH_3-TTT TTG TAA ACT GAT TCGA-3´	6183	6182
6	5´-P-TCGA ATC AGT TTA CAA AAA-HR-3´	6776.7	6777.2
7	5´-cy-TTT TTG TAA ACT GAT-3´	4933.3	4935.5
8	5´-cyCH_3-TTT TTG TAA ACT GAT-3´	4947	4945.6
9	5´-TTT TTG TAA ACT GAT-3´	4572	4570.4

Table S1. Maldi data of synthesized oligonucleotides

1 5´-TCGA ATC AGT TTA CAA AAA-3´ Calc. 5803.9 Found. 5811.3

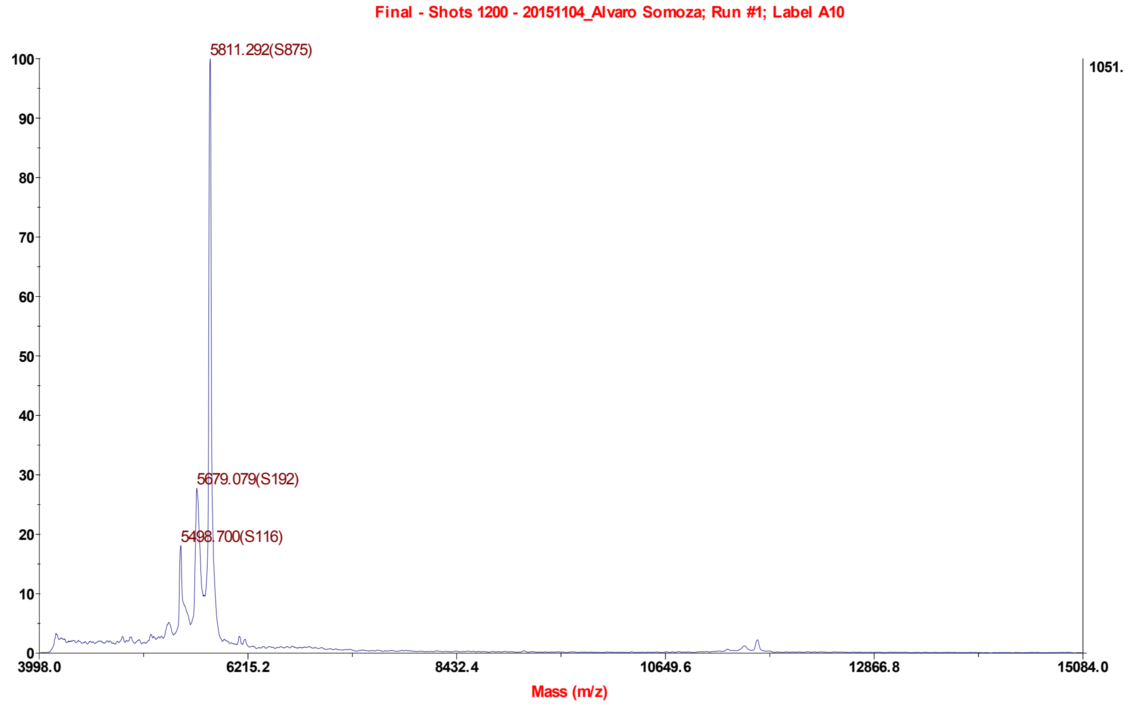

2 5´-TTT TTG TAA ACT GAT TCGA-3´ Calc. 5807.8 Found. 5812.1

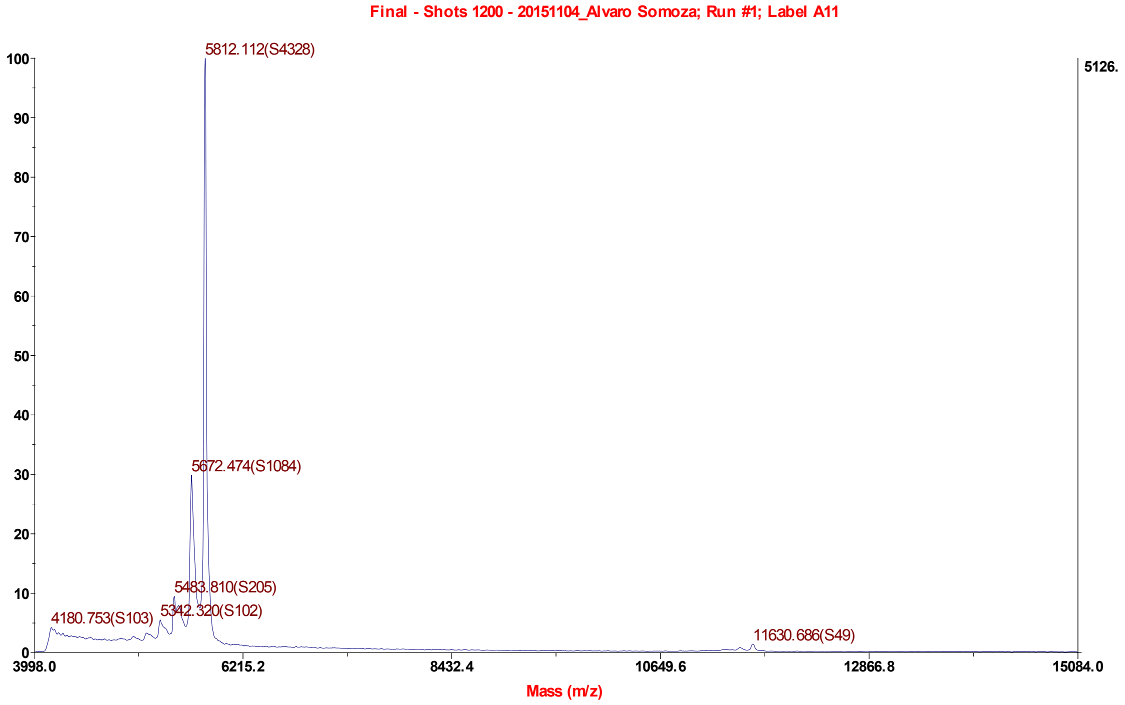

3 5´-TCGA ATC AGT TTA CAA AAA-HR-3´ Calc. 6699.8 Found. 6696.1

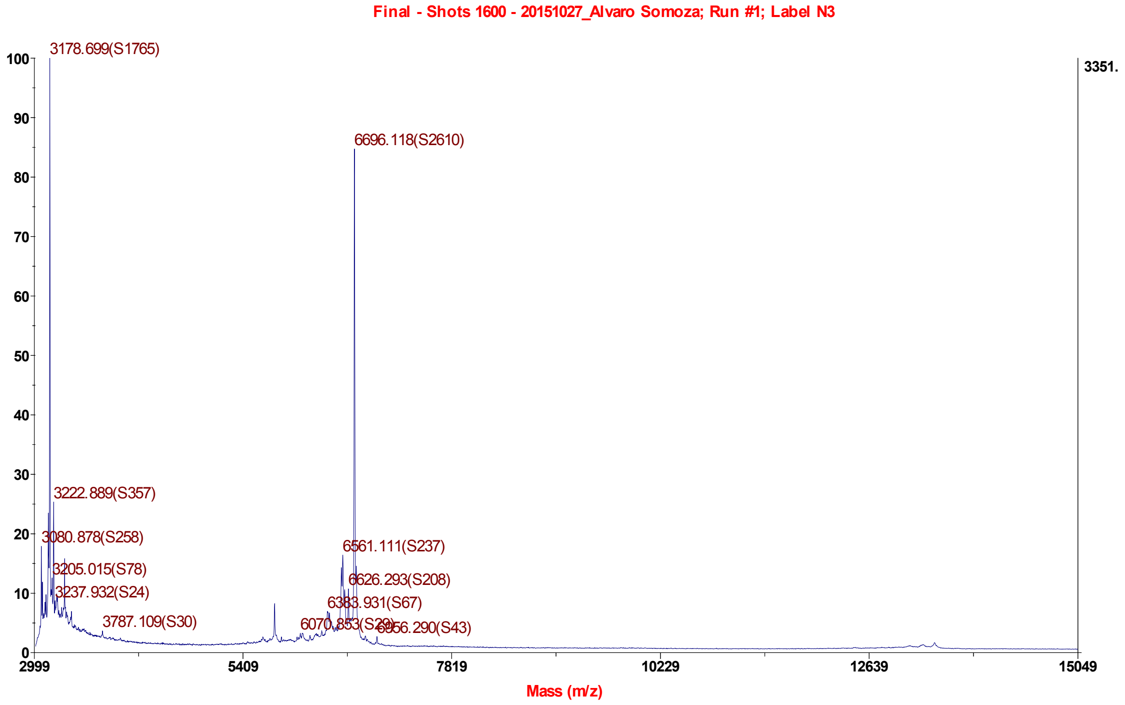

4 5´-cy-TTT TTG TAA ACT GAT TCGA-3´ Calc. 6169.1 Found. 6165

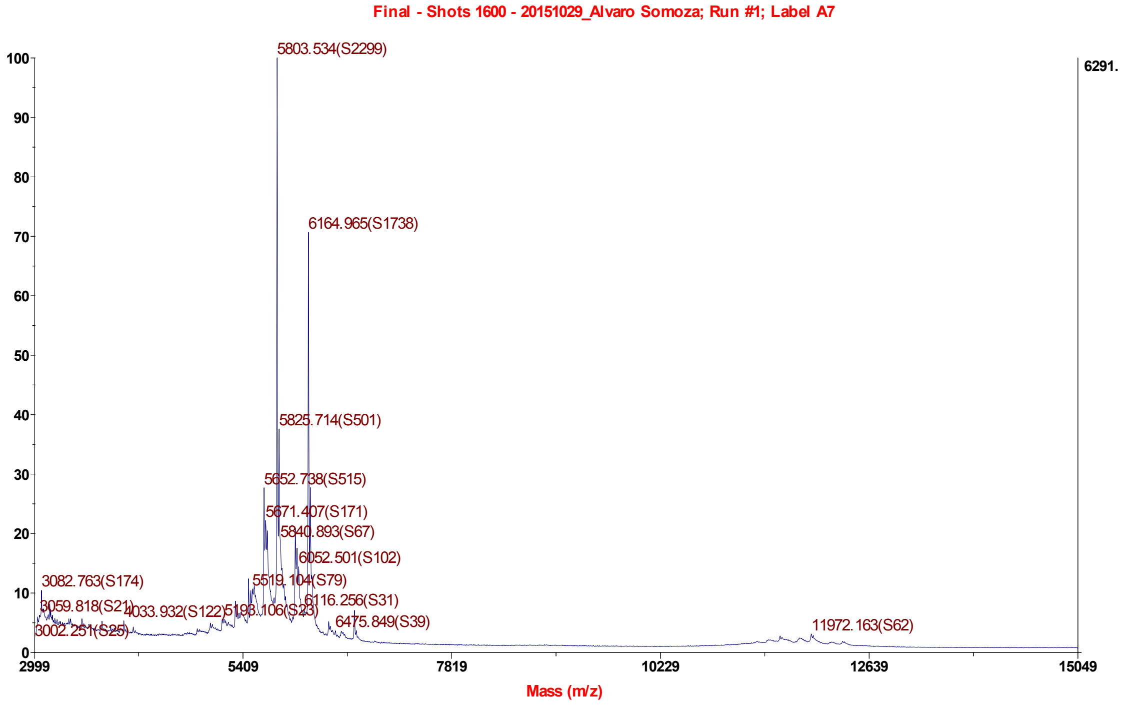

5 5´-cyCH$_3$-TTT TTG TAA ACT GAT TCGA-3´ Calc.6183 Found. 6182

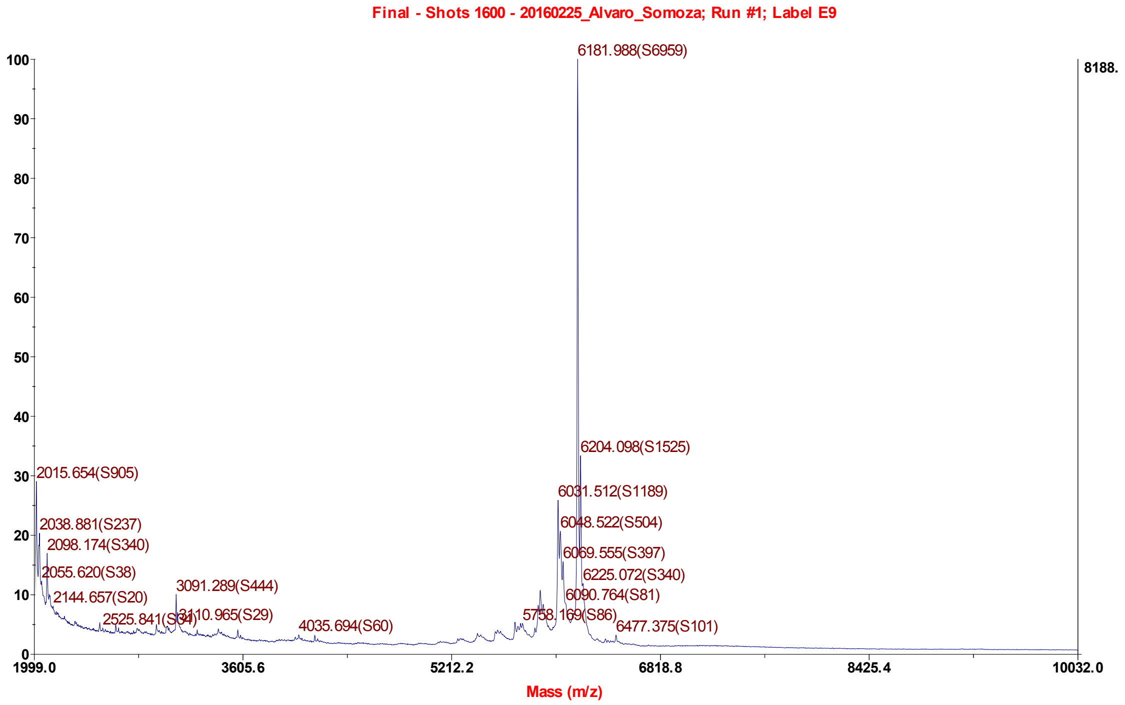

6 **5´-P-TCGA ATC AGT TTA CAA AAA-HR-3´** **Calc. 6776.7** **Found. 6777.2**

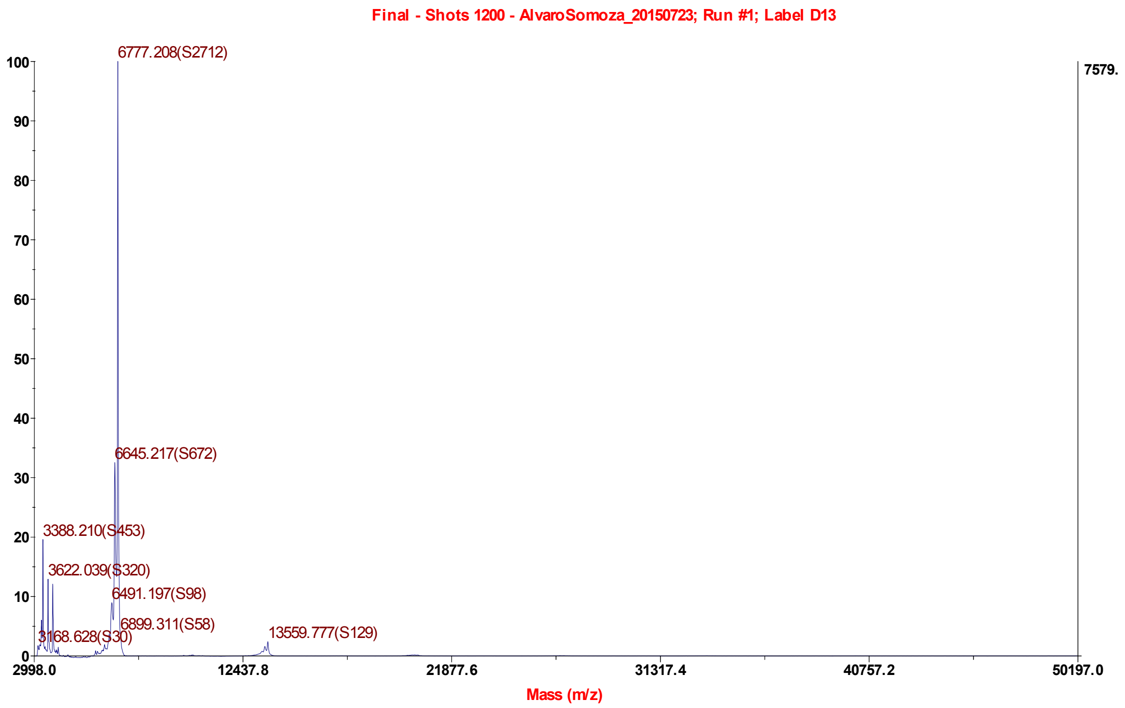

7 **5´-cy-TTT TTG TAA ACT GAT-3´** **Calc. 4933.3** **Found. 4935.5**

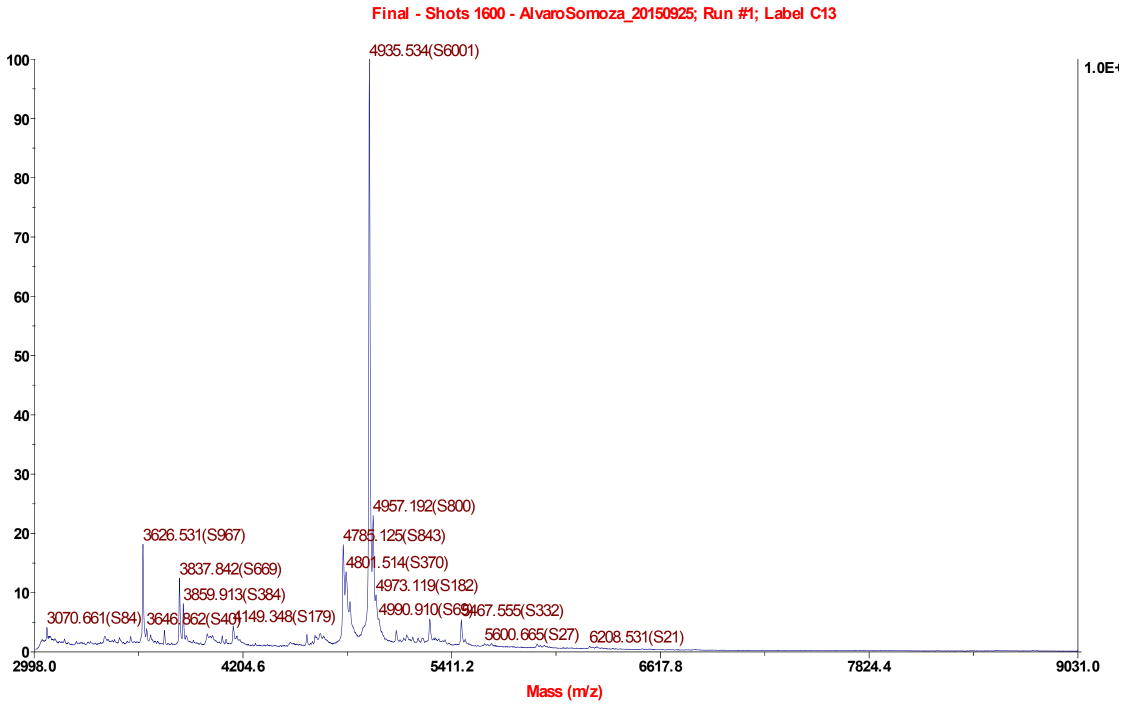

8 5´-cyCH_3-TTT TTG TAA ACT GAT-3´ Calc. 4947 Found. 4945.6

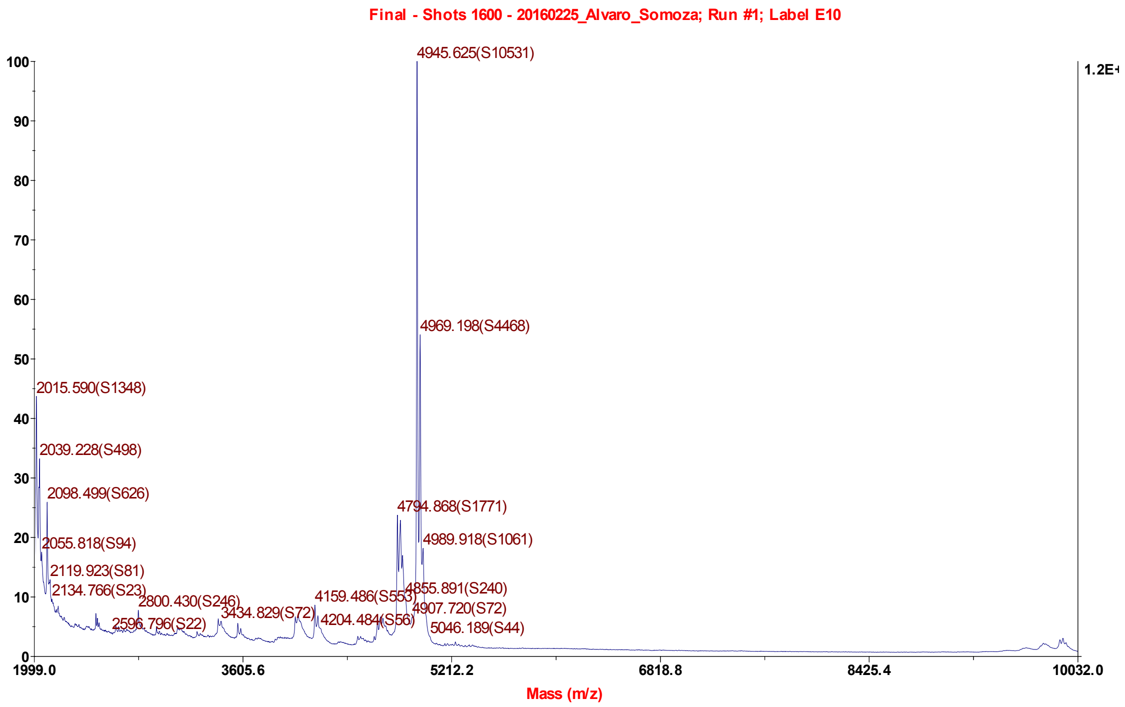

9 5´-TTT TTG TAA ACT GAT-3´ Calc. 4572 Found. 4570.4

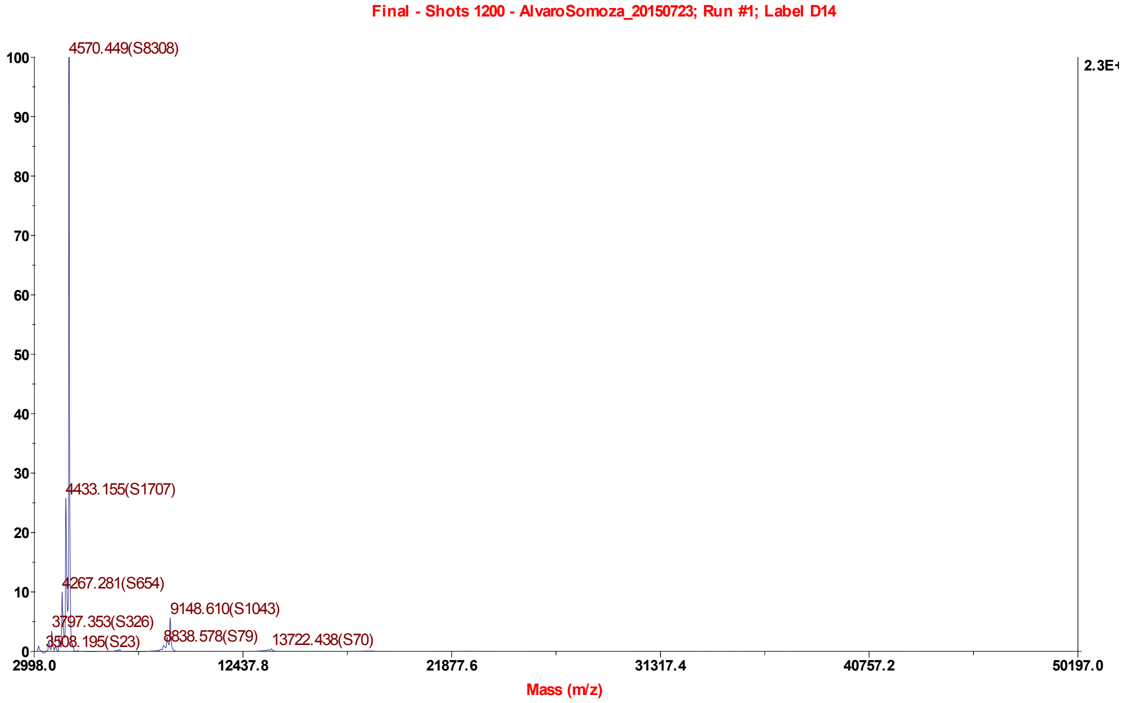

6. Melting temperatures of duplexes

Melting curves of duplexes were measured on a Cary 5000 UV–Vis-NIR spectrophotometer. For these measurements, buffered aqueous solutions of oligonucleotides were prepared. Phosphate Buffered Saline (PBS) (pH=7, 10 mM NaH_2PO_4/Na_2HPO_4 1:1, 150 mM NaCl) with oligonucleotide concentrations of 0.2 µM. The volume of each sample was 1000µL. The samples were heated at a rate of 1 °C/min. For each sample, three melting curves were recorded at a wavelength of 260 nm. Melting temperatures were obtained by calculating the inflexion points of the melting curves.

The melting point was determined for the duplexes a-f, which have no free base pairs (in spite of measuring the melting temperature of the duplexes that were ligated to form the hairpin DNA structure).

Duplexes b and c reveal an increment in the melting point of 3.9 and 2.6 ºC, this fact is due to the 6 and 4 hydrogen bonds that end up each duplex.

All Tm and ΔTm values are reported in °C.

ΔTm values are referring to duplex a, which has no modifications.

no.	Sequence	Tm	ΔTm
**a**	5´-TCGA ATC AGT TTA CAA AAA-3´<br>5´-TTT TTG TAA ACT GAT TCGA-3´	56.3 ± 0.2	3.9
**b**	5´-TCGA ATC AGT TTA CAA AAA-HR-3´<br>5´-cy-TTT TTG TAA ACT GAT TCGA-3´	60.2 ± 0.3	0
**c**	5´-TCGA ATC AGT TTA CAA AAA-HR-3´<br>5´-cyCH_3-TTT TTG TAA ACT GAT TCGA-3´	58.9 ± 0.4	2.6
**d**	5´-TCGA ATC AGT TTA CAA AAA-HR-3´<br>5´-TTT TTG TAA ACT GAT TCGA-3´	55.9 ± 0.2	0.4
**e**	5´-TCGA ATC AGT TTA CAA AAA-3´<br>5´-cy-TTT TTG TAA ACT GAT TCGA-3´	56.2 ± 0.4	0.1
**f**	5´-TCGA ATC AGT TTA CAA AAA-3´<br>5´- cyCH_3-TTT TTG TAA ACT GAT TCGA-3´	56.9 ± 0.3	0.6

Table S2. Melting temperatures of oligonucleotide duplexes.

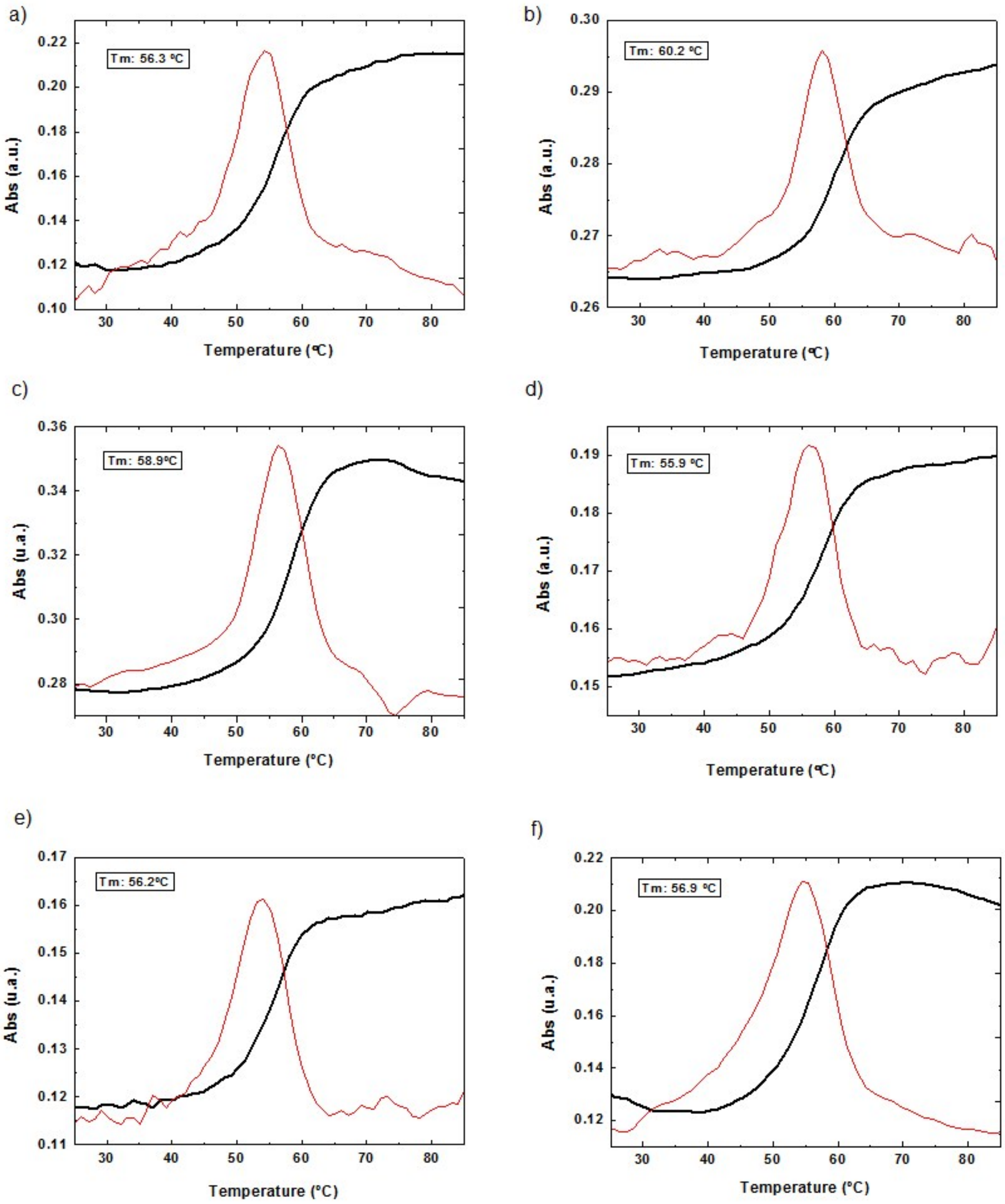

Figure S1. Melting temperatures of oligonucleotide duplexes a-f.

7. Synthesis of the DNA construct

The main DNA unwinding segment (410 bp) contains one, two and three repetitions of the GCC cluster separated by 97 bp of a low GC content sequence (Genscript Corp.) The DNA unwinding segment was digested with *EcoRI* and *SalI* restriction endonucleases. The *SalI* end was ligated to the previously described oligonucleotides modified with the HR-cy couple or to a self-annealing oligonucleotide forming a penta-loop (to test the strength of the attachments, see Figure S3). The *EcoRI* end was ligated to the *EcoRI* end of a short dsDNA linker; the other end of the linker contains non-complementary 5' and 3' protruding strands. The 5' strand is labelled with biotin and the 3' contains the *PstI* restriction endonuclease recognition sequence. After purification of ligation products (Qiagen PCR Purification Kit) a 2686 dsDNA handle (puc19 vector, Novagen) cut with *PstI* and labeled with Digoxigenin at the 5' end was ligated to the *PstI* 3' protruding end of the linker.

8. Optical tweezers experiments

The DNA construct was bound to Anti-Dig covered beads (Spherotech Co.) by incubating 3 μl of the DNA preparation with 3 μl of the beads for ten minutes at room temperature. The sample was then diluted with 300 μl of the reaction buffer (Tris-HCl 20 mM pH 7.5, 50 mM NaCl) and flowed into the flow chamber. The Anti-Dig, DNA covered beads were manipulated with the optical trap and single DNA constructs were obtained by attaching the biotin end of the DNA to the streptavidin bead (Spherotech Co.) previously hold on top of a mobile micropipette. The mechanical properties of the DNA construct allowed identifying single attachments (see experimental force-extension curves below). Data were collected in a dual-beam optical tweezers at 100 Hz at 22 ± 1 °C. Pulling rates were 50 and 200 nm/ s, when indicated.

9. Experimental force-extension curves

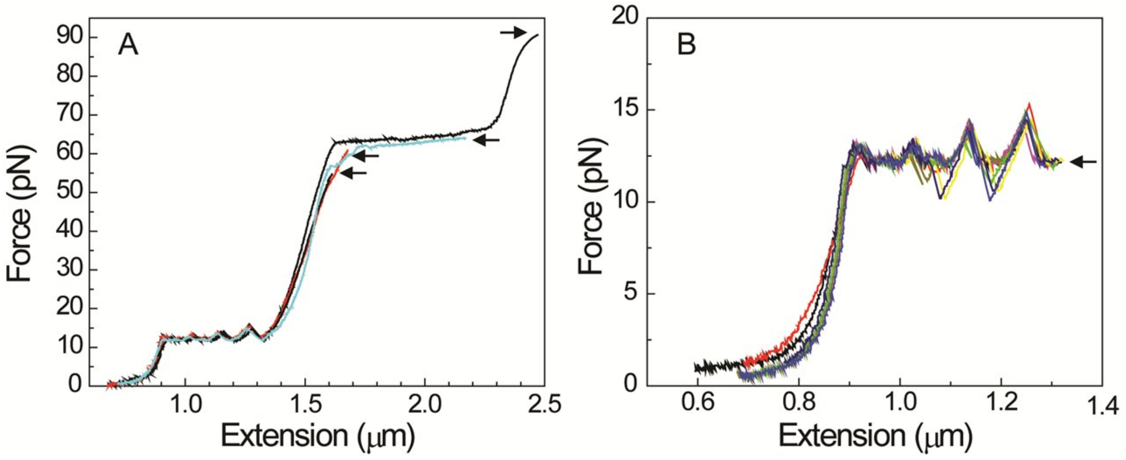

Figure S2. A) Independent force-extension curves (FECs) of DNA constructs covalently closed at the *SalI* end by a self-annealing oligonucleotide (see above). Rupture forces (black arrows) indicate the strength of the Dig-AntiDig and/or Biotin/Streptavidin connections used to attach the DNA between the beads (Figure 1, main text). The increase in extension at ~60 pN corresponds to the overstretching transition of the dsDNA handle (reference 16 main text). **B**) Alignment of independent FECs without the HR component at the *SalI* end. Disassembly (or rupture force, black arrow) occurred after unzipping of the last GC cluster position. For A) and B) the pulling rate was 50 nm/s.

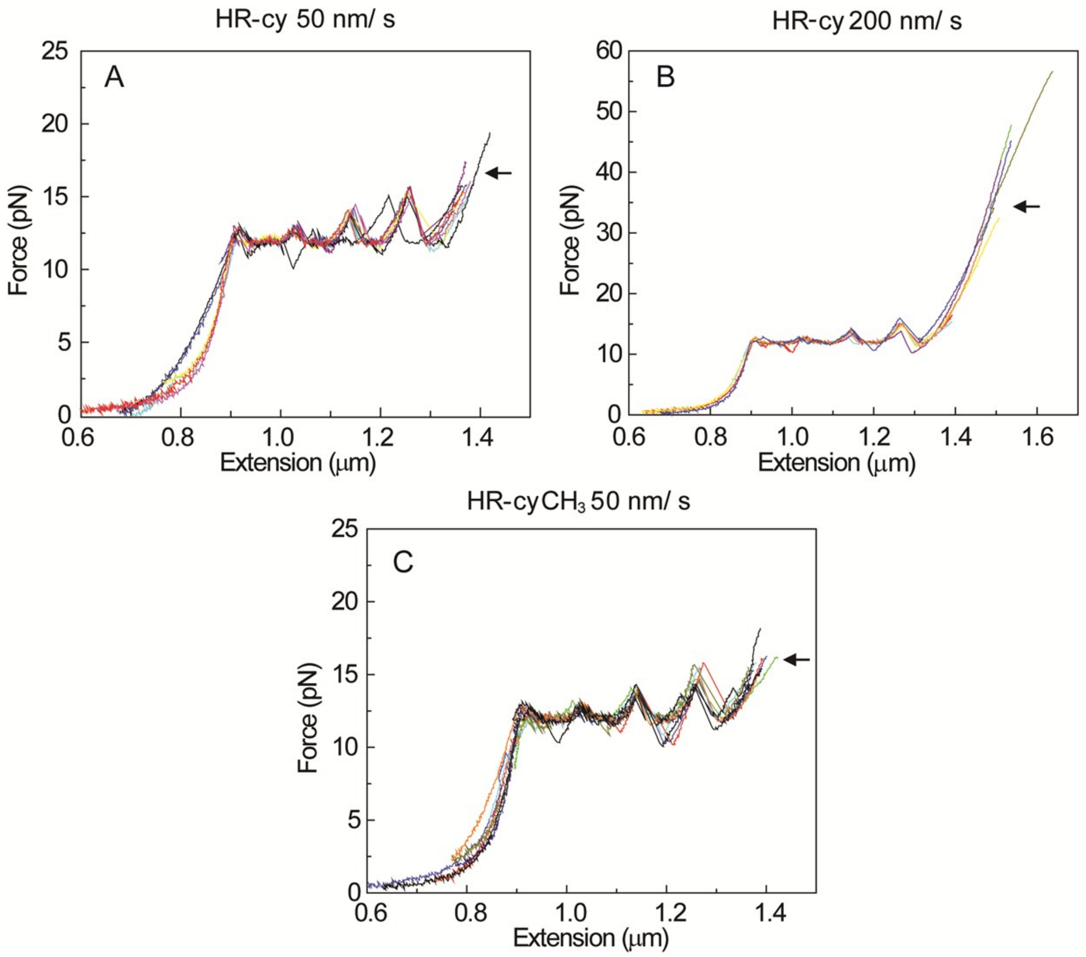

Figure S3. Alignment of independent FECs of DNA constructs harbouring at the *SalI* end the HR-cy couple (A, pulling rate 50 nm/ s and B, pulling rate 200 nm/ s) or the HR-cyCH_3 couple (C, pulling rate 50 nm /s). For all plots arrows show the average rupture force at each condition: A) 16.7 ± 1.6 pN, B) 34.0 ± 9.0 pN and C) 15.6 ± 0.7 pN.

10. Statistical analysis

In order to test quantitatively whether the differences between the values of the rupture forces reported in our work are statistically significant, we applied the statistical hypothesis test known as t-test. The t-test is commonly used to determine whether two independent sets of data are significantly different from each other (Boslaugh S and Watters PA (2008). “The t-test” in Statistics in a nutshell. O’Reilly, 151-165).

As the two samples have unequal sizes and unequal variances, the t statistic to test whether the population averages are different is known as Welch t-test and is calculated as

$$t = \frac{\bar{X}_1 - \bar{X}_2}{\sqrt{s_{1,m}^2 + s_{2,m}^2}},\ (1),$$

where $\bar{X}_1$ is the mean of the sample 1, and $s_{1,m}^2 = s_1^2/n_1$ is the square of the standard deviation of the mean, with s_1 the sample standard deviation and n_1 the size of the sample 1. Analogously, the same quantities with sub-index 2 are defined for the sample 2. For use in significance testing, the distribution of the test statistic is approximated as an ordinary Student's t-bilateral-distribution with the degrees of freedom (*df*) calculated as

$$df = \frac{\left(s_{1,m}^2 + s_{2,m}^2\right)^2}{s_{1,m}^4/(n_1 - 1) + s_{2,m}^4/(n_2 - 1)} - 2,\ (2).$$

Once the t value and degrees of freedom are determined, a *p*-value can be found using a table of values from Student's t-bilateral distribution. The *p*-value is defined as the probability of obtaining a result equal to or more extreme than what was actually observed. For example, if the *p*-value obtained is 0.1, the probability of the two data sets to be equal ($\bar{X}_1 \pm s_{1,m} = \bar{X}_2 \pm s_{2,m}$) is 10%. In other words, it can be affirmed with a 90% probability that the two data sets are different ($\bar{X}_1 \pm s_{1,m} \neq \bar{X}_2 \pm s_{2,m}$).

As mentioned above, we run the t-test to determine if the differences between the rupture forces measured for the HR-cy couple at different conditions are ‘real‘ or statistically significant. Table S1 below shows the averages and uncertainties of the rupture forces (F_{rup}) measured for each DNA construct (or HR-cy couple) and pulling rate. wt^{50}: DNA construct with the HR-cy couple, pulling rate 50 nm/ s. c^{50}: DNA construct without the HR-cy couple, pulling rate 50 nm/ s. CH_3^{50}: DNA construct with the HR-cyCH_3 couple, pulling rate 50 nm/ s. wt^{200}: DNA construct with the HR-cy couple, pulling rate 200 nm/ s.

The results of the test indicate that the average rupture forces measured for the HR-cy and HR-cyCH_3 couples are statistically different with a ~95 % probability (highlighted in blue in Table S1).

X_1 vs X_2	wt^{50} vs c^{50}	wt^{50} vs $CH_3{}^{50}$	wt^{50} vs wt^{200}
F_{rup} X_1	16.7 ± 1.6	16.7 ± 1.6	16.7 ± 1.6
F_{rup} X_2	11.5 ± 0.3	15.6 ± 0.7	34.4 ± 8.5
F_{rup} X_1 – F_{rup} X_2	5.2 ± 0.46	1.1 ± 0.51	17.7 ± 2.87
p-value F_{rup} X_1 ≠ F_{rup} X_2	10^{-7}	0.046	0.0001
Probability (%) of F_{rup} X_1 ≠ F_{rup} X_2	99.99	95.35	99.98

Table S1. Statistical analysis of the differences between the rupture forces.

11. Computational details

All theoretical calculations were carried out within the density functional theory (DFT) approach by using the C.01 revision of the Gaussian 09 program package.[1] DFT calculations were performed using the long-range corrected ωB97X-D[2-3] density functional, which are able to incorporate the dispersion effects by means of a pair-wise London-type potential. The ωB97X-D density functional has emerged as a robust and powerful density functional able to provide accurate structures in supramolecular aggregates dominated by non-covalent interactions of different nature. A Polarizable Continuum Model (PCM) using the integral equation formalism variant (IEFPCM) was used to represent solvent around solutes via a set of overlapping spheres. This level of DFT has been successfully used for the description of non-covalent interactions in this family of synthetic receptors.[4] The double-zeta Pople's 6-31G(d,p)[5] basis set was employed throughout and the basis set superposition error (BSSE) was corrected according to the counterpoise (CP) scheme of Boys and Bernardi.[6]

The optimized geometry of HR-cy and HR-cyCH_3 at ωB97X-D/6-31G (d,p) level of theory are shown in figure S4. The structure of the system was simplified by substituting the alkys chain attached to the Hamilton receptor by an hydrogen atom and the alkys chain attached to the cyaniric acid guest by a shorter chain of $–(CH_2)_2-CH_3$ moiety. As scan coordinate we define the distance between the carbonyl moiety of the cy and the benzene moiety in the HR. In the figure the initial optimized structures of both studied system are depicted and also the graphical definition of the scan coordinate.

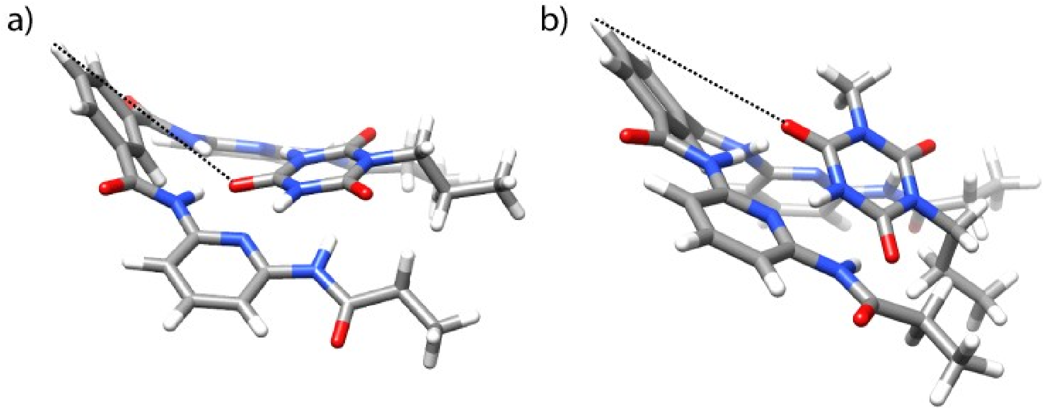

Figure S4. A) Optimized structure of HR-cy and B) HR-cyCH_3 systems at ωB97X-D /6-31G (d,p) level of theory. In dotted line the scan coordinate is defined.

The potential energy curves (PECs) for HR-cy and HR-cyCH_3 couples were calculated using the SCAN tool implemented in Gaussian software, where single point energy evaluations were performed in each selected internal coordinates. The potential energy curves (PECs) for HR-cy and HR-cyCH_3 in two different solvents: water and o-dichlorobenzene are displayed in Figure S5. In the same figure we show the optimized structures for in the initial stage (completely bonded) and non-interacting state, using a continuous model of water.

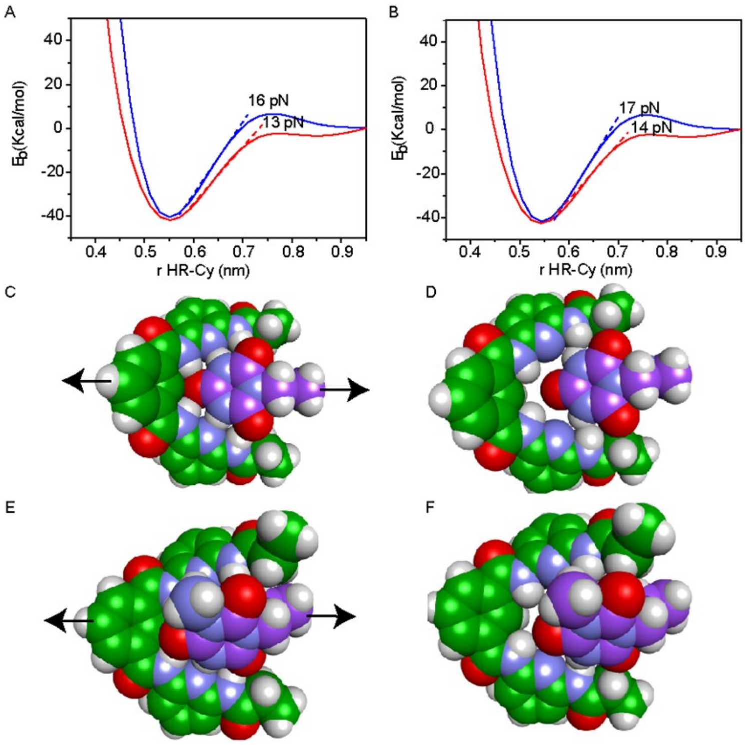

Figure S5. A) Potential energy curves for HR-cy (blue) and HR-cyCH3 (red) using a continuous model of water and B) o-dichlorobenzene. Energy-minimized molecular models showing the geometry of the C) bound and D) unbound HR-cy. Energy-minimized molecular models showing the geometry of the E) bound and F) unbound HR-cyCH_3.

12. Cartesian coordinates of molecular optimized geometries of HR-cy and HR-cyCH_3 systems at ωB97X-D /6-31G (d,p) level of theory.

<u>HR-cy wb97xd/6-31g(d,p) scrf=(iefpcm,solvent=water)</u>

0 1

O	-0.01442500	1.08558400	-0.27699300
C	0.00002000	-0.01905700	0.25065100
N	1.16700100	-0.65072600	0.57686200
N	-1.15017100	-0.69107700	0.55563500
C	1.24421900	-1.88494200	1.17748300
H	2.05880500	-0.17340000	0.29640900
C	-1.19601900	-1.93174700	1.14630500
H	-2.05319100	-0.23747900	0.27256700
N	0.03122500	-2.48306400	1.48585900
O	2.31195800	-2.41931200	1.42242800
O	-2.24929500	-2.50700400	1.35886000
C	0.05306600	-3.82757000	2.08041600
C	0.19728700	-4.92037000	1.02560300
H	-0.88133600	-3.93993200	2.63054000

H	0.88675500	-3.85262400	2.78345800
C	0.17141400	-6.30926500	1.65796900
H	1.13851100	-4.76958300	0.48675300
H	-0.61792100	-4.82298400	0.30008300
H	-0.77600300	-6.48868200	2.17586400
H	0.98076100	-6.42795300	2.38530600
H	0.28879600	-7.08498900	0.89772000
C	-0.06454800	6.16677300	1.85714300
N	3.56429200	0.63779000	-0.35678200
C	3.64833300	1.95773700	-0.59215200
C	4.59251200	-0.14896700	-0.72015100
C	4.75649900	2.54763200	-1.19728700
N	2.51934800	2.69392100	-0.21340100
N	4.43204900	-1.49443900	-0.38891800
C	5.73578300	0.34635900	-1.34907600
C	5.79632900	1.71203900	-1.57653000
H	4.78774200	3.61513700	-1.35035800
C	2.49756400	4.03629600	0.10412700
H	1.66638600	2.15496000	-0.09527900
C	5.19104700	-2.56648900	-0.81464200
H	3.70753700	-1.70823900	0.29266200
H	6.52535700	-0.33053600	-1.63677400
H	6.67238000	2.13489500	-2.05738000
C	1.15702300	4.56811300	0.52397700
O	3.48685500	4.74794600	0.10055700
C	4.73958600	-3.89099700	-0.21445700
O	6.12314300	-2.47830800	-1.59148500
C	-0.04855500	4.03707700	0.06817000
C	1.14409800	5.64616900	1.40996700
C	5.54882900	-5.06911200	-0.73662900
H	3.67113100	-4.02059500	-0.42261800
H	4.81641300	-3.81214600	0.87645500
C	-1.26147100	4.53474000	0.54143300
H	-0.04319000	3.25635400	-0.68072100
H	2.09097800	6.06347100	1.73463000
H	5.43617600	-5.17238500	-1.81802600
H	5.21761500	-5.99655500	-0.26264100
H	6.61215900	-4.93168800	-0.53072300
C	-1.26471800	5.61251200	1.42811400
C	-2.59359300	3.96968000	0.13824200
H	-0.07100600	7.00319300	2.54787600
H	-2.21759900	6.00368900	1.76743900
N	-2.58619800	2.63059100	-0.19425600
O	-3.60090600	4.65542800	0.15757400
C	-3.70244700	1.87313000	-0.57027100
H	-1.71921900	2.11092500	-0.09214800
C	-4.83011100	2.44701500	-1.15454300
N	-3.58710900	0.55207100	-0.35398200
C	-5.85683400	1.59498200	-1.53237500
H	-4.88616300	3.51538200	-1.29312200
C	-4.60399500	-0.25105900	-0.71462500
C	-5.76508500	0.22834500	-1.32317000
H	-6.74702200	2.00560000	-1.99761300
N	-4.41314700	-1.59695500	-0.40256000
H	-6.54389800	-0.46140400	-1.60946500
C	-5.16948200	-2.67665400	-0.81388400
H	-3.66925500	-1.80601100	0.25925200
C	-4.69453600	-3.99663400	-0.22209000
O	-6.11932000	-2.59751900	-1.57006100
C	-5.46709600	-5.18766100	-0.77009000

H	-3.61955700	-4.09942600	-0.40868500
H	-4.79539200	-3.93047700	0.86794700
H	-5.33331800	-5.27783900	-1.85036400
H	-5.12264800	-6.11204400	-0.29956000
H	-6.53659400	-5.07638400	-0.58148500

HR-cyCH_3 wb97xd/6-31g(d,p) scrf=(iefpcm,solvent=water)

0 1

O	-0.01442500	1.08558400	-0.27699300
C	0.00002000	-0.01905700	0.25065100
N	1.16700100	-0.65072600	0.57686200
N	-1.15017100	-0.69107700	0.55563500
C	1.24421900	-1.88494200	1.17748300
H	2.05880500	-0.17340000	0.29640900
C	-1.19601900	-1.93174700	1.14630500
H	-2.05319100	-0.23747900	0.27256700
N	0.03122500	-2.48306400	1.48585900
O	2.31195800	-2.41931200	1.42242800
O	-2.24929500	-2.50700400	1.35886000
C	0.05306600	-3.82757000	2.08041600
C	0.19728700	-4.92037000	1.02560300
H	-0.88133600	-3.93993200	2.63054000
H	0.88675500	-3.85262400	2.78345800
C	0.17141400	-6.30926500	1.65796900
H	1.13851100	-4.76958300	0.48675300
H	-0.61792100	-4.82298400	0.30008300
H	-0.77600300	-6.48868200	2.17586400
H	0.98076100	-6.42795300	2.38530600
H	0.28879600	-7.08498900	0.89772000
C	-0.06454800	6.16677300	1.85714300
N	3.56429200	0.63779000	-0.35678200
C	3.64833300	1.95773700	-0.59215200
C	4.59251200	-0.14896700	-0.72015100
C	4.75649900	2.54763200	-1.19728700
N	2.51934800	2.69392100	-0.21340100
N	4.43204900	-1.49443900	-0.38891800
C	5.73578300	0.34635900	-1.34907600
C	5.79632900	1.71203900	-1.57653000
H	4.78774200	3.61513700	-1.35035800
C	2.49756400	4.03629600	0.10412700
H	1.66638600	2.15496000	-0.09527900
C	5.19104700	-2.56648900	-0.81464200
H	3.70753700	-1.70823900	0.29266200
H	6.52535700	-0.33053600	-1.63677400
H	6.67238000	2.13489500	-2.05738000
C	1.15702300	4.56811300	0.52397700
O	3.48685500	4.74794600	0.10055700
C	4.73958600	-3.89099700	-0.21445700
O	6.12314300	-2.47830800	-1.59148500
C	-0.04855500	4.03707700	0.06817000
C	1.14409800	5.64616900	1.40996700
C	5.54882900	-5.06911200	-0.73662900
H	3.67113100	-4.02059500	-0.42261800
H	4.81641300	-3.81214600	0.87645500
C	-1.26147100	4.53474000	0.54143300
H	-0.04319000	3.25635400	-0.68072100
H	2.09097800	6.06347100	1.73463000
H	5.43617600	-5.17238500	-1.81802600
H	5.21761500	-5.99655500	-0.26264100

H	6.61215900	-4.93168800	-0.53072300
C	-1.26471800	5.61251200	1.42811400
C	-2.59359300	3.96968000	0.13824200
H	-0.07100600	7.00319300	2.54787600
H	-2.21759900	6.00368900	1.76743900
N	-2.58619800	2.63059100	-0.19425600
O	-3.60090600	4.65542800	0.15757400
C	-3.70244700	1.87313000	-0.57027100
H	-1.71921900	2.11092500	-0.09214800
C	-4.83011100	2.44701500	-1.15454300
N	-3.58710900	0.55207100	-0.35398200
C	-5.85683400	1.59498200	-1.53237500
H	-4.88616300	3.51538200	-1.29312200
C	-4.60399500	-0.25105900	-0.71462500
C	-5.76508500	0.22834500	-1.32317000
H	-6.74702200	2.00560000	-1.99761300
N	-4.41314700	-1.59695500	-0.40256000
H	-6.54389800	-0.46140400	-1.60946500
C	-5.16948200	-2.67665400	-0.81388400
H	-3.66925500	-1.80601100	0.25925200
C	-4.69453600	-3.99663400	-0.22209000
O	-6.11932000	-2.59751900	-1.57006100
C	-5.46709600	-5.18766100	-0.77009000
H	-3.61955700	-4.09942600	-0.40868500
H	-4.79539200	-3.93047700	0.86794700
H	-5.33331800	-5.27783900	-1.85036400
H	-5.12264800	-6.11204400	-0.29956000
H	-6.53659400	-5.07638400	-0.58148500

HR-cy wb97xd/6-31g(d,p) scrf=(iefpcm,solvent=o-dichlorobenzene)

0 1

O	-0.00704500	1.10691400	-0.22085100
C	0.00291900	-0.00431600	0.29890300
N	1.16326900	-0.64320800	0.62178900
N	-1.14594300	-0.67641800	0.59456600
C	1.23651800	-1.88728700	1.20215400
H	2.05251600	-0.17272500	0.34849500
C	-1.19772200	-1.92530300	1.16687200
H	-2.04238400	-0.22392400	0.31557500
N	0.02420000	-2.49025100	1.49346900
O	2.30783700	-2.42021200	1.44096000
O	-2.25931400	-2.49063400	1.37191300
C	0.04024800	-3.84397600	2.06924500
C	0.17850700	-4.92120100	0.99798600
H	-0.89232300	-3.95975900	2.62101800
H	0.87162700	-3.88218500	2.77388200
C	0.15248800	-6.31812000	1.61241600
H	1.11706800	-4.76491900	0.45633900
H	-0.63798800	-4.81225200	0.27579600
H	-0.79328000	-6.50097000	2.13214100
H	0.96442000	-6.44660300	2.33520500
H	0.26508200	-7.08338300	0.84075900
C	-0.04607800	6.10862300	1.92506900
N	3.57786800	0.63702700	-0.34903200
C	3.65188500	1.95040200	-0.61283500
C	4.60074200	-0.15401400	-0.71337800
C	4.74417400	2.53261800	-1.25296700
N	2.52467500	2.68852000	-0.22639300
N	4.44389600	-1.49500600	-0.36157700

C	5.73638400	0.33590700	-1.36188900
C	5.78701900	1.69613200	-1.62460300
H	4.76399000	3.59441700	-1.44324400
C	2.50893900	4.02245400	0.10409900
H	1.67330000	2.14689100	-0.09175600
C	5.16143900	-2.57876000	-0.81710200
H	3.71248700	-1.70226200	0.31693700
H	6.53119300	-0.33771400	-1.64105800
H	6.65402900	2.11231100	-2.12658300
C	1.17313400	4.55332800	0.53745600
O	3.50305100	4.73413900	0.10143000
C	4.70294000	-3.89563500	-0.21111900
O	6.07072200	-2.50167400	-1.62803200
C	-0.03270400	4.04243100	0.05931300
C	1.16163700	5.59897400	1.46216200
C	5.46741400	-5.09301200	-0.75602300
H	3.62706300	-4.00070700	-0.38978200
H	4.81409900	-3.82200500	0.87679500
C	-1.24475000	4.52826000	0.54735700
H	-0.02805900	3.28355500	-0.71178000
H	2.10558300	6.00018600	1.81413200
H	5.32992200	-5.18937200	-1.83552700
H	5.11300100	-6.01104000	-0.28133200
H	6.53804300	-4.99532900	-0.56321300
C	-1.24672800	5.57369600	1.47257700
C	-2.57372700	3.97359600	0.12270200
H	-0.05140000	6.91680600	2.64803800
H	-2.19572800	5.95538200	1.83259300
N	-2.56843400	2.64243600	-0.22021300
O	-3.58088400	4.66664200	0.13581800
C	-3.68707800	1.88986700	-0.60487700
H	-1.70727300	2.11396100	-0.09553200
C	-4.79400600	2.46301100	-1.22801500
N	-3.59140000	0.57472600	-0.35605800
C	-5.82905000	1.61612100	-1.59742400
H	-4.83123100	3.52619300	-1.40707100
C	-4.60834300	-0.22628600	-0.71645200
C	-5.75734500	0.25449800	-1.34838500
H	-6.70661600	2.02536600	-2.08661800
N	-4.43161400	-1.56792300	-0.37753500
H	-6.54568500	-0.42762000	-1.62515900
C	-5.16129200	-2.65393400	-0.80794900
H	-3.68255400	-1.77316400	0.28218600
C	-4.69234500	-3.96716000	-0.20196700
O	-6.09081600	-2.58154200	-1.59621400
C	-5.46224200	-5.16815100	-0.73122800
H	-3.61819100	-4.07260700	-0.38976000
H	-4.79310600	-3.88755600	0.88666100
H	-5.33454600	-5.27257400	-1.81122700
H	-5.10395000	-6.08293800	-0.25319500
H	-6.53102400	-5.06842000	-0.52959400

HR-cyCH_3 wb97xd/6-31g(d,p) scrf=(iefpcm,solvent=o-dichlorobenzene)

0 1

O	-0.83297700	1.27159700	1.40284200
C	-0.31733900	0.16455000	1.33067400
N	0.92404700	-0.11069900	1.86265300
C	1.64956400	0.93854400	2.58490700
C	1.58080900	-1.31064800	1.66316300

H	2.03318700	0.52556000	3.51630100
H	2.47297100	1.29729100	1.96521500
H	0.94797300	1.74298800	2.78901600
N	0.87969700	-2.30227500	1.00092000
O	2.72280600	-1.47638300	2.05989700
N	-0.93915500	-0.88032600	0.70338800
C	1.54577300	-3.58964700	0.74935300
C	-0.39208800	-2.12038300	0.49556000
H	-1.89915900	-0.71285500	0.34332300
C	2.22222800	-3.62932900	-0.61698500
H	2.27107100	-3.73121700	1.55044700
H	0.78032300	-4.36246400	0.82743200
O	-0.99009300	-2.99922500	-0.10349800
C	2.89160500	-4.97898500	-0.85998900
H	1.47356700	-3.43685600	-1.39194000
H	2.95946400	-2.82136900	-0.67393600
H	2.15431300	-5.78767500	-0.86094200
H	3.62999800	-5.20188400	-0.08270800
H	3.40449700	-4.98976000	-1.82461800
C	-1.57780900	6.16187500	1.04061600
N	-3.73866000	-0.43927000	-0.18303900
C	-4.52130000	-1.50118800	-0.44378900
C	-4.29339200	0.78522800	-0.19949200
N	-3.85961900	-2.72779200	-0.42339600
C	-5.88514200	-1.38398600	-0.72661400
C	-5.64528900	1.00204600	-0.46716300
N	-3.39492400	1.82526000	0.06759300
C	-4.40267000	-3.98994600	-0.52300700
H	-2.84520000	-2.69410100	-0.33098100
C	-6.42956600	-0.11122400	-0.73125500
H	-6.46943200	-2.26762500	-0.92796100
H	-6.04641000	2.00230800	-0.45861900
C	-3.65164200	3.17425100	0.05410200
H	-2.48125100	1.54268500	0.41974900
C	-3.36457000	-5.09798000	-0.44131300
O	-5.59591800	-4.21513600	-0.65370900
H	-7.48567800	0.01907400	-0.94311200
C	-2.44930700	4.04853500	0.25791600
O	-4.75795600	3.67021800	-0.10934700
C	-3.97361100	-6.48319700	-0.60103100
H	-2.60118600	-4.91322500	-1.20487700
H	-2.84618700	-5.00445600	0.51963800
C	-1.18152300	3.70057800	-0.20691600
C	-2.64391000	5.28550800	0.87422200
H	-4.46159900	-6.58791000	-1.57289400
H	-3.19582300	-7.24662600	-0.52329500
H	-4.72334500	-6.67407400	0.17012500
C	-0.11226400	4.57905100	-0.04287200
H	-1.03595900	2.76451400	-0.73465500
H	-3.63914600	5.55066200	1.21373500
C	-0.31611800	5.81548700	0.57247200
C	1.25577400	4.27530200	-0.57255700
H	-1.73249900	7.11881100	1.52650800
H	0.52072100	6.49769800	0.67423700
N	1.61471400	2.94843200	-0.52791300
O	1.98083400	5.16030900	-1.00153100
C	2.81810100	2.39392000	-0.99548100
H	1.05333000	2.33388900	0.04717100
C	3.57697300	2.99835800	-1.99857000
N	3.14457100	1.23282900	-0.42334600

C	4.73266700	2.34231600	-2.39630000
H	3.27216400	3.93821700	-2.43176500
C	4.27240200	0.62718300	-0.81717400
C	5.10830000	1.14185700	-1.81072200
H	5.35279600	2.77431400	-3.17476900
N	4.53849800	-0.56067700	-0.12675200
H	6.00545600	0.61369300	-2.09248000
C	5.57914800	-1.43777700	-0.31058400
H	3.89958600	-0.75441300	0.63884700
C	5.55700000	-2.65118500	0.60317900
O	6.45608000	-1.28834700	-1.15002500
C	6.92128000	-2.89510000	1.24717200
H	4.77236000	-2.56376700	1.35861000
H	5.29627300	-3.50412100	-0.03450100
H	6.91303800	-3.83110100	1.81039200
H	7.18178000	-2.08585800	1.93508000
H	7.69712800	-2.95705800	0.48130900

13. References

1. Frisch, M. J.; Trucks, G. W.; Schlegel, H. B.; Scuseria, G. E.; Robb, M. A.; Cheeseman, J. R.; Scalmani, G.; Barone, V.; Mennucci, B.; Petersson, G. A.; Nakatsuji, H.; Caricato, M.; Li, X.; Hratchian, H. P.; Izmaylov, A. F.; Bloino, J.; Zheng, G.; Sonnenberg, J. L.; Hada, M.; Ehara, M.; Toyota, K.; Fukuda, R.; Hasegawa, J.; Ishida, M.; Nakajima, T.; Honda, Y.; Kitao, O.; Nakai, H.; Vreven, T.; Montgomery Jr., J. A.; Peralta, J. E.; Ogliaro, F.; Bearpark, M. J.; Heyd, J.; Brothers, E. N.; Kudin, K. N.; Staroverov, V. N.; Kobayashi, R.; Normand, J.; Raghavachari, K.; Rendell, A. P.; Burant, J. C.; Iyengar, S. S.; Tomasi, J.; Cossi, M.; Rega, N.; Millam, N. J.; Klene, M.; Knox, J. E.; Cross, J. B.; Bakken, V.; Adamo, C.; Jaramillo, J.; Gomperts, R.; Stratmann, R. E.; Yazyev, O.; Austin, A. J.; Cammi, R.; Pomelli, C.; Ochterski, J. W.; Martin, R. L.; Morokuma, K.; Zakrzewski, V. G.; Voth, G. A.; Salvador, P.; Dannenberg, J. J.; Dapprich, S.; Daniels, A. D.; Farkas, Ö.; Foresman, J. B.; Ortiz, J. V.; Cioslowski, J.; Fox, D. J. *Gaussian 09 Revision C.01*, Gaussian, Inc.: Wallingford, CT, USA, 2009.
2. Chai, J.-D.; Head-Gordon, M., Long-range corrected hybrid density functionals with damped atom-atom dispersion corrections. *Physical Chemistry Chemical Physics* **2008,** *10* (44), 6615-6620.
3. Chai, J.-D.; Head-Gordon, M., Systematic optimization of long-range corrected hybrid density functionals. *The Journal of Chemical Physics* **2008,** *128* (8), 084106.
4. McGrath, J. M.; Pluth, M. D., Understanding the Effects of Preorganization, Rigidity, and Steric Interactions in Synthetic Barbiturate Receptors. *J. Org. Chem.* **2014,** *79* (2), 711-719.
5. Francl, M. M.; Pietro, W. J.; Hehre, W. J.; Binkley, J. S.; Gordon, M. S.; DeFrees, D. J.; Pople, J. A., Self-consistent molecular orbital methods. XXIII. A polarization-type basis set for second-row elements. *J. Chem. Phys.* **1982,** *77* (7), 3654-3665.
6. Boys, S. F.; Bernardi, F., The calculation of small molecular interactions by the differences of separate total energies. Some procedures with reduced errors. *Mol. Phys.* **1970,** *19* (4), 553-566.
